# Facet‐dependent Heterogeneous Fenton Reaction Mechanisms on Hematite Nanoparticles for (Photo)catalytic Degradation of Organic Dyes

**DOI:** 10.1002/advs.202508058

**Published:** 2025-09-06

**Authors:** Ping Chen, Duo Song, Tianying Liu, Ying Chen, Yadong Zhou, Micah P. Prange, Tianhu Chen, David Z. Wang, Yatong Zhao, Xiang Wang, Xiaoxu Li, Dunwei Wang, Zihua Zhu, Zheming Wang, Kevin M. Rosso, Xin Zhang

**Affiliations:** ^1^ Physical & Computational Science Directorate Pacific Northwest National Laboratory 902 Battelle Blvd Richland WA 99354 USA; ^2^ Key Laboratory of Nano‐minerals and Pollution Control of Anhui Higher Education Institutes School of Resources and Environmental Engineering Hefei University of Technology No.193 Tunxi Road Hefei 230009 China; ^3^ Department of Chemistry Merkert Chemistry Center Boston College 2609 Beacon Street Chestnut Hill MA 02467 USA; ^4^ Environmental Molecular Sciences Laboratory (EMSL) Pacific Northwest National Laboratory 902 Battelle Blvd Richland WA 99354 USA

**Keywords:** (photo)catalytic degradation, facet‐dependent reactivity, hematite, photo‐fenton reactions, redox chemistry

## Abstract

Although heterogeneous photo‐Fenton reactions on nanoparticulate iron oxides effectively degrade organic pollutants, the underlying surface mechanisms remain debated. Here, we demonstrate how these pathways are modulated by specific hematite crystal facets. To investigate the influence of particle surface structure, methylene blue (MB) adsorption and photodegradation kinetics are examined using facet‐engineered hematite nanoparticles with distinct exposed facets. The results reveal that MB photodegradation strongly depends on both pH and facet orientation. When normalized by surface area, (116) facet shows higher photodegradation activity than those with (104) or (001) facets. This enhanced activity is attributed to favorable electronic structure and surface characteristics, including a smaller optical bandgap, faster charge transfer, and superior H_2_O_2_ decomposition. In contrast, the photodegradation capacity follows (104) 〉 (116) 〉 (001), primarily due to the higher density of surface‐active sites on the (104) facet. These sites promote coupled MB adsorption and degradation, enabling removal of a greater overall quantity of MB. Additionally, under high pH conditions, hematite can degrade MB in the dark, with capacities following (001) ≫ (116) 〉 (104). These findings underscore the critical catalytic role of specific hematite surfaces and advance the understanding of facet‐dependent photoinduced redox chemistry at mineral–water interfaces.

## Graphical abstract

This study reveals how hematite crystal facets govern heterogeneous (photo)‐Fenton degradation of organic dyes. Facet‐dependent differences in H_2_O_2_ decomposition and reactive oxygen species generation are demonstrated, providing mechanistic insights that inform environmental strategies for water purification and pollutant remediation.

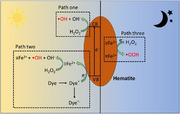

## 1 Introduction

The preponderance of mineral nanoparticles in the environment positions them as important contributors to various geochemical and biogeochemical reactions. Their specific interactions with dissolved compounds and organisms have profound implications for Earth's systems and the functioning of ecosystems as a whole.^[^ ^1^, ^2^, ^3^, ^4^ ^]^ Semiconducting mineral nanoparticles are an important subset that can play a distinct role as photocatalytic substrates for redox processes.^[^ ^5^, ^6^, ^7^ ^]^ This characteristic has also led to their development as designer particles for photocatalytic technologies and applications, such as wastewater treatment,^[^ ^8^, ^9^, ^10^ ^]^ air purification,^[^ ^11^, ^12^ ^]^ and solar energy conversion.^[^ ^13^ ^–^ ^15^ ^]^

Hematite is one such nanoparticle type that exhibits photo‐catalytic semiconducting behavior with significant (bio)geochemical impacts. For example, in the near‐surface environment of aquatic systems, it facilitates the photodegradation of organic matter in a process that often also yields bioavailable ferrous iron.^[^ ^1^, ^5^, ^16^, ^17^ ^]^ The underlying mechanism is thought to be the classical heterogeneous photo‐Fenton process, shown to catalyze the photodegradation of various organic dyes.^[^ ^18^, ^19^, ^20^ ^]^ Electron transfer from photoexcited chromophoric groups in the organic matter to hematite yields Fe(II) on the surface that can activate the production of reactive oxygen species (ROS), enabled either by reaction with electrophilic groups in the organic matter (e.g., carboxylates) or by addition of hydrogen peroxide. Recent studies have demonstrated that hematite is capable of accepting electrons from photoexcited chromophores within dissolved organic matter (DOM), leading to the generation of radicals upon reaction with O_2_ that enable DOM photodegradation through cleavage of aromatic groups.^[^ ^21^ ^]^ Such findings represent the prevailing consensus on the photodegradation of organic components by hematite and underscore its efficacy as a photocatalytic substrate. However, direct band gap excitation of electron‐hole pairs in hematite has also been proposed to play a role,^[^ ^22^, ^23^ ^]^ but its significance is unclear given the poor hole mobility in hematite.^[^ ^24^, ^25^ ^]^ Although a definitive mechanistic model has yet to emerge, the naturally photocatalytic property of hematite nanoparticles has sparked widespread interest in developing applications in solar energy conversion,^[^ ^14^, ^26^, ^27^, ^28^ ^]^ photocatalysis,^[^ ^29^, ^30^, ^31^ ^]^ and environmental sensing.^[^ ^32^, ^33^ ^]^

Because of the diversity of hematite nanoparticle types that have been developed and studied, ranging from uniformly faceted euhedral particles to very rough anhedral particles, there currently is a lack of consensus on what constitutes a bona fide surface structure effect, and why. Dye molecules have been a useful experimental model organic compound. For example, the photodegradation rates of Rhodamine B by different hematite facets have been reported to follow the order of (110) > (012) > > (001).^[^ ^34^ ^]^ Chan et al. linked this to differences in surface atom arrangements and the number and type of hydroxyl groups bonded to terminal underlying Fe atoms.^[^ ^18^ ^]^ Similarly, Huang et al. concluded the critical factor is the extent of surface Fe coordination,^[^ ^20^ ^]^ and Ni et al. found that higher catalytic activity on hematite (012) over (001) is due to more electron accepting sites and reactive oxygen species.^[^ ^29^ ^]^ But Zong et al.^[^ ^19^ ^]^ and Zhou et al.^[^ ^35^ ^]^ reported that facet‐specific differences in photodegradation behavior were controlled by surface structure impacts on organic adsorption. Besides, it is noteworthy that while the decomposition of hydrogen peroxide has long been recognized as a crucial step in heterogeneous photo‐Fenton systems,^[^ ^36^ ^]^ the influence of crystal facet regulation of the efficiency of hydrogen peroxide decomposition remains uncertain. Finally, Electron transfer at hematite/water interfaces is also controlled in part by crystal facet expression.^[^ ^37^, ^38^, ^39^ ^]^ This arises because different low‐index facets on hematite have different surface atomic structures and consequently different electrostatic properties, such as pH‐dependent surface potentials.^[^ ^40^, ^41^ ^]^ Therefore, additional careful facet‐specific studies are needed to help reconcile these contrasting views.

In this Article, we synthesized three types of hematite nanoparticles that differ primarily in their dominant facet expression, namely (001), (104), and (116) dominated particles, and studied their (photo)‐Fenton activities with respect to methylene blue (MB) dye. We selected these hematite particles because: (1) they could be synthesized using simple hydrothermal methods without the aid of surfactants; (2) one crystallographic facet is highly dominant (>88%) in each sample; (3) (116) and (104) are high surface energy facets and as such have remained relatively unexplored in previous studies. Our observations revealed two pathways of MB photodegradation, one involving direct band gap photoexcitation of electron‐hole pairs in the heterogeneous photo‐Fenton system, and another pathway of dark degradation. Given its importance, we thoroughly explore the (photo)‐Fenton chemistry involved. Our findings reinforce the picture that the impact of hematite nanoparticles on the photo‐induced redox chemistry at the Earth's surface goes beyond simple mineral phase dependence,^[^ ^5^ ^]^ but also entails morphology and, therefore, particle history dependence.

## 2 Results and Discussion

### 2.1 Hematite Nanoparticle Preparation and Characterization

**Figure 1:**
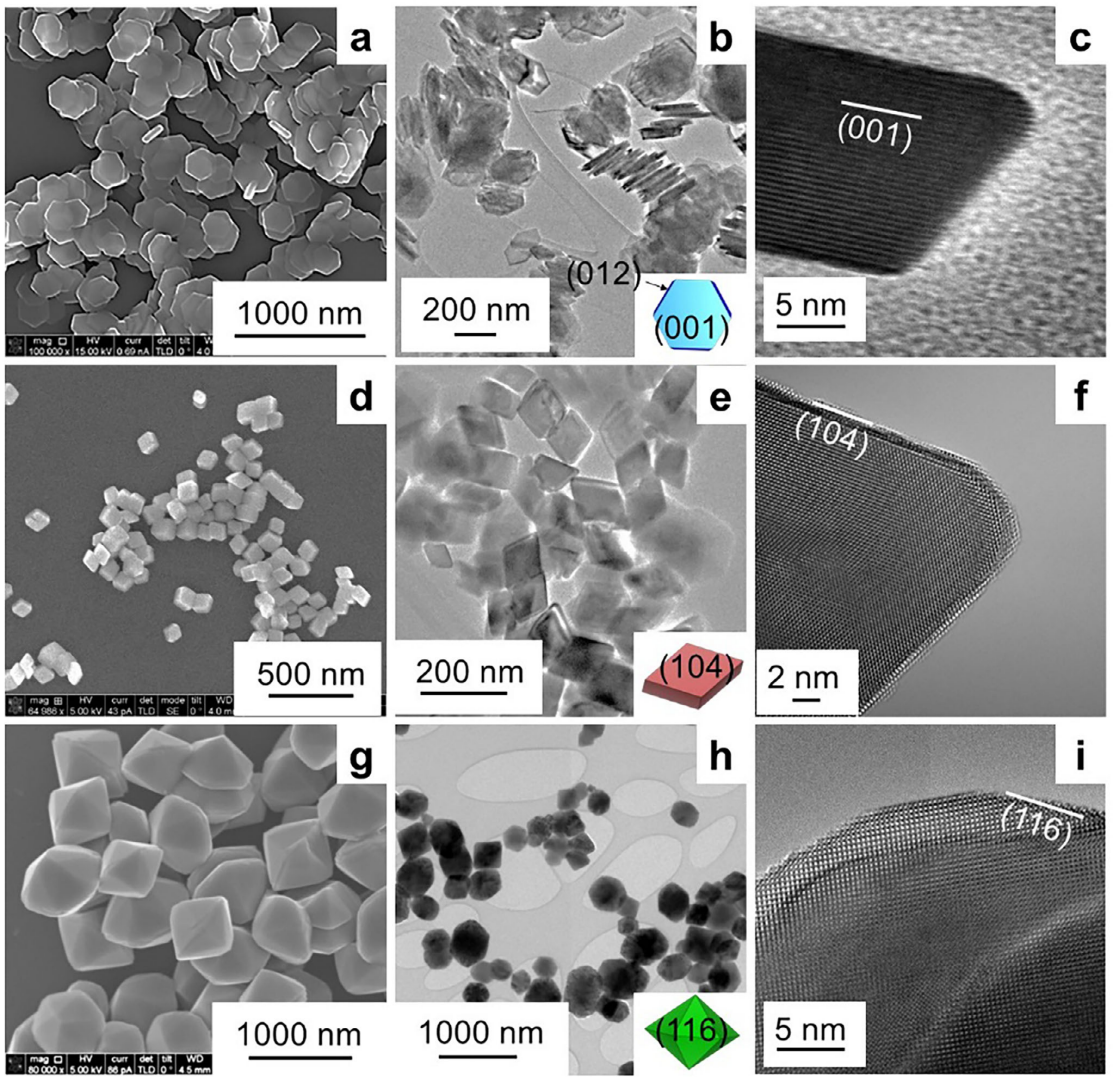
The morphologies of the three sets of initial as‐synthesized hematite nanoparticles. a–c) Hexagonal‐shaped nanoplatelets with 2 (001) basal facets and 12 (012) edge facets. d–f) Rhombic‐shaped nanoparticles with 6 (104) facets. g–i) Hexagonal bipyramid‐shaped nanoparticles with 12 (116) facets.

Hematite nanoparticles with different morphologies (**Figure** **1**) were prepared using iron (III) chloride hexahydrate as an iron source under hydrothermal or solvothermal conditions (see **Methods** for details).^[^ ^42^, ^43^ ^]^ The morphology and structure of as‐synthesized hematite nanoparticles were first characterized in detail using X‐ray diffraction (XRD), scanning electron microscopy (SEM), and high resolution (scanning) transmission electron microscopy (HR S/TEM). As shown in Figure S1 (Supporting Information), the XRD patterns indicated that all samples were pure hematite with high crystallinity. All diffraction patterns matched well with rhombohedral α‐Fe_2_O_3_ (International Centre for Diffraction Data 33–0664).^[^ ^44^ ^]^ The sharp peaks located at the 2θ angles of 33.3°, 35.7°, and 54.2° were assigned to (104), (110), and (116) lattice planes, respectively.

SEM and TEM revealed highly uniform particle sizes, shapes, and high crystallinity for each of the three types of morphologically distinct hematite nanoparticles (Figure 1). Figure 1a–c show the hexagonal‐shaped hematite nanoplatelets defined by two dominant (001) basal facets and twelve (012) edge facets, for which the surface area ratio of (001) to (012) is ≈9:1.^[^ ^19^, ^45^ ^]^ The 3D schematic inserted in Figure 1b illustrates the morphology of hexagonal‐shaped hematite particles, where light yellow represents (001) planes and deep yellow represents (012) planes. The HRTEM micrograph in Figure 1c shows the high crystallinity of the nanoplatelets. Similar high‐quality synthesis results were achieved for the other two sets of hematite particles. The second set consisted of rhombic crystals enclosed by six (104) facets shown in Figure 1d–f. The third set consisted of hexagonal bipyramidal particles enclosed by twelve (116) facets as shown in Figure 1g–i. Additional characterization results, including specific surface area (SSA), bandgap, and the density of surface‐active sites, are listed in Table S1 (Supporting Information).

### 2.2 Heterogeneous Photo‐Fenton (Visible Light) Reactivity Measurements

**Figure 2:**
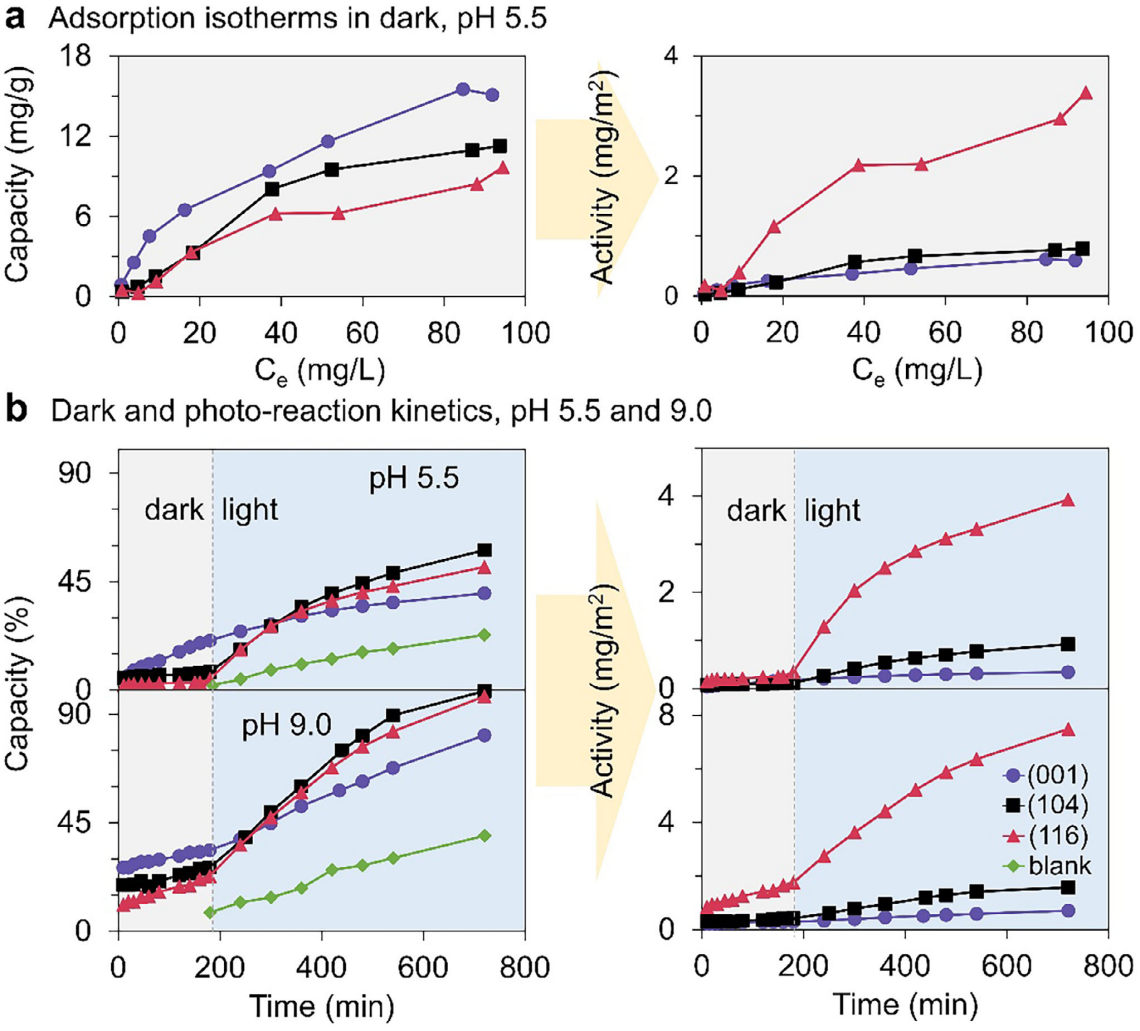
MB adsorption isotherms a), MB dark and photo‐reaction kinetics b) on three different hematite particles.

To examine the MB photodegradation of three types of nanoparticles, we first measured MB adsorption isotherms at pH 5.5 (The pH monitoring results were shown in Table S2, Supporting Information) in the dark with the present of H_2_O_2_ (initial concentration: 0.87%, w/w). Here, we introduce two terms to describe the adsorption and (photo)degradation behavior of methylene blue (MB) on hematite: capacity and activity. Capacity refers to the total amount of MB removed per unit mass of hematite (e.g., mg/g or overall removal percentage), whereas activity describes the intrinsic adsorption/photodegradation efficiency normalized to surface area (e.g., mg/m^2^). Based on these definitions, the MB adsorption capacity followed the order (001) > (104) > (116) (**Figure** **2**a). In contrast, the activity displayed a different trend, with (116) > (104) ≈ (001). The adsorption of MB on various hematite nanoparticles was evaluated using three different models: the Freundlich isotherm model, the Langmuir isotherm model and the Temkin isotherm model. Among these models, the Langmuir isotherm provided a better fit for all three types of hematite nanoparticles, indicating single‐layer adsorption of MB on the hematite nanoparticle surfaces.^[^ ^46^, ^47^ ^]^ Further details and results regarding the fitting can be found in Figure S2 and Tables S3, Supporting Information).

Additionally, we investigated MB adsorption kinetics in the presence of H_2_O_2_ (initial concentration: 0.87%, w/w) during the dark period (Figure 2b) and analyzed the data using pseudo‐first‐order and pseudo‐second‐order models, as described in the methods section. The kinetics data at pH 5.5 were best fitted by the pseudo‐second‐order model. Notably, the (104) samples exhibited the highest adsorption rate of ≈2.524 g/mg/min, followed by (116) (≈0.540 g/mg/min) and (001) (≈0.043 g/mg/min). However, both the pseudo‐first‐order and pseudo‐second‐order models produced poor fittings to the data at pH 9.0, as described in Figure S3, and Table S4, Supporting Information). These observed differences in the absorption kinetics at the two pH values suggest that the mechanism for MB adsorption strongly depends on pH.

After reaching the equilibrium adsorption of MB on hematite nanoparticles in the dark, the samples were subjected to illumination under visible light by using a 200 W xenon arc lamp. At pH 5.5, the photodegradation capacity (Figure 2b, left), followed the order (104) > (116) > (001), while the photodegradation activity, (Figure 2b, right), followed the order (116) > (104) > (001). Similar trends were observed at pH 9.0 (Figure 2b). Throughout the photodegradation experiments, the pH of the solution was monitored, which remained relatively stable. Furthermore, no obvious dissolved iron was released from the nanoparticles during the process (Figure S4, Supporting Information). To analyze the photodegradation kinetics, we employed the Langmuir‐Hinshelwood (L‐H) pseudo‐first‐order model, which provided a good fit for the experimental data.^[^ ^19^, ^20^ ^]^ The rate constants obtained from the fitting are reported in Table S5 and Figure S5 (Supporting Information). Specifically, for the (001), (104), and (116) samples at low pH (5.5), the L‐H pseudo‐first‐order model yielded rate constants of 0.0005, 0.0015, and 0.0012 h^−1^, respectively, effectively describing the data over the duration of the experiments. Conversely, at high pH, only the data within the first 9 h of the reaction could be well‐fitted using the L‐H pseudo‐first‐order model, with rate constants of 0.0020, 0.0053, 0.0042 h^−1^ for (001), (104), and (116) samples, respectively. The failure of the L‐H model fitting at later times can be attributed to the low concentration of MB remaining in the solution after 540 min, as it was nearly consumed in the photodegradation experiments for the (104) and (116) samples. Notably, the (104) sample exhibited the highest photodegradation rate constants at both pH values, indicating its superior performance in the degradation of MB.

### 2.3 Reactive Oxygen Species Observations from In Situ Electron Paramagnetic Resonance (EPR) Spectroscopy

In situ EPR spectroscopy studies using the spin‐trapping molecule DEPMOPO (details described in the methods section) were performed to determine the role of ROSs. Results (Figures S6–S8, Supporting Information) showed only two ROSs that participate in the reaction: DEPMOPO‐OOH and DEPMOPO‐OH, observed at pH 5.5 and 9.0, respectively. The DEPMOPO‐OOH adduct was identified by the twelve resolved peaks (Figure S6, Supporting Information).^[^ ^48^ ^]^ We assigned these EPR signals to DEPMOPO‐OOH not DEPMOPO‐OOR^[^ ^49^ ^]^ based on previous studies of heterogeneous photo‐Fenton reactions.^[^ ^50^, ^51^, ^52^, ^53^ ^]^ Additionally, the ∙OOR radical usually forms in an organically associated system,^[^ ^54^, ^55^ ^]^ so it is more likely to be DEPMOPO‐OOH in a pure hematite and hydrogen peroxide system, and the same applies to the ternary system involving hematite, H_2_O_2_, and MB. The EPR signals of the pH 9.0 samples consist of two sets of four singlets with an intensity ratio of 1:2:2:1,^[^ ^56^ ^]^ unambiguously supporting the DEPMOPO‐OH generation in the photocatalytic process (Figure S7, Supporting Information). A tiny amount of DEPMOPO‐OOH was also seen at pH 9.0. Based on EPR results, self‐decomposition of aqueous H_2_O_2_ into ∙OH occurred at pH 9.0 irrespective of light exposure, with accelerated decomposition in the presence of hematite. However, at pH 5.5, H_2_O_2_ remained stable in both dark and light conditions. Only during the photoreaction, in conjunction with hematite, did H_2_O_2_ decompose to produce ∙OOH.

**Figure 3:**
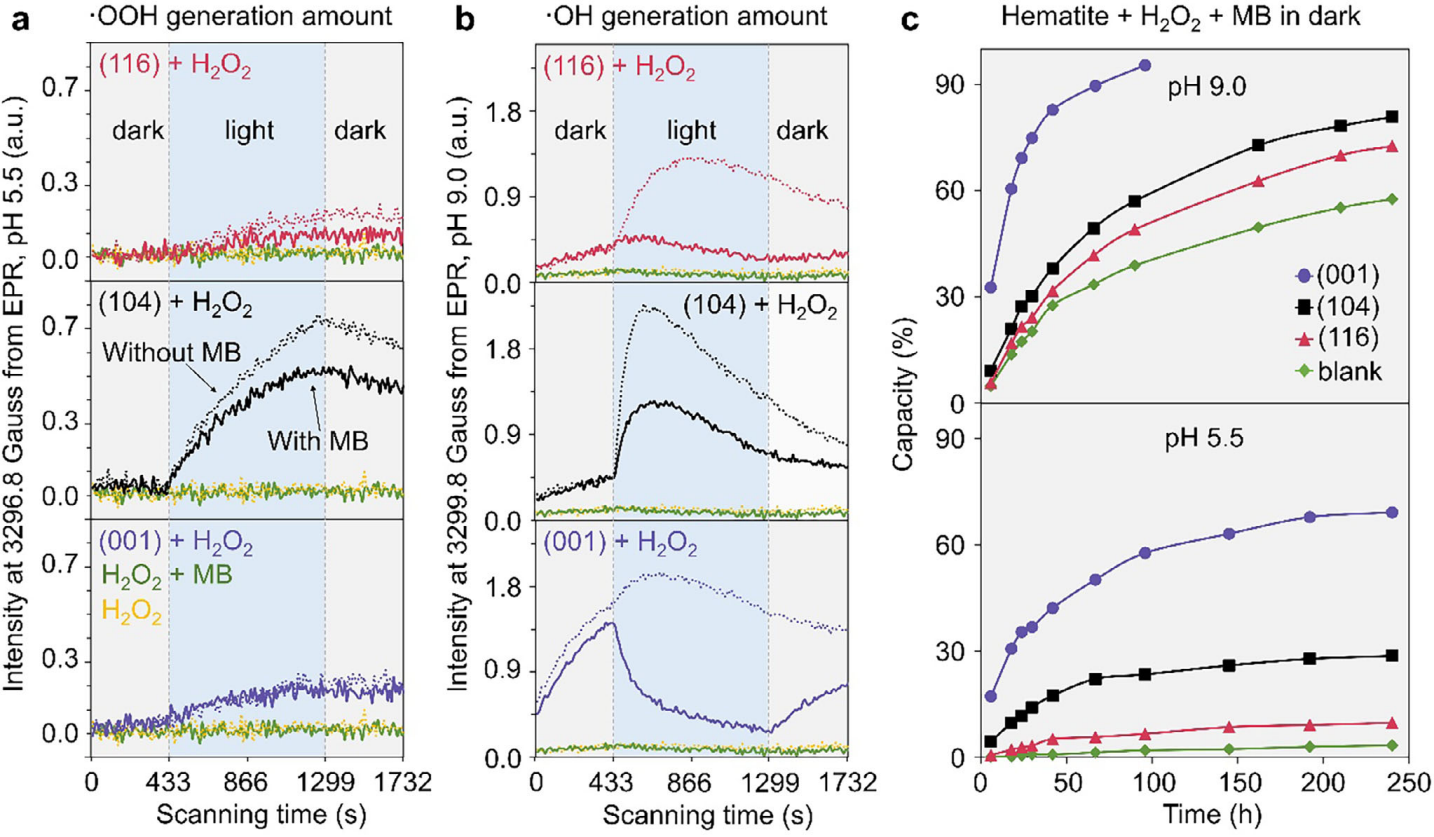
Corresponding intensity changes at 3296.8 Gauss a, DEPMPO‐OOH, pH ≈5.5) and 3299.8 Gauss b, DEPMPO‐OH, pH 9.0) from measured DEPMPO‐trapped EPR signal changes in aqueous solutions as a function of time. c) MB degradation capacity in the dark as a function of long time.

Using the EPR signal intensity at 3296.8 Gauss as a measure of the concentration of ∙OOH as a function of time in the pH 5.5 experiments (**Figure** **3**a), we observed that the concentration of ∙OOH increased linearly during illumination (433 s to 866 s) then immediately decreased upon turning the lamp off at 866 s. No ∙OOH was detected in the dark time (0 to 433 s). Hence, ∙OOH is a photogenerated species that decays in the absence of light in these experiments. The (104) samples generated much more ∙OOH than the samples exposing the other two facets, which showed similar production of ∙OOH. Also, even though the MB removal capacity by the three hematite samples was about the same (Figure S9a, Supporting Information), ∙OOH consumption upon the addition of MB was much more pronounced in the (104) samples, while the (001) barely consumed any ∙OOH, and the (116) sample exhibited intermediate behavior.

A similar analysis was performed using the 3299.8 Gauss feature at pH 9.0 to monitor ∙OH concentration (Figure 3b). Interestingly, ∙OH generation was more complicated than ∙OOH. In the dark, (001) yielded much more ∙OH than the other two facets. Illumination diminished the production of ∙OH on (001). Under illumination, (104) produced the most ∙OH, followed by (116) and (001).

MB degradation in the dark at high pH, especially by (001), was of interest. We ran additional experiments (Figure 3c) to verify the long‐time degradation without illumination (heterogeneous Fenton reaction). The results showed that over 95% MB was degraded by (001) at the first time, and 92.1% degradation capacity was reached after recycling for three times (Figure S9b, Supporting Information). We also heated (001) terminated hematite under air up to 300 and 500 °C, respectively, to reduce potential effects of the crystal's surface defects, Fe^2+^, and organics,^[^ ^57^ ^]^ but MB degradation was not substantially affected by either treatment (Figure S9c, Supporting Information).

### 2.4 Intermediate Product Observations from Time‐of‐Fight Secondary Ion Mass Spectrometry (ToF‐SIMS)

**Figure 4:**
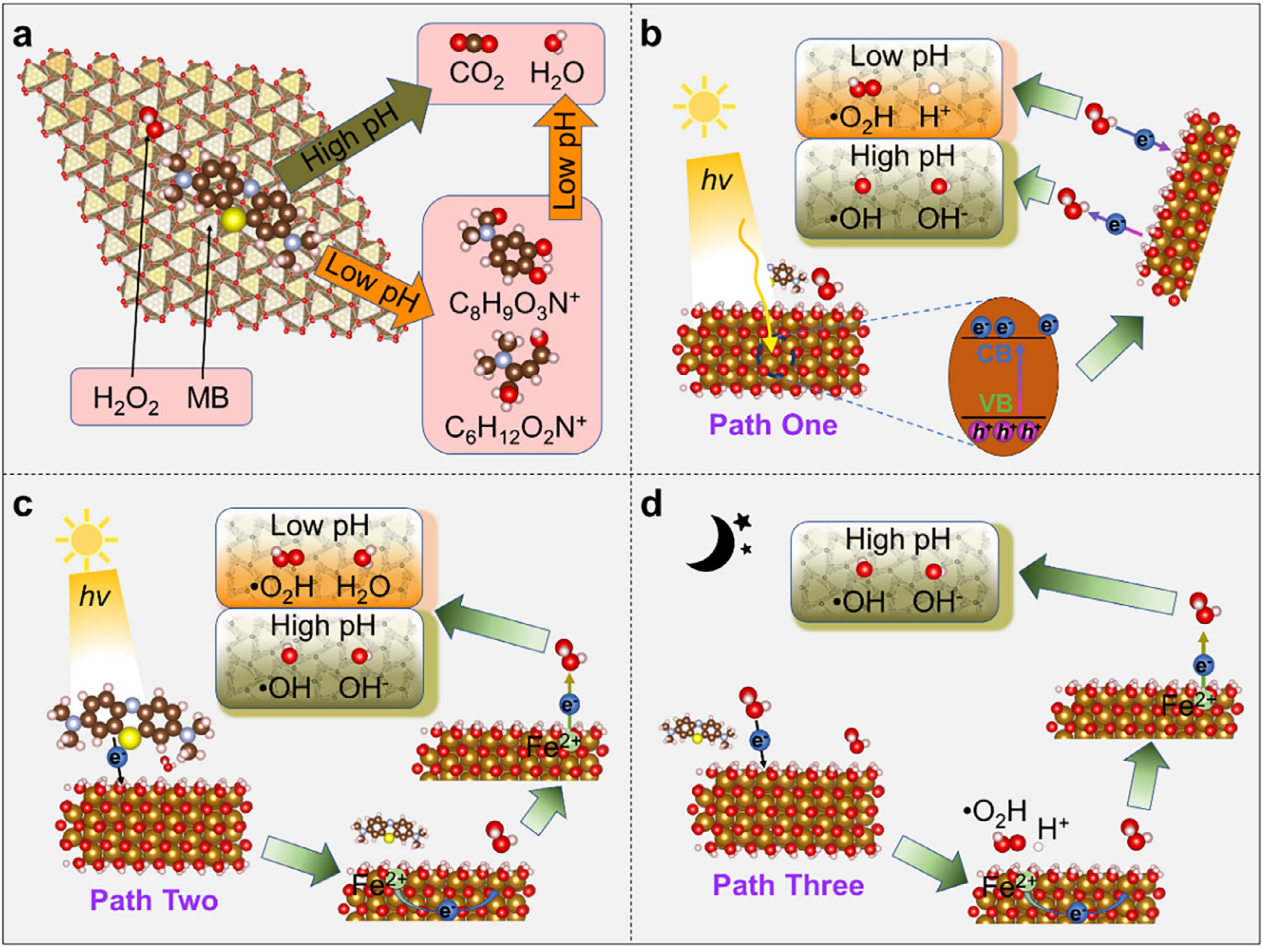
a) Proposed MB degradation pathway during the (photo)catalysis process. Conceptual schematic representation of the photocatalyzed degradation mechanism: Path One b), Path Two c), and the dark catalyzed degradation mechanism of Path Three (c).

Ex situ ToF‐SIMS was performed to gain surface molecular information of hematite at different reaction times and help to identify reaction intermediates and also the possible degradation routes. In the TOF‐SIMS spectra (Figures S10 and S11, Supporting Information), several peaks were associated with MB^+^ molecules (*m/z* = 284 (C_16_H_18_N_3_S^+^)), and their characteristic fragments (analogs), such as m/z 270 (C_15_H_16_N_3_S^+^), *m/z* 268 (C_15_H_14_N_3_S^+^), *m/z* 254 (C_14_H_12_N_3_S^+^), and *m/z* 240 (C_13_H_10_N_3_S^+^). At low pH of 5.5, only (116) showed adsorbed MB^+^‐associated peaks (180 min, Figure S10c, Supporting Information). This is due to the low MB coverage on (001)/(104) and is consistent with the adsorption experiments discussed above (Figure 2b). (116) samples that had been illuminated for 720 min produce m/z spectra in which the MB^+^‐associated peaks were replaced by the peaks at *m/z* 130 (C_6_H_12_O_2_N^+^) and *m/z* 167 (C_8_H_9_O_3_N^+^), which indicates the photodegradation of MB^+^ and analogs will initially decompose into intermediate compounds (C_6_H_12_O_2_N^+^, C_8_H_9_O_3_N^+^) before yielding the final products of CO_2_ and H_2_O. Similar peaks at *m/z* 130 and/or *m/z* 167 were also observed in the illuminated (001) and (104) particles. At a high pH of 9.0, all three types of hematite showed adsorbed MB^+^ and analogs peaks, but no intermediates were recorded in the range m/z = 100 to 200 after illumination, indicating the degradation was more direct and efficient at high pH (**Figure** **4**a). This suggests that ∙OOH is less reactive than ∙OH^[^ ^58^ ^]^ and allows intermediates in the degradation reaction to persist long enough to contribute to the TOF‐SIMS data. Furthermore, the presence of adsorbed intermediates can impede the progress of ongoing reactions, which is consistent with smaller observed photodegradation rate constants at low pH compared to high pH.

### 2.5 Steps Controlling MB (Photo)‐Degradation

From the EPR results, we can draw two paths for MB photodegradation (**Path One** and **Path Two**) and one path for MB degradation in dark (**Path Three**) (Figure 4b–d). Photogenerated carriers of either sign, originating from the hematite lattice, can react with amphoteric H_2_O_2_ to produce radicals through Equations (1) and (2) (Figure 4b).^[^ ^59^, ^60^ ^]^

(1) $$H_{2}O_{2}+h^{*}==\mathrm{OOH}+H^{*}\mathrm{acid}$$

or

(2) $$H_{2}O_{2}+e^{-}==\mathrm{OH}+OH^{-}\mathrm{base}$$

We label this as **Path One**. Another possibility is that the photo‐absorption occurs in the MB itself followed by electron transfer to the hematite substrate with subsequent ˙OH/∙OOH production (Figure 4c):^[^ ^18^, ^19^ ^]^

(3) $$\mathrm{MB}+\mathrm{hv}\left(visible\;light\right)==MB^{*}$$

(4) $$\equiv Fe^{3+}+MB^{*}==\equiv Fe^{2+}+MB^{*+}$$

(5) $$\equiv Fe^{2+}+2H_{2}O_{2}+H+==Fe^{3+}+\mathrm{OOH}+2H_{2}O\mathrm{acid}$$

or

(6) $$\equiv Fe^{2+}+2H_{2}O_{2}==Fe^{3+}+\mathrm{OH}+OH^{-}\mathrm{base}$$

We call this sequence **Path Two**, also known as the classical heterogeneous Fenton reactions. **Path Three** does not require photoactivation. In this path, hematite oxidizes H_2_O_2_ to form ∙OOH in a first step (Equation 7).^[^ ^61^, ^62^ ^]^ The resulting lattice Fe^2+^ then reduces another hydrogen peroxide molecule, producing ∙OH (Equation 8). This is the path (Figure 4c) to which the observed MB degradation in the dark can be attributed to.

(7) $$\begin{array}{ccc} \equiv Fe^{3+}+H_{2}O_{2} & & ==\equiv \mathrm{Fe}\text{…}\mathrm{OO}H^{2+}+H^{+}\\ & & ==\equiv Fe^{2+}+\mathrm{OOH}+H^{+} \end{array}$$

(8) $$\equiv Fe^{2+}+H_{2}O_{2}==\equiv Fe^{3+}+\mathrm{OH}+OH^{-}$$

**Figure 5:**
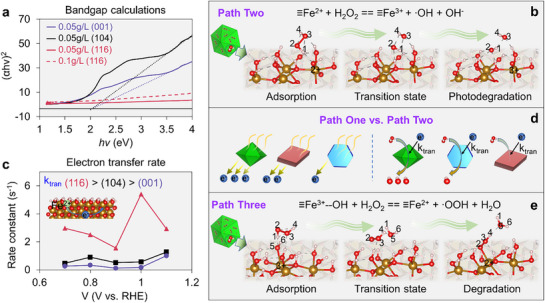
a) the (αhn)^2^ versus hv curve of the three hematite nanoparticles from UV–vis spectra. b) different states of the Equation 10 in Path Two from the NEB minimum energy pathway of (116). c) Charge transfer rate constants extracted from IMPS data at different applied potentials for (104), (001), (116). d) comparison of Path One and Path Two in the MB photodegradation. e) different states of Equation 11 in Path Three from the NEB minimum energy pathway of (116).

A prerequisite of **Path One** (and photo‐redox chemistry generally) is photoelectron generation. To assess variations in photocarrier generation between the samples, the optical band gap was measured using UV‐vis absorption experiments on nanoparticle suspensions. These values were supplemented with estimates from density functional theory (DFT) calculations using a PBE+U approach as described in the methods section. While the absolute value of the band gap from the DFT calculations is expected to be sensitive to the values chosen for the Hubbard parameters (*J* = 1 eV and *U* = 4 eV), the differences among slab models using the same approximation are meaningful. We find values in the range 1.8‐2.2 eV in the order (116) < (104) < (001) for the band gaps (**Figure** **5**a; Figure S12 and Table S1, Supporting Information). This suggests that more electron‐hole pairs are generated near (116) surfaces of hematite under solar illumination compared to other facets.

Charge neutral 1 × 1 slab models of the hematite surfaces studied here, described in the **Methods section**, were used in DFT calculations. In these models, we calculated minimum energy pathways and associated barriers and transition states using nudged elastic band methods for the reductive dissociation of hydrogen peroxide (Equation 6). The resulting barriers were 0.018 eV, 0.191 eV, and 0.390 eV for (116), (001), (104), respectively (Figure S13, Supporting Information). Thus, H_2_O_2_ degradation proceeds more readily on the Fe^2+^/hematite (116) surface. As shown in Figure 5b, the final step of this reaction is the breaking of O2‐O3 bonding and oxidation of Fe^2+^. These calculations were supported with further experiments in which MB degradation by hematite nanoparticles exposed to Fe^2+^_aq_ was monitored (Figure S14b, Supporting Information). These tests showed that Fe^2+^ adsorbed (116) nanoparticles had the highest MB removal capacity. The disparity in energy barriers may also imply the significance of surface Fe coordination in the cycling of Fe(II)/Fe(III), as suggested by previous studies.^[^ ^18^, ^20^ ^]^

Broadly speaking, adsorption must occur before (photo)degradation. Therefore, the number of active adsorption sites can affect the photodegradation kinetics. For example, the (001) terminated hematite studied here has an adsorption site density of 1.13 × 10^20^ site/g, which is 22.6 times larger than (116), hence (001) could adsorb more H_2_O_2_ or MB (Figure S15 and Table S1, Supporting Information), and more H_2_O_2_ can take part in the **Path One** or more e^−^ in **Path Two** can be generated by illuminated MB.

For the photodegradation, the electron transfer rate is another factor that needs to be considered.^[^ ^63^, ^64^, ^65^ ^]^ For this purpose, data measured by IMPS are presented in Figure 5c (Figure S16, Supporting Information) showing the charge transfer rate constants (*k*_tran_) of (001) and (104) hematite are comparable across the entire voltage range, in which the *k*_tran_ of (104) are slightly larger than *k*_tran_ of (001), indicating a faster complex charge transfer processes from the photoelectrode to electrolyte. More interestingly, the *k*_tran_ of (116) hematite is much larger (about one order of magnitude) than that of (001) and (104) hematite, highlighting the faster charge transfer property of (116) hematite. However, both **Path One** and **Path Two** involve electron transfer, despite fundamentally different mechanisms of electron generation: **Path One** relies on photogenerated electrons originating from hematite, whereas **Path Two** involves electron donation from photoexcited MB. Regardless of their origin, we consider that the electrons follow the same transport behavior within a given hematite particle, as their mobility is governed by the material's intrinsic electronic properties. Therefore, the electron transfer rates measured by IMPS can be appropriately used to characterize charge transport in both pathways, since the charge carriers in both cases are electrons.

From our observations, we can conclude that photoelectron generation, charge transfer, and surface‐active sites are involved in **Path One**. On the other hand, charge transfer from MB photosensitization, ≡Fe^2+^…H_2_O_2_ degradation, and surface‐active sites are key factors in **Path Two**. Furthermore, we can observe that the photodegradation activity is determined by processes such as photoelectron generation, charge transfer, and H_2_O_2_ degradation, while the final photodegradation capacity is significantly controlled by the presence of surface‐active sites. Therefore, the MB photodegradation activity of the hematite nanoparticles follows the trend (116) > (104) > (001) for **Path One**, and (116) > (001) > (104) for **Path Two** (Figure 5d). This is because the (116) facet exhibits superior performance in photoelectron generation, charge transfer, and ≡Fe^2+^…H_2_O_2_ degradation. In contrast, the (104) and (001) facets show only moderate advantages in individual steps of the process. Particularly, our DFT calculations indicate that the energy barrier associated with Equation 6—representing the key step in **Path Two**—is significantly higher on the Fe^2+^/hematite (104) surface than on the (001) surface. Although the overall photodegradation efficiency of MB on the (001) facet is relatively low, it results from the combined contributions of both **Path One** and **Path Two**. In contrast, the high barrier on (104) suggests that the ≡Fe^2+^…H_2_O_2_ degradation step in **Path Two** is unlikely to occur on this facet. This mechanistic interpretation is further supported by the ∙OOH concentration profiles shown in Figure 3a. For the (104) particles, a pronounced difference in ∙OOH levels with and without MB is observed, indicating the operation of **Path One** alone. Conversely, for the (116) and (001) particles, only minor differences are observed under the same conditions, implying that both **Path One** and **Path Two** are active, with **Path Two** likely compensating for ∙OOH consumption via continuous generation of ∙OOH through photoexcited MB. In summary, the (116) facet supports both **Path One** and **Path Two**, the (104) facet is limited to **Path One**, and the (001) facet exhibits low activity in both. Consequently, the photodegradation rate constants for (001) and (116) reflect contributions from both pathways, while that of (104) arises solely from **Path One**. However, due to differences in surface‐active site density (Table S1, Supporting Information), the final photodegradation capacity follows the order: (104) > (116) > (001). One limitation of this study is the inability to quantitatively evaluate the relative contributions of **Path One** and **Path Two** within individual hematite samples, due to constraints in the available data.

Therefore, we argue that radical kinetics derived from EPR measurements in the presence of H_2_O_2_, hematite, and MB do not accurately reflect the actual radical generation rates and thus cannot be directly correlated with the observed MB degradation rate constants. In such systems, the EPR signal represents the residual radical concentration—i.e., the amount remaining after partial consumption by MB—rather than the total radicals produced. As a result, these measurements inherently underestimate the extent of radical formation and do not directly correspond to the apparent degradation kinetics. Similarly, EPR measurements conducted with only H_2_O_2_ and hematite omit the contribution of **Path Two**, in which photoexcited MB donates electrons to the hematite surface, further generating radicals. This omission leads to an incomplete picture of radical dynamics, except in the case of the (104) facet. For this facet, radical generation is primarily driven by photogenerated electrons reacting with H_2_O_2_ via **Path One**, making EPR signals a more accurate reflection of total radical production.

Although (001) performed poorly in the MB photodegradation, we were surprised to observe MB degradation in the absence of light at high pH. DFT calculations indicated the energy barriers of Equation 7 were 0.066, 0.095, 0.153 eV for (116), (104), (001), respectively (Figure S17, Supporting Information), which meant (116) is the best candidate for the reaction. But the values in this case are nearly isoenergetic, suggesting the prospect that the reaction is nearly equally favorable on these different hematite nanoparticles. Therefore, the more H_2_O_2_ adsorbed on the surface could lead to more involvement in the reaction, and thus produce a high MB degradation in the order of (001) > (104) > (116). This may also explain why the adsorption kinetics fittings of MB at high pH were poor. The simulations revealed details of the mechanism for degradation (Figure 5e): the adsorbed H_2_O_2_ can donate its surplus e^−^ to hematite to complete the Fe^3+^ reduction, leading to its decomposition into H^+^ and OOH radical. H^+^ combines with surface ‐OH to form water molecules, detaching from the surface and generating free water. Meanwhile, the resulting OOH radical will occupy the vacancy. The fact that low DEPMPO‐OOH signal was detected by EPR may be due to most of the ‐OOH being incorporated into oxygen vacancies at the surface. However, the dark degradation is Fe^2+^ limited, because in the classical Fenton reaction,^[^ ^66^ ^]^ k for Equation (11) is ≈0.001 ≈0.01 M^−1^ s^−1^, and much smaller than Equation (12) (≈63–76 M^−1^ s^−1^), so we expect the photoinduced reaction to be faster.

## 3 Conclusion

The findings of this study have significant environmental implications, particularly in advancing sustainable and efficient water treatment technologies. Compared with Fe_3_O_4_‐ or TiO_2_‐based catalysts (Table S6, Supporting Information), hematite‐based catalysts demonstrate strong potential for facilitating heterogeneous photo‐Fenton reactions. They can be synthesized cost‐effectively and offer an efficient strategy for removing organic pollutants, including dye contaminants, from wastewater. By understanding facet‐dependent reactivity, hematite catalysts can be optimized to enhance degradation capacity while reducing the reliance on excessive chemical additives like H_2_O_2_, thereby minimizing secondary pollution. This study identifies two pathways for heterogeneous photo‐Fenton reactions and one for heterogeneous Fenton reactions, all dictated by specific hematite facets. High‐energy facets, such as (116), improve electron transfer, promote H_2_O_2_ decomposition, and enhance overall catalytic performance, making them well‐suited for oxidation‐based wastewater treatment. Future research should focus on optimizing the (116) facet's size to maximize surface effects, improve recyclability, and further reduce secondary pollutants. However, surface‐active site density is equally critical. The (001) facet exhibits higher surface site amounts than (104) and (116), achieving greater MB degradation capacity at high pH in the absence of light. This suggests that the most effective catalyst design must account for both energy considerations and reaction conditions, rather than focusing solely on high‐energy facets. For real‐world wastewater treatment applications, the optimization of photocatalysts must account for additional practical considerations, including catalyst recovery, long‐term stability under variable pH and ionic strength conditions, and system‐specific operational constraints. To address these challenges, we propose immobilizing facet‐engineered hematite nanoparticles onto magnetic substrates to enable efficient magnetic separation and recovery. Furthermore, future research should investigate surface functionalization approaches to enhance mineral stability across a wider pH range, as well as the design of composite materials that integrate high catalytic activity with improved mechanical and chemical durability. These strategies will be critical for translating our fundamental insights into scalable, robust, and sustainable water treatment technologies. All in all, by deepening our understanding of facet‐dependent catalytic behavior, this study contributes to the rational design of highly efficient, durable, and environmentally sustainable catalysts for scalable and practical solutions in industrial and municipal wastewater treatment.

## Conflict of Interest

The authors declare no conflict of interest.

## Supporting information



Supporting Information

## Data Availability

The data that support the findings of this study are available from the corresponding author upon reasonable request.
