# Supplementary material for: Facet‐dependent Heterogeneous Fenton Reaction Mechanisms on Hematite Nanoparticles for (Photo)catalytic Degradation of Organic Dyes
Source: Adv Sci (Weinh). 2025 Sep 6;12(43):e08058. doi: 10.1002/advs.202508058 (PMC12631832; doi:10.1002/advs.202508058)
Supplement: Supplementary file 1 — Supporting Information [file ADVS-12-e08058-s001.docx]

Facet-dependent Heterogeneous Fenton Reaction Mechanisms on Hematite Nanoparticles for (Photo)catalytic Degradation of Organic Dyes

Ping Chen,^[a, b]^ Duo Song,^[a]^ Tianying Liu,^[c]^ Ying Chen,^[a]^ Yadong Zhou,^[d]^ Micah P. Prange,^[a]^ Tianhu Chen,*^[b]^ David Z. Wang,^[c]^ Yatong Zhao,^[a]^ Xiang Wang,^[a]^ Xiaoxu Li,^[a]^ Dunwei Wang,^[c]^ Zihua Zhu,^[d]^ Zheming Wang,^[a]^ Kevin M. Rosso,*^[a]^ Xin Zhang*^[a]^

[a] Dr. P. Chen, Dr. D. Song, Dr. Y. Chen, Dr. M. P. Prange, Dr. Y. Zhao, Dr. X. Wang, Dr. X. Li, Dr. Z. Wang, Dr. K. M. Rosso, Dr. X. Zhang

Physical & Computational Science Directorate

Pacific Northwest National Laboratory

902 Battelle Blvd, Richland, Washington 99354, United States

E-mail: kevin.rosso@pnnl.gov (K.M.R.); xin.zhang@pnnl.gov (X.Z.)

[b] Dr. P. Chen, Prof. T. Chen

Key Laboratory of Nano-minerals and Pollution Control of Anhui Higher Education Institutes,

School of Resources and Environmental Engineering,

Hefei University of Technology

No.193 Tunxi Road, Hefei 230009, China

[c] Dr. T. Liu, Dr. D. Z. Wang, Prof. D. Wang

Department of Chemistry

Merkert Chemistry Center

Boston College

2609 Beacon Street, Chestnut Hill, Massachusetts 02467, United States

Methods

**Chemicals.**

Iron (III) chloride hexahydrate (FeCl_3_·6H_2_O, 97%) and methylene blue (C_16_H_18_ClN_3_S·3H_2_O, ≤100%) were purchased from Sigma Aldrich Chemical Reagent Co., Ltd. Sodium acetate (CH_3_COONa·3H_2_O, pure) was purchased from Amresco Chemical Reagent Co., Ltd. Ethanol (200 proof) was purchased from Decon Laboratories. Sodium hydroxide (NaOH, Sigma-Aldrich, ACS reagent, ≥ 97.0%, pellets). All chemicals are analytical purity and used directly without any further treatment. Deionized water (DI, pH 7.02, 18.2 MΩ·cm) used in this work was made with a Barnstead water purification system.

**Materials synthesis and characterizations.**

Three different hematite nanoplates were synthesized based on the method in ref ^[1]^:

**Synthesis of hexagonal-shaped hematite nanoplates.** 270.3 mg FeCl_3_·6H_2_O was dissolved in a mixed solution (10 mL, Vethanol/Vwater is 9:1) under magnetic stirring at room temperature to form a 0.1 M homogeneous solution. Then, 1.3276 g of sodium acetate was slowly introduced into the above suspension with stirring. After stirring at room temperature for about 6 h, the obtained mixture was transferred into a 20 mL Teflon liner stainless steel autoclave and held at 180 °C for 12 h.

**Synthesis of rhombic-shaped hematite nanoplates.** A total of 27.0 mg FeCl_3_·6H_2_O was dissolved into 10 mL DI water at room temperature to form a 10 mM homogeneous solution. Then, the obtained solution was transferred into a 20 mL Teflon liner stainless steel autoclave and heated at 120 °C for 3 d.

**Synthesis of hexagonal bipyramid-shaped hematite nanocrystals.** A total of 67.6 mg FeCl_3_·6H_2_O was dissolved into 10 mL DI water at room temperature to form a 25 mM homogeneous solution. Then, the obtained solution was transferred into a 20 mL Teflon liner stainless steel autoclave, put into an oven equipped with a rotation rack (10 rpm), and heated at 120 °C for 3 d.

The resulting precipitates were recovered by centrifugation at 13000 rpm and then washed several times with deionized water until the rinse water became colorless and its pH was around 7.53 The washed powders were dried in air at 80 °C overnight.

The phase and crystallinity of the as-synthesized samples were characterized by X-ray diffraction (XRD, PANalytical, Almelo, The Netherlands) equipped with Cu Kα radiation performed at 50 kV and 40 mA (2θ range from 5° to 75°). Particle morphologies were examined by scanning electron microscopy (SEM, FEI, Helios NanoLab 600i) (around 5 nm carbon thin film was coated on the samples to improve the imaging quality) and transmission electron microscopy (TEM, FEI Titan) (samples were prepared by drop-casting suspensions onto TEM copper grids, lacey carbon film, 300 mesh, Ted Pella, Inc.). The specific surface area from Brunauer-Emmett-Teller (SSA_BET_) of the two hematite samples was calculated from the nitrogen adsorption-desorption experiment that was conducted with a Micromeritics ASAP 2020 surface area analyzer.

**Photocatalytic degradation measurements and characterizations.**

The photocatalytic properties of the different hematite nanoplates were evaluated by examining their effectiveness for MB degradation in an aqueous solution under visible light illumination at room temperature. Circulating cooling water (16 ± 2 °C) was employed to stabilize the reaction temperature. The flow rate of the cooling water was 10 mL·min^-1^. Before illumination, 25.0 mg of the photocatalyst was dispersed in 50.0 mL of MB aqueous solution with an initial concentration of 10 mg·L^-1^ in a 200 mL cylindrical Pyrex vessel; 1.5 mL of hydrogen peroxide (30%, w/w) was added into the solution. The initial pH of the reaction systems was monitored or adjusted, and the pH values of all the solutions were measured using a Thermo Scientific instrument (110P-01A, America). After 2 h for the MB adsorption-desorption equilibrium condition, the suspension was illuminated by a 200 W xenon arc lamp (UXL-360, HI2608). At the termination of the light source emission, a 420 nm filter was added to limit the applied spectrum to that consistent with visible light. The light intensity at the center of the cylindrical Pyrex vessel was measured to be ∼14 mW·cm^-2^. A total of 1 mL of mixed solution was taken out from the vessel at predetermined time intervals of 1 h increments and centrifuged for 10 min at 13000 rpm to separate the solid and solution. The supernatant was transferred into a semi-micro-cuvette (Fisher Scientific) and measured at 664 nm on a UV-vis spectrophotometer (UV-vis 2501PC, Shimadzu) at a scan rate of 0.5 nm/min and a wavelength range of 800-200 nm.

**In-situ electron paramagnetic resonance (EPR) spectroscopy.**

The reactive oxygen species (ROS) produced in the photocatalytic processes were measured by a Bruker ELEXSYS E580 electron paramagnetic resonance spectrometer at 25 °C operated at the X-band with a microwave frequency of 9.32 GHz. Solutions were prepared from stock solutions and mixed immediately with spin trap 5-(diethylphosphono)-5-methyl-1-pyrroline N-oxide (DEPMPO) right before EPR experiments. A glass capillary (VitroTubes™, ID 0.8 mm and OD 1 mm) was used to hold the sample within a 4 mm EPR tube in EPR cavity, both ends of the capillary were sealed using Critoseal™ Leica Microsystems capillary tube sealant. Kinetic measurements were performed by recording EPR spectra continually before and after visible light irradiation with a sweep time of ~ 10.83 s at a microwave power of 20 mW. The sweep width is set to 150 G, the time constant is 40.96 ms, and the field modulation amplitude is 0.5 G. Absolute spin concentrations of the samples were determined by comparing to a calibration curve of double integration vs. concentration of TEMPO (2,2,6,6-tetramethyl-1-piperidinyloxyl) at varying concentrations (0.1 mM to 100 mM).

**Intensity modulated photocurrent spectroscopy (IMPS) measurements.**

IMPS spectra were recorded using a Solartron ModuLab XM potentiostat coupled with a Frequency Response Analyzer (FRA, Solartron ModuLab) and a 405 nm LED (ThorLab) with 1000 mA max power and controlled by the ModuLab XM DSSC software. IMPS data were measured using a 30% light intensity modulation (centered at ca. 95 mW/cm^2^) varying between 10 kHz and 0.01 Hz. A three-electrode configuration was utilized, with a Pt wire serving as the counter electrode, saturated calomel electrode as the reference electrode, and the fabricated electrode with hematite solution dropcasted on FTO glass substrate as the working electrode. The electrolyte was a 1 M NaOH solution.

**Electrode Fabrication.** A 2.2 mm thick glass slide coated with fluorine-doped tin oxide conductive film (Sigma-Aldrich, 7 Ω/sq surface resistivity) was cleaned in acetone, methanol, isopropanol and DI water. 2 mg hematite powders were mixed with 4 mL 0.3 wt% polyethylenimine (PEI) aqueous ethanol solution (1:1 v/v) to make a hematite solution. Hematite nanoparticles were dispersed in an ultrasonic bath for 30 min. Then 30 μL of the hematite solution was dropcasted on a 1 × 1 cm2 FTO glass substrate and dried naturally in ambient air at room temperature. The FTO glass substrate with monodispersed hematite was then annealed in air at 500 °C to remove organic solvents in the hematite solution and improve the adhesion between the hematite nanoparticles and the substrate. To make a photoelectrode, the resulting hematite sample was connected to a Cu wire using Ag paste (MG Chemicals, 8331 Silver Conductive Epoxy Adhesive) and protected with nonconductive epoxy (Loctite 615 Hysol Epoxy Adhesive).

**Photoelectrochemical (PEC) Characterization.** PEC characterization was carried out using a Solartron ModuLab XM potentiostat. A three-electrode configuration was employed, with hematite as the working electrode, a saturated calomel electrode (SCE, CH instruments, model number: CHI150) as the reference electrode and a Pt wire as the counter electrode. The electrolyte was a 1 M NaOH (Sigma-Aldrich, ACS reagent, ≥ 97.0%, pellets) solution. The light source for all data presented in this work was a 405 nm LED (ThorLab) (95 mW/cm^2^). In a typical J−V plot, the voltage was swept linearly from negative to positive at a rate of 20 mV s^−1^ with front illumination.

**Time-of-Fight Secondary Ion Mass Spectrometry (ToF-SIMS)**

ToF-SIMS measurement was performed at Environmental Molecular Sciences Laboratory (EMSL), which located at Pacific Northwest National Laboratory. A TOF.SIMS5 instrument (IONTOF GmbH, Münster, Germany) was used. A 25 keV pulsed Bi_3_^+^ beam was used as the analysis beam to collect SIMS spectra. The Bi_3_^+^ beam was focused to be ∼5 μm diameter and scanned over a 200 × 200 μm^2^ area. The current of the pulsed Bi_3_^+^ beam (10 kHz) was about 0.56 pA, and data collection time is ∼96 s per spectrum. Mass resolution was in a range of 5000-7000, varying from sample to sample due to sample roughness. A low energy (10 eV) electron flood gun was used for charge compensation in all measurement. The ToF-SIMS sample preparation was as follows: as-prepared samples were dispersed in water by a sonicator for 5 min, and then the dispersed suspension was dropped on a silicon wafer, which was then dried under ambient conditions prior to being introduced into the chamber.

**X-ray Photoelectron Spectroscopy (XPS)**

XPS measurements were performed with a Physical Electronics Quantera Hybrid Scanning X-ray Microprobe. This system uses a focused monochromatic Al Kα X-ray (1486.7 eV) source for excitation and a spherical section analyzer. The instrument has a 32-element multichannel detection system. The X-ray beam is incident normal to the sample and the photoelectron detector is at 45° off-normal. High energy resolution spectra were collected using a pass-energy of 69.0 eV with a step size of 0.125 eV. For the Ag 3d_5/2_ line, these conditions produced a FWHM of 0.92 eV ± 0.05 eV. The binding energy (BE) scale is calibrated using the Cu 2p_3/2_ feature at 932.62 ± 0.05 eV and Au 4f_7/2_ at 83.96 ± 0.05 eV.

**Simulation methods.**

All calculations were performed applying the pseudopotential plane-wave DFT approach implemented in the NWPW module of the NWChem computational chemistry package ^[2, 3]^. The Perdue-Burke-Ernzerhof exchange-correlation functional (PBE96) was employed throughout the PSPW optimization procedure ^[4]^. The DFT + U approach according to Dudarev et al. was applied to account for the electron correlation in the localized Fe 3d orbitals ^[5]^. Based on previous studies on goethite ^[6, 7]^, the effective on-site Coulomb and exchange interaction parameters for each Fe atom were set to 4 eV and 1 eV, respectively. Default pseudopotentials contained in NWChem were used, viz., the valence electron interactions with the atomic core were approximated with generalized norm-conserving Hamann ^[8]^ for O and H and norm-conserving Troullier-Martins ^[9]^ pseudopotentials for Fe, which contains 4s, 4p and 3d projectors and a semi-core correction. All the pseudopotentials were modified to the separable form suggested by Kleinman and Bylander ^[10]^.

Unrestricted calculations were performed because this is a spin-ordered system. To define the antiferromagnetic structure of hematite, spin penalty functions were used; details on this approach are available in Bylaska et al. ^[11]^ Briefly, spin penalty functions were introduced in the pseudopotential by adding a scaling factor to the nonlocal pseudopotential for up/down spins on specified atoms and angular momentum to stabilize the electronic wavefunction at a selected set of sites^[2, 3]^. This breaks the symmetry of the wavefunction creating spin localization, which makes it easier to define antiferromagnetic structures. With spin penalty functions, the pseudopotential is modified to

$E_{psp-penalty}=\sum_{\sigma=\uparrow,\downarrow} \sum_{i=1}^{n_{elc}^{\sigma}} \sum_{I=1}^{n_{ions}} \left( \left\langle\psi_{i}^{\sigma} | V_{local}^{I} | \psi_{i}^{\sigma} \right\rangle+\sum_{l=0}^{l_{max}^{I}} \sum_{m=-l}^{l} \sum_{n=1}^{n_{max}^{I}} \sum_{n^{'}=1}^{n_{max}^{I}} \left( 1-\delta_{l,l_{I}^{\sigma}}\delta_{I,ionlist}\left( \xi_{I,l}^{\sigma}-1 \right) \right)\times\left\langle\psi_{i}^{\sigma} | P_{nlm}^{I} \right\rangle h_{l,n,n^{'}}^{I}\left\langle P_{n^{'}lm}^{I} | \psi_{i}^{\sigma} \right\rangle\right)$ (1)

where ξ_I,l_^σ^ and *l*_I_^σ^ specify the strength and locations of the penalties, respectively.

Periodic boundary conditions were used and wavefunction solutions were obtained at the Γ-point with a wavefunction cutoff energy of 100 Ry and a density cutoff energy of 200 Ry. All atomic positions were entirely relaxed in a fixed unit cell using the default NWChem DRIVER optimizer until the forces on the atoms were converged to 10^-2^ eV/Å and the total energy was converged to 10^-5^ eV. The simulations were performed by using the hexagonal hematite unit cell (a = b = 5.090 Å, c = 13.892 Å, α = β = 90.0°, γ = 120.0°). We assume all surfaces are hydroxylated O-terminations in contact with the solution. The (001) surface slab was expanded by doubling the conventional unit cell along a and b directions to produce a (2×2) surface slab (a = b = 10.196 Å) with 8 Fe layers. To create the (104) surface, we used Euler transformation to get the new unit cell whose lattice parameters are a = 9.050 Å, b = 5.090 Å, c = 5.492 Å, α = 90.0°, β = 94.9°, γ = 124.2° and expanded the cell in a and b directions to obtain a (1×2) slab (a = 9.050 Å, b = 10.180 Å) with 8 Fe layers. The first three layers are oxygen (hydroxyl) ions, followed by two iron layers below. This structure can be represented by O(H)-O(H)-O(H)-Fe-Fe-R. The (116) surface was created by expanding the new unit cell along direction with lattice parameters a = 7.483 Å, b = 9.050 Å, c = 10.986 Å, α = 110.2°, β = 95.8°, γ = 63.6° after Euler transformation. We use a (1×1) surface slab with 12 Fe layers and the first three oxygen (hydroxyl) layers are followed by one layer of oxygen ions and the next two layers are iron layer and oxygen layer, which can be represented as O(H)-O(H)-O(H)-Fe-O(H)-Fe-O(H)-R. In our notation, for (001), (104) and (116) we only specify the top three, five and seven atomic layers that identify the surface termination, where R represents the remaining atomic layers that retain the bulk unit cell stacking sequence. All the slabs consist of an upper and a lower surface with the same outmost atomic layer. Both (001) and (104) surface slabs contained 32 Fe, 60 O and 24 H atoms while (116) slab consisted of 24 Fe, 46 O and 20 H atoms and the three surfaces were modeled by fully relaxed slabs without inversion symmetry and a vacuum of approximately 10 Å in between with the thickness of slab 9.6 Å, 13.9 Å and 11.3 Å (distance in c direction between two oxygen atoms on surface) respectively. For the simulations on the reactions in the presence of light, the total system of (001), (104) and (116) surface contained 655 (328 spin up electrons and 327 spin down electrons), 655 (328 spin up electrons and 327 spin down electrons) and 503 (252 spin up electrons and 251 spin down electrons) valence electrons, respectively. For the simulations on the reactions in the absence of light, the total system of (001), (104) and (116) surface contained 654 (327 spin up electrons and 327 spin down electrons), 654 (327 spin up electrons and 327 spin down electrons) and 502 (251 spin up electrons and 251 spin down electrons) valence electrons, respectively.

The nudged elastic band (NEB) ^[12-17]^ method was used to locate a reaction path (RP) connecting two local energy minima representing the reactant and product states on the potential energy surface (PES). An elastic band with N images can be denoted by $\left[ R_{1}, R_{2}, R_{3},\ldots, R_{N} \right]$. The initial (R_1_) and final (R_N_) state structures were fully relaxed before NEB calculations. In between the initial and final configurations, $N-2$ intermediate images were generated on a discrete representation of path. In our calculations, 9 intermediate images (N = 11) were used. An initial path was first generated using a linear interpolation scheme and then an iterative algorithm was used to shift the intermediate images to the RP. The $N-2$ intermediate images were adjusted using an effective NEB force acting on the atoms in combination with a gradient-based optimization algorithm, while the two endpoint configurations were kept fixed. The total force acting on an image is the sum of the spring force along the local tangent and the true force perpendicular to the local tangent**.**

$F_{i}=F_{i}^{s}|_{\parallel}-\nabla E\left( \mathbf{R}_{i} \right)|_{\perp}$ (2)

The spring force is given by

$F_{i}^{s}|_{\parallel}=k\left( \left| \mathbf{R}_{i+1}-\mathbf{R}_{i} \right|-\left| \mathbf{R}_{i}-\mathbf{R}_{i-1} \right| \right)\hat{\tau}_{i}$ (3)

where *k* is the spring constant, and $\hat{\tau}_{i}$ is the normalized local tangent at image i. The true force is

$\nabla E\left( \mathbf{R}_{i} \right)|_{\perp}=\nabla E\left( \mathbf{R}_{i} \right)-\nabla E\left( \mathbf{R}_{i} \right)\cdot\hat{\tau}_{i}$ (4)

where E is the energy of the system, which is a function of all the atomic coordinates. An optimization algorithm was then used to move the images according to the force in Eq. (2). A projected velocity Verlet algorithm^17^ was applied. The highest energy point along a RP then gives an estimate for the activation energy for the transition.


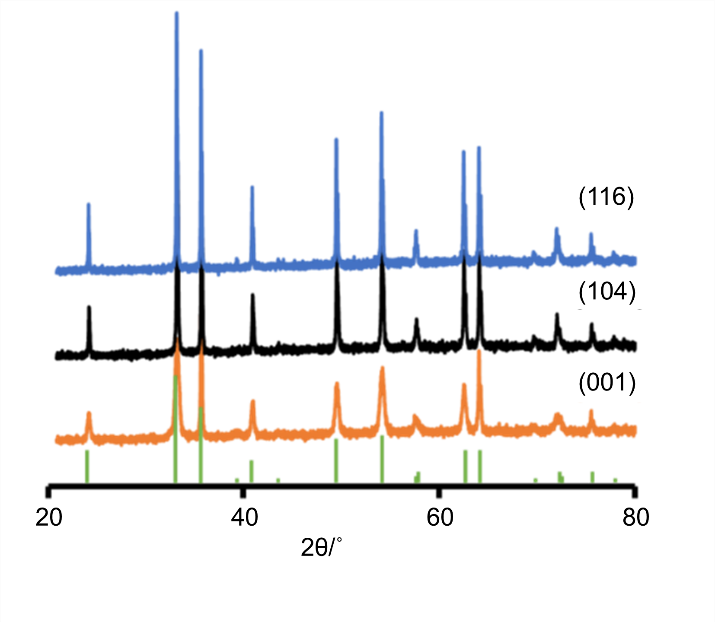


**Figure S1.** XRD patterns of three different hematite nanoparticles (Green pattern: α-Fe_2_O_3_ PDF#33-0664)

**Table S1.** summary of specific surface area (SSA), bandgap, and adsorption site of three different hematite nanoparticles

| **Hematite** | **SSA (m^2^/g)^1^** | **Bandgap (eV)^2^** | **Surface active site (site/g)** |
| --- | --- | --- | --- |
| (001) | 25.5/25.4 | 2.2/2.1 | 113*10^18 |
| (104) | 8.9/14.3 | 2.1/2.0 | 37*10^18 |
| (116) | 0.2/2.9 | --/1.8 | 5*10^18 |

Note: ^1^ SSA was obtained from BET test (first value) or theory calculation (second value), respectively. ^2^ Bandgap was obtained from UV-vis test (former value) or DFT calculation (later value), respectively. --meant this value couldn’t be detested from UV-vis test.

**Table S2.** pH values for the MB adsorption isotherms by three different hematite nanoparticles

|  |  |  | (001) | (104) | (116) |
| --- | --- | --- | --- | --- | --- |
| methylene blue | | before | 5.49-5.59 | 5.46-5.62 | 5.43-5.66 |
|  |  | after | 5.40-5.46 | 5.12-5.25 | 5.30-5.50 |

**Experimental data fitting models**

**Models of equilibrium adsorption isotherm**: Langmuir, Freundlich, and Temkin isotherm models.

Langmuir isotherm model assumes that each active site can only hold one adsorbate molecule. The nonlinear Langmuir isotherm equation is expressed as,

*q_e_ = q_max_*$\frac{K_{L}C_{e}}{1+K_{L}C_{e}}$ （5）

where q_e_ (mg/m^2^) and C_e_ (mg/L) are the amounts of adsorbed MB onto hematite nanoparticles and MB concentration at equilibrium, respectively, q_max_ (mg/m^2^) is the maximum amount of MB adsorbed per unit surface area of hematite nanoparticles, and K_L_ is the Langmuir adsorption constant.

Freundlich isotherm model is given by:

*q_e_ = K_F_*$C_{e}^{1/n}$ （6）

Where K_F_ and n are the two Freundlich adsorption constants representing the adsorption capacity and affinity, respectively.

Temkin isotherm model is given by:

*q_e_ = B ln(A*C_e_)*  （7）

Where B = RT/b, b (kJ/mol) is the Temkin constant related to adsorption heat, T (K) is the absolute temperature, R is the gas constant. A (L/g) is the Temkin isotherm constant.

**Models of adsorption kinetics**: The pseudo-first-order and pseudo-second-order model

The pseudo-first-order model can be expressed as,

$\frac{{dq}_{t}}{dt}=k_{1}\left( q_{e}-q_{t} \right)$ （8）

where q_e_ and q_t_ (mg/m^2^) are the amounts of adsorbed MB at equilibrium and at time t, respectively, k_1_ (min^-1^) is pseudo-first-order rate constant, and t (min) is contact time.

The pseudo-second-order relation is given as:

$\frac{{dq}_{t}}{dt}=k_{2}(q_{e}-q_{t})^{2}$ （9）

where k_2_ (g/mg/min) is the adsorption rate constant.

**Degradation model:** Langmuir-Hinshelwood pseudo-first-order model

-ln(C/C_0_) = K_3_·t + c （10）

Where K_3_ (h^-1^) is the pseudo-first-order degradation rate constant, and t (h) is the specific time. The kinetics were measured by plotting the -ln(C/C_0_) versus t. c is a constant


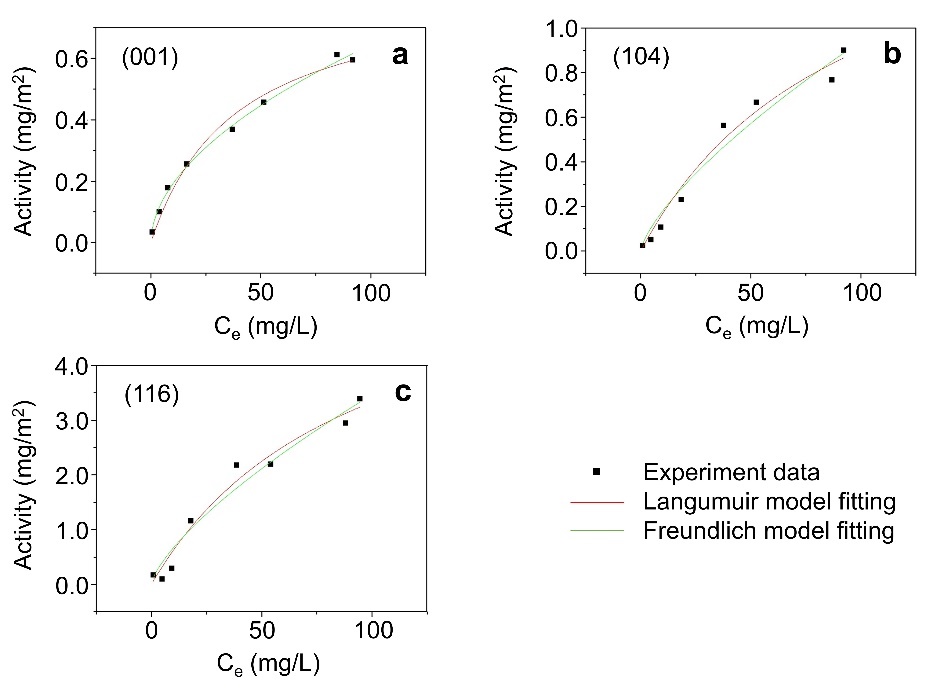


**Figure S2.** Adsorption isotherms of MB on three different hematite nanoparticles and their model fittings

**Table S3.** MB adsorption isotherms model parameters on three different hematite nanoparticles

|  | Langmuir model | | | Freundlich model | | | Temkin model | | |
| --- | --- | --- | --- | --- | --- | --- | --- | --- | --- |
|  | q_max_ | K_L_ | R^2^ | K_F_ | n | R^2^ | A | b | R^2^ |
| (001) | 0.84 | 0.026 | 0.978 | 0.057 | 1.896 | 0.995 | 0.972 | 20.89 | 0.875 |
| (104) | 1.44 | 0.014 | 0.973 | 0.040 | 1.492 | 0.945 | 0.476 | 13.28 | 0.816 |
| (116) | 6.20 | 0.012 | 0.972 | 0.138 | 1.430 | 0.960 | 0.511 | 35.05 | 0.786 |

**Table S3** indicated both Langmuir and Freundlich models can well fit the adsorption isotherms with R^2^ over 0.94 than Temkin model. Specially, (001) got a higher R^2^ value from Freundlich than Langmuir model, which is consistent with previous study ^[18]^, while higher R^2^ values for (104) and (116) in the Langmuir model. However, it is worth noting that the Langmuir model can successfully fit the adsorption data of all three sets of hematite, with R^2^ values exceeding 0.97. Hence, the maximum adsorption capacities (q_max_) follow the order of (001) < (104) ≤ (116). The (116) exhibits the highest q_max_ value of 6.20 mg/m^2^, which is significantly larger than that of (104) and (001), which are 1.44 mg/m^2^ and 0.84 mg/m^2^, respectively. Also, Langmuir adsorption model implies that the main adsorption occurred on the monolayer of the hematite surface.


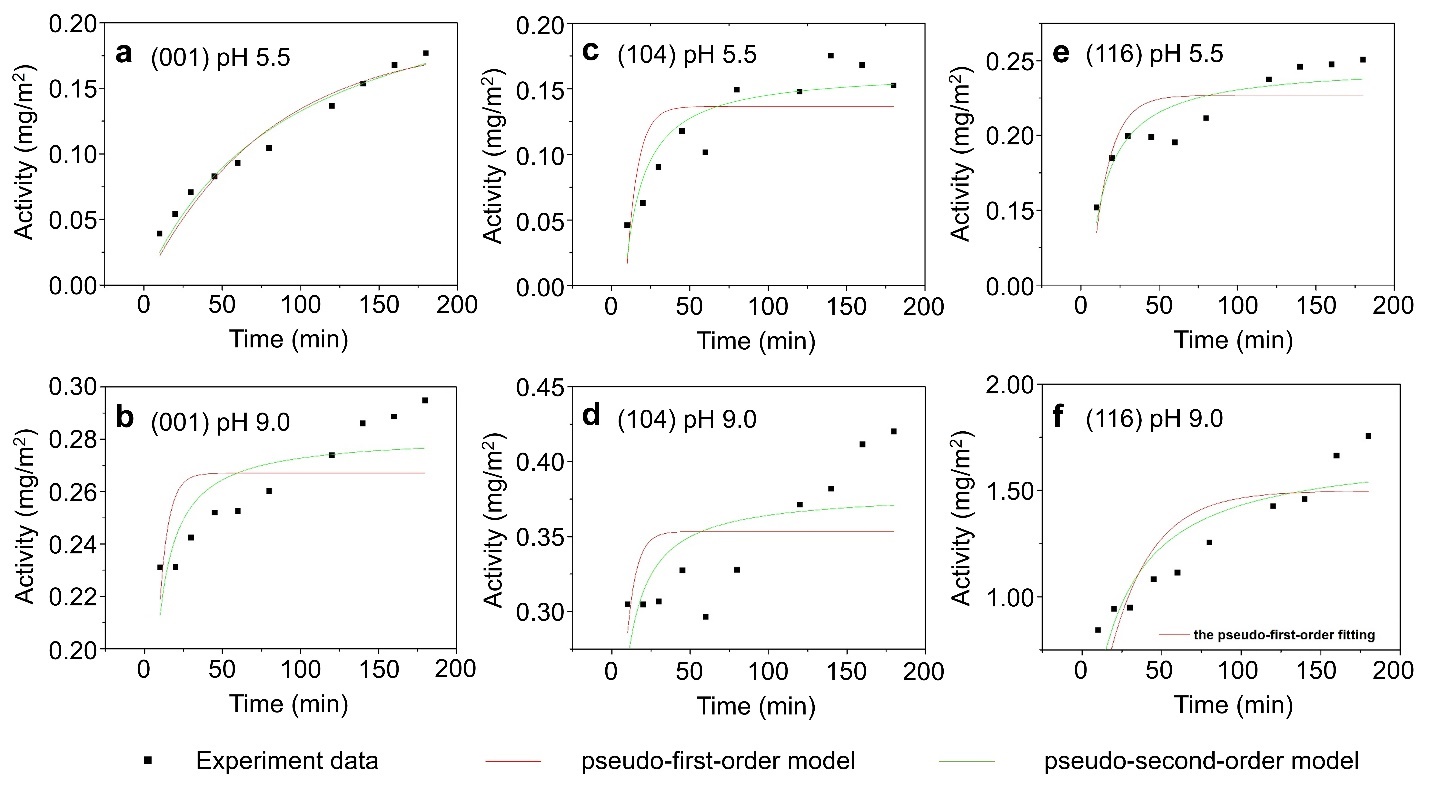


**Figure S3.** Adsorption kinetics of MB on three different hematite nanoparticles and their kinetic fittings

**Table S4.** MB adsorption kinetics model parameters on three different hematite nanoparticles

|  |  | pseudo-first-order model | | | pseudo-second-order model | | |
| --- | --- | --- | --- | --- | --- | --- | --- |
|  |  | q_e,f_ | k_1_ | R^2^ | q_e,s_ | k_2_ | R^2^ |
| 001 | pH 5.5 | 0.187 | 0.013 | 0.945 | 0.257 | 0.043 | 0.960 |
|  | pH 9.0 | 0.267 | 0.171 | 0.200 | 0.282 | 1.099 | 0.629 |
| 104 | pH 5.5 | 0.097 | 0.140 | 0.443 | 0.103 | 2.524 | 0.802 |
|  | pH 9.0 | 0.353 | 0.166 | 0.026 | 0.380 | 0.618 | 0.351 |
| 116 | pH 5.5 | 0.227 | 0.091 | 0.567 | 0.248 | 0.540 | 0.815 |
|  | pH 9.0 | 1.498 | 0.038 | 0.541 | 1.703 | 0.031 | 0.738 |


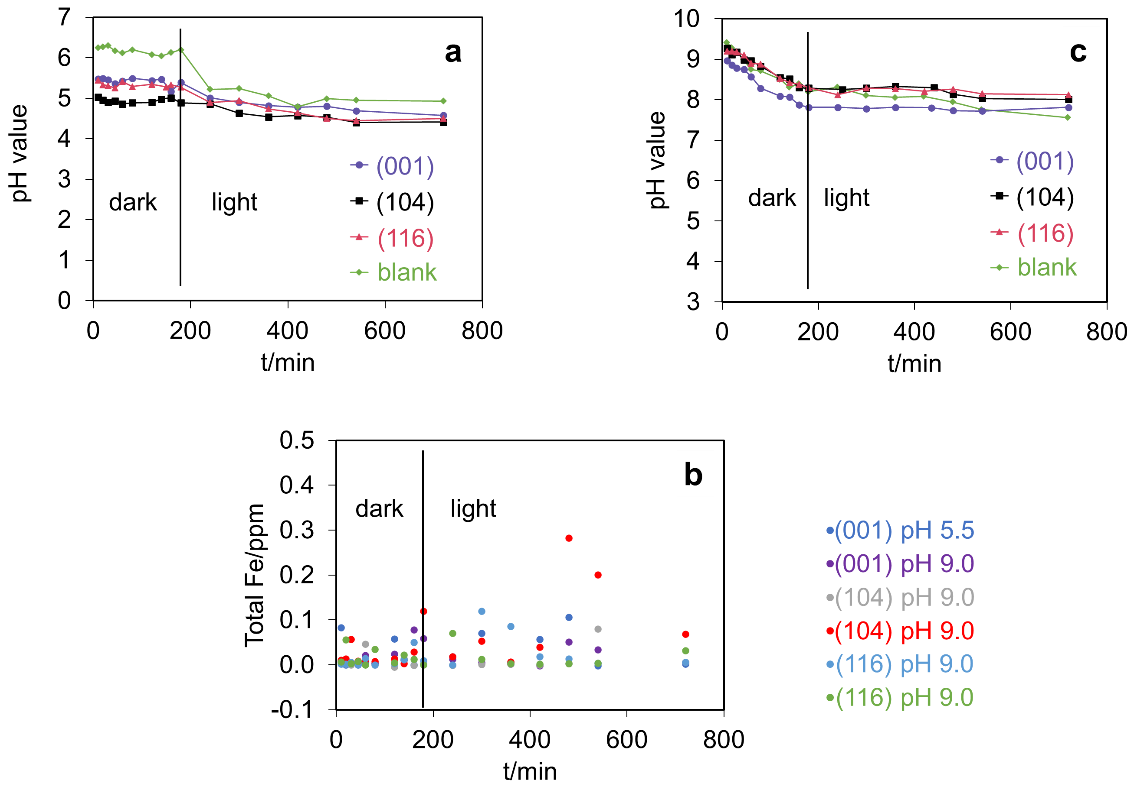


**Figure S4.** Real time monitoring of pH value and total Fe concentration tested by ICP-MS from the photoreactions.


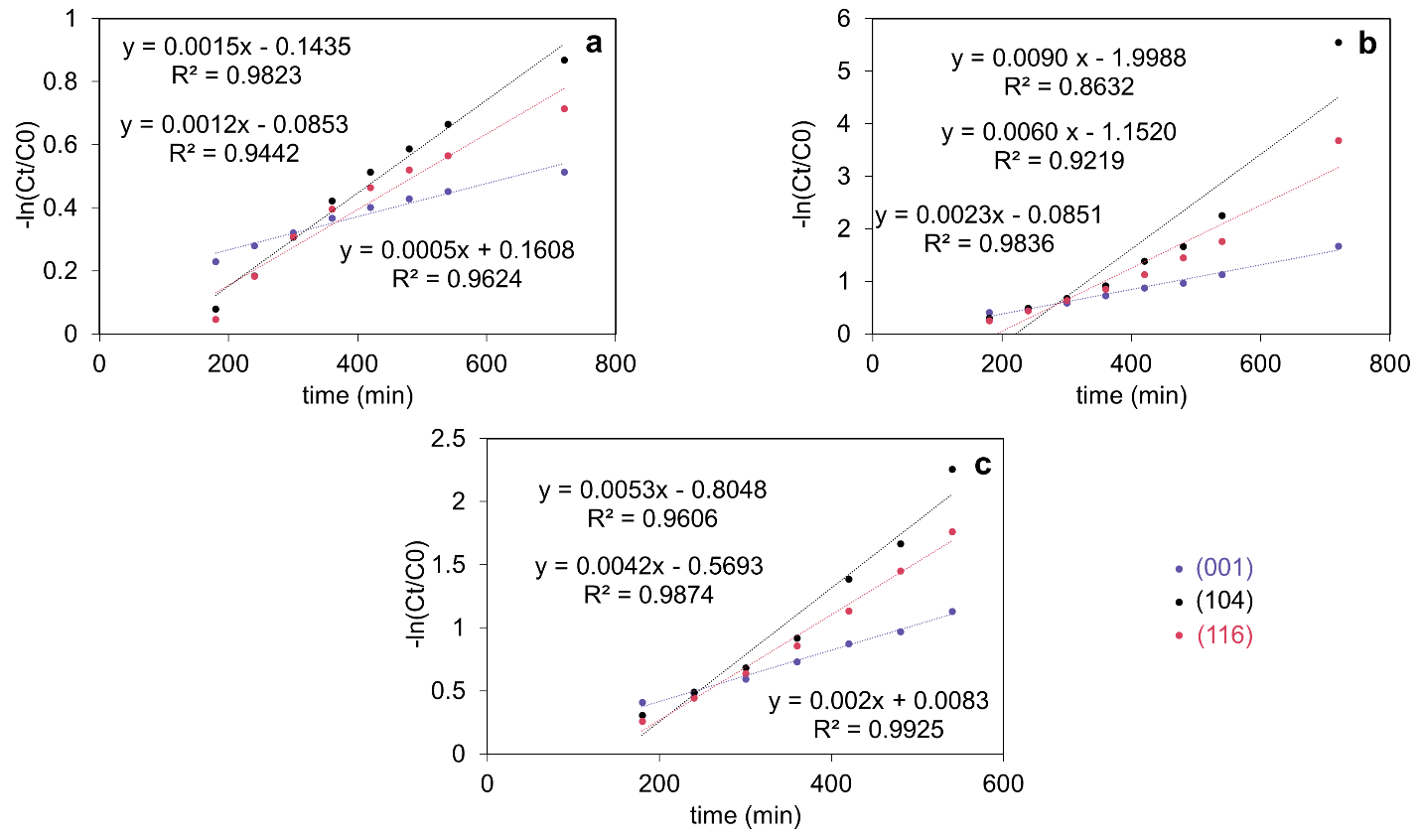


**Fig. S5.** Langmuir-Hinshelwood pseudo-first-order MB photodegradation kinetic fittings (a, pH 5.5. b, pH 9.0. c, a selected period from b).

**Table S5.** MB photodegradation kinetics fitting parameters on three different hematite nanoparticles

|  |  | L-H pseudo-first-order model | |
| --- | --- | --- | --- |
|  |  | k_3_ | R^2^ |
| 001 | pH 5.5 | 0.0005 | 0.9624 |
|  | pH 9.0 | 0.0020 | 0.9925 |
| 104 | pH 5.5 | 0.0015 | 0.9823 |
|  | pH 9.0 | 0.0053 | 0.9606 |
| 116 | pH 5.5 | 0.0012 | 0.9442 |
|  | pH 9.0 | 0.0042 | 0.9874 |


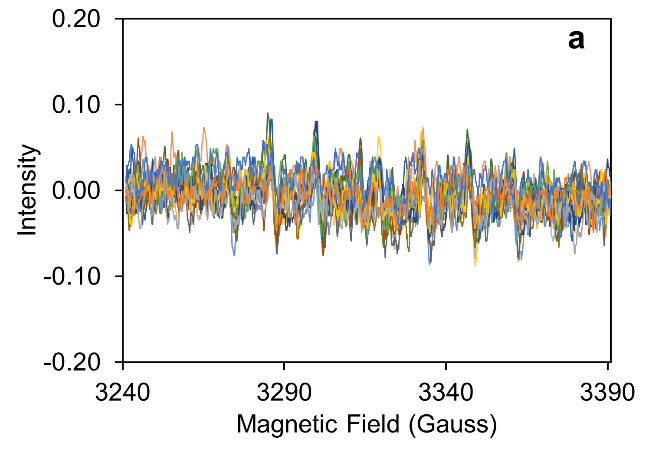

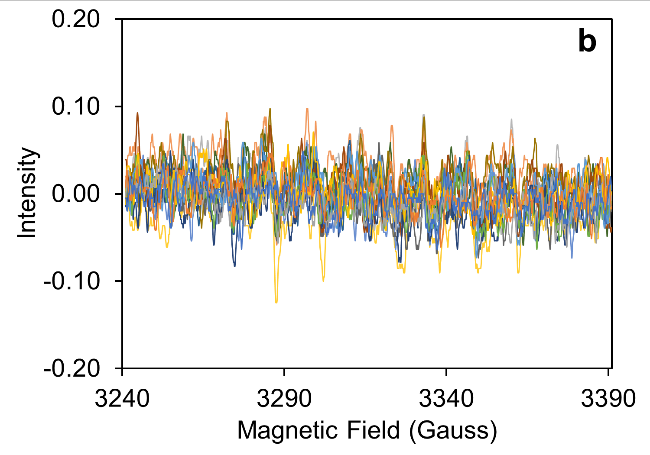


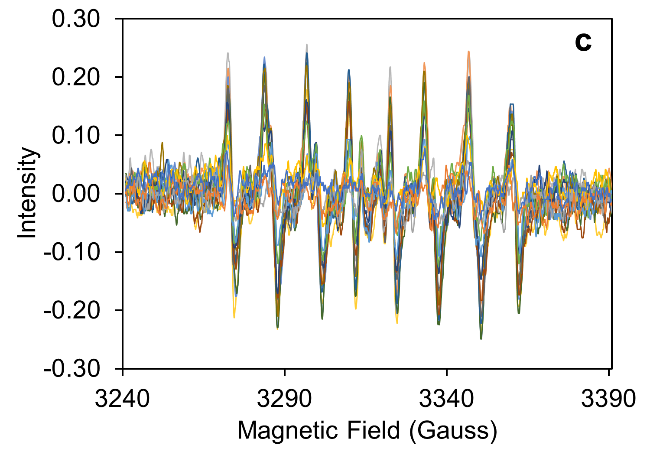

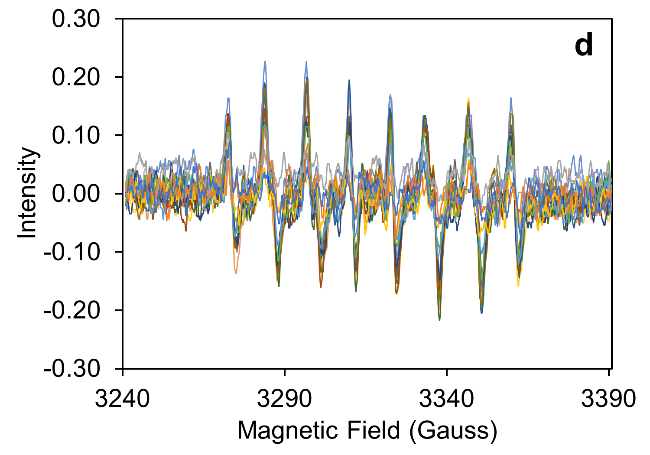


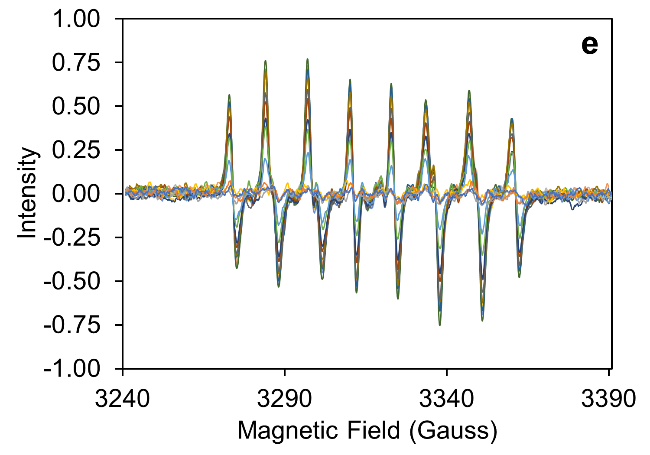

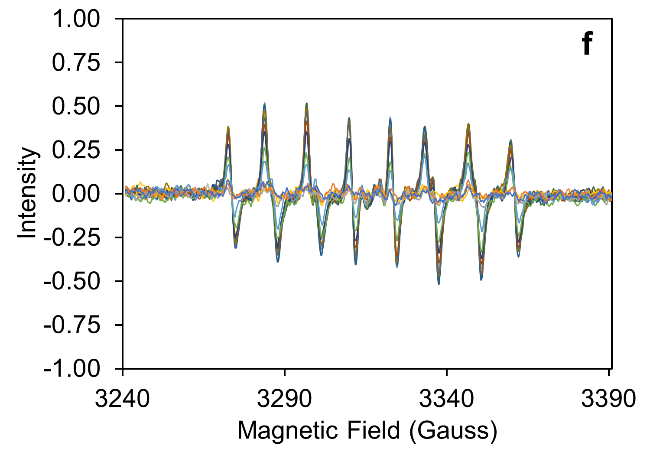


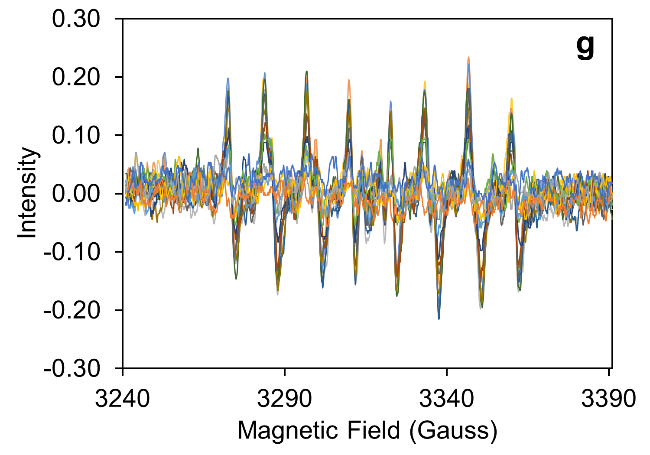

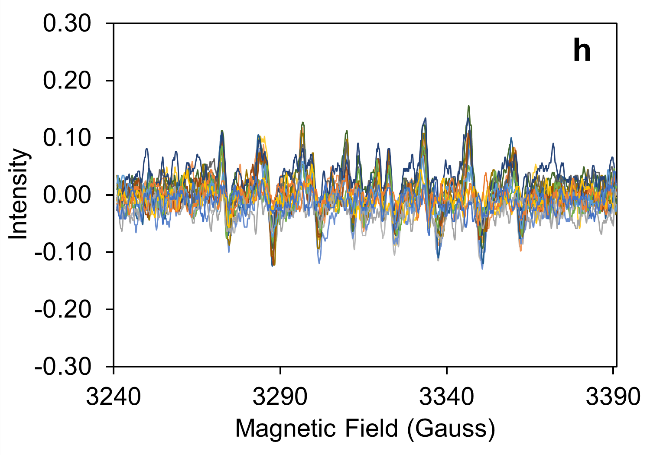


**
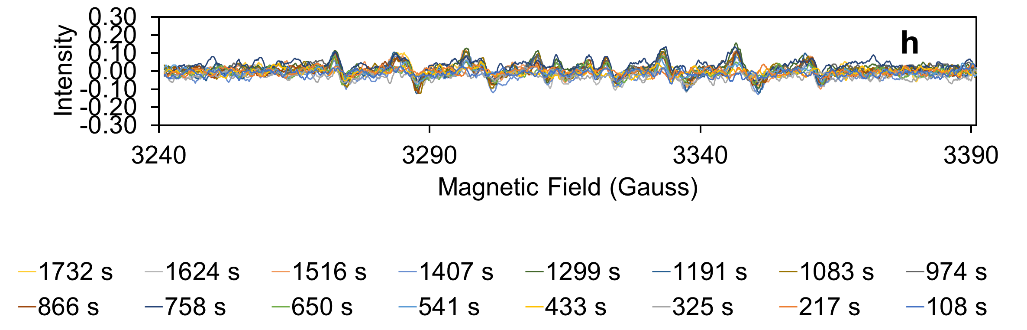
**

**Figure S6.** Measured DEPMPO-trapped EPR signal changes in aqueous solutions as a function of time in the presence of MB, hematite, and H_2_O_2_ at pH~5.5. (a) only H_2_O_2_, (b) H_2_O_2_ + MB, (c) H_2_O_2_ + (001), (d) H_2_O_2_ + (001) + MB, (e) H_2_O_2_ + (104), (f) H_2_O_2_ + (104) + MB, (g) H_2_O_2_ + (116), (h) H_2_O_2_ + (116) + MB.


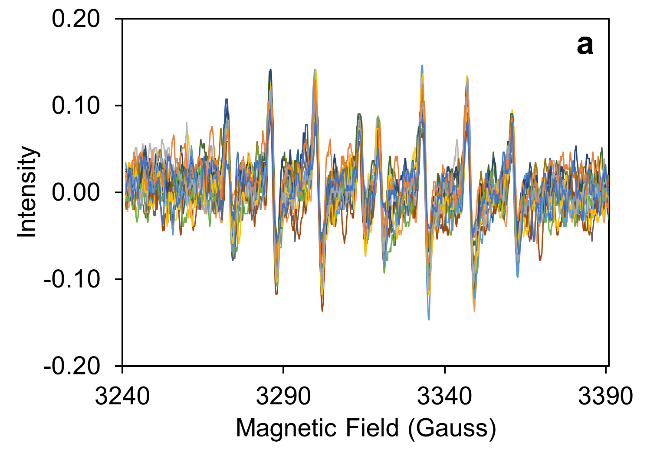

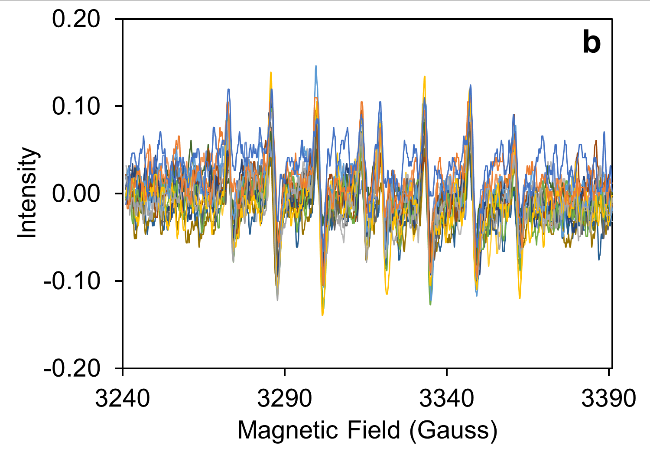


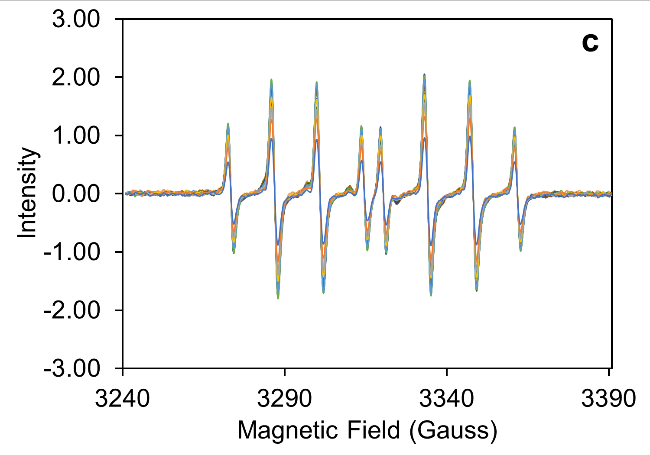

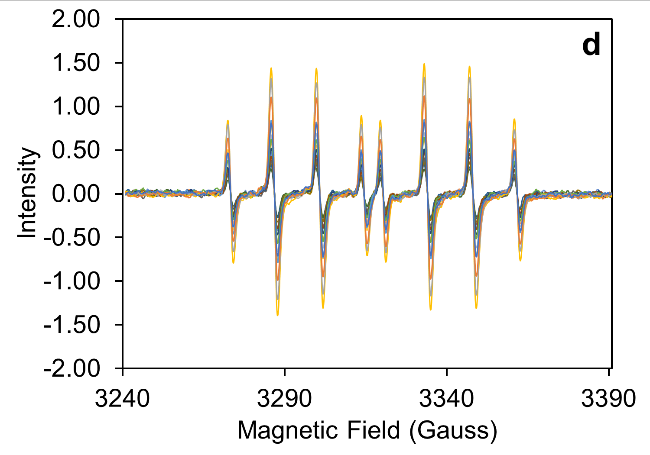


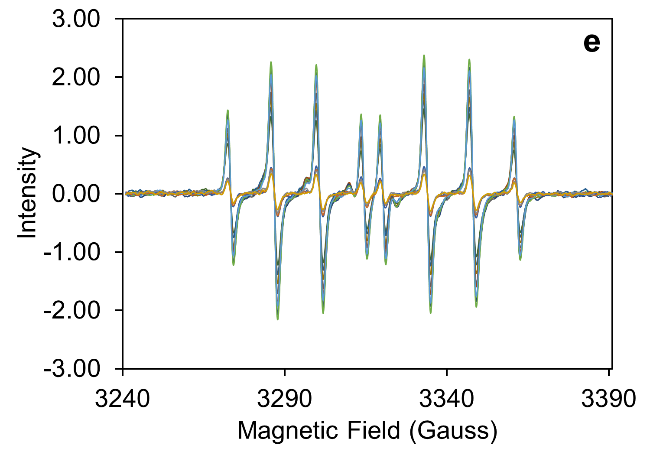

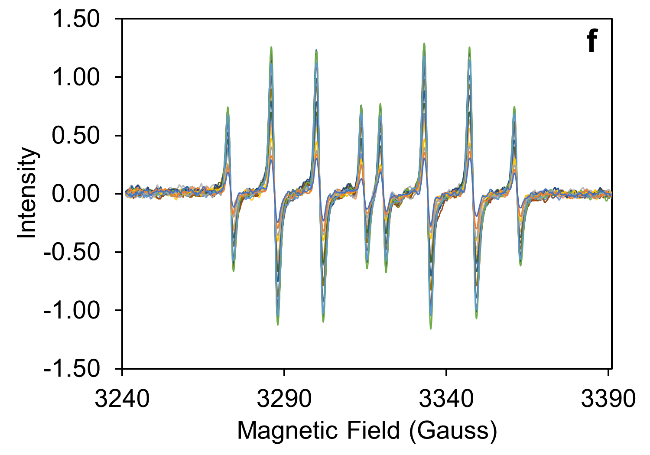


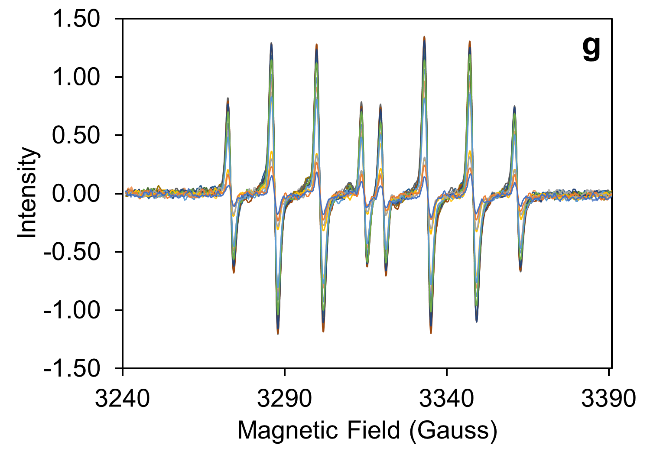

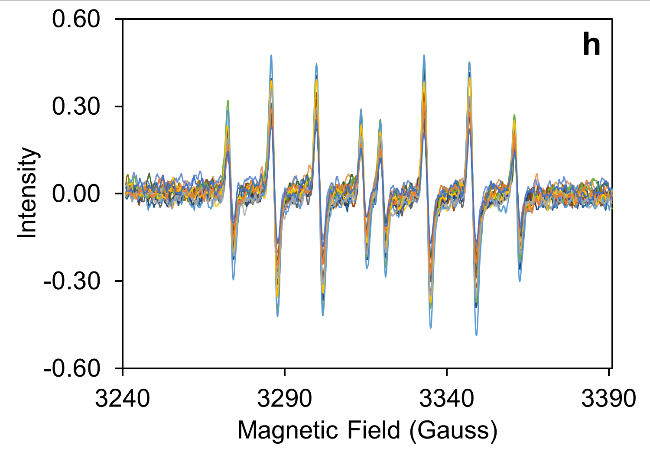


**
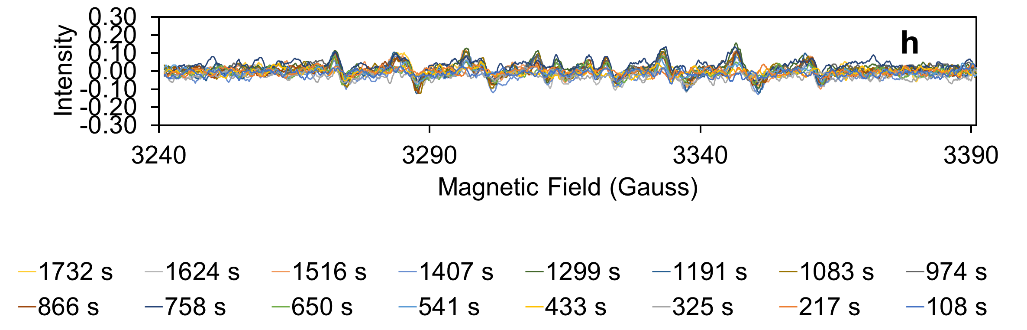
**

**Figure S7.** Measured DEPMPO-trapped EPR signal changes in aqueous solutions as a function of time in the presence of MB, hematite, and H_2_O_2_ at pH~9.0. (a) only H_2_O_2_, (b) H_2_O_2_ + MB, (c) H_2_O_2_ + (001), (d) H_2_O_2_ + (001) + MB, (e) H_2_O_2_ + (104), (f) H_2_O_2_ + (104) + MB, (g) H_2_O_2_ + (116), (h) H_2_O_2_ + (116) + MB.


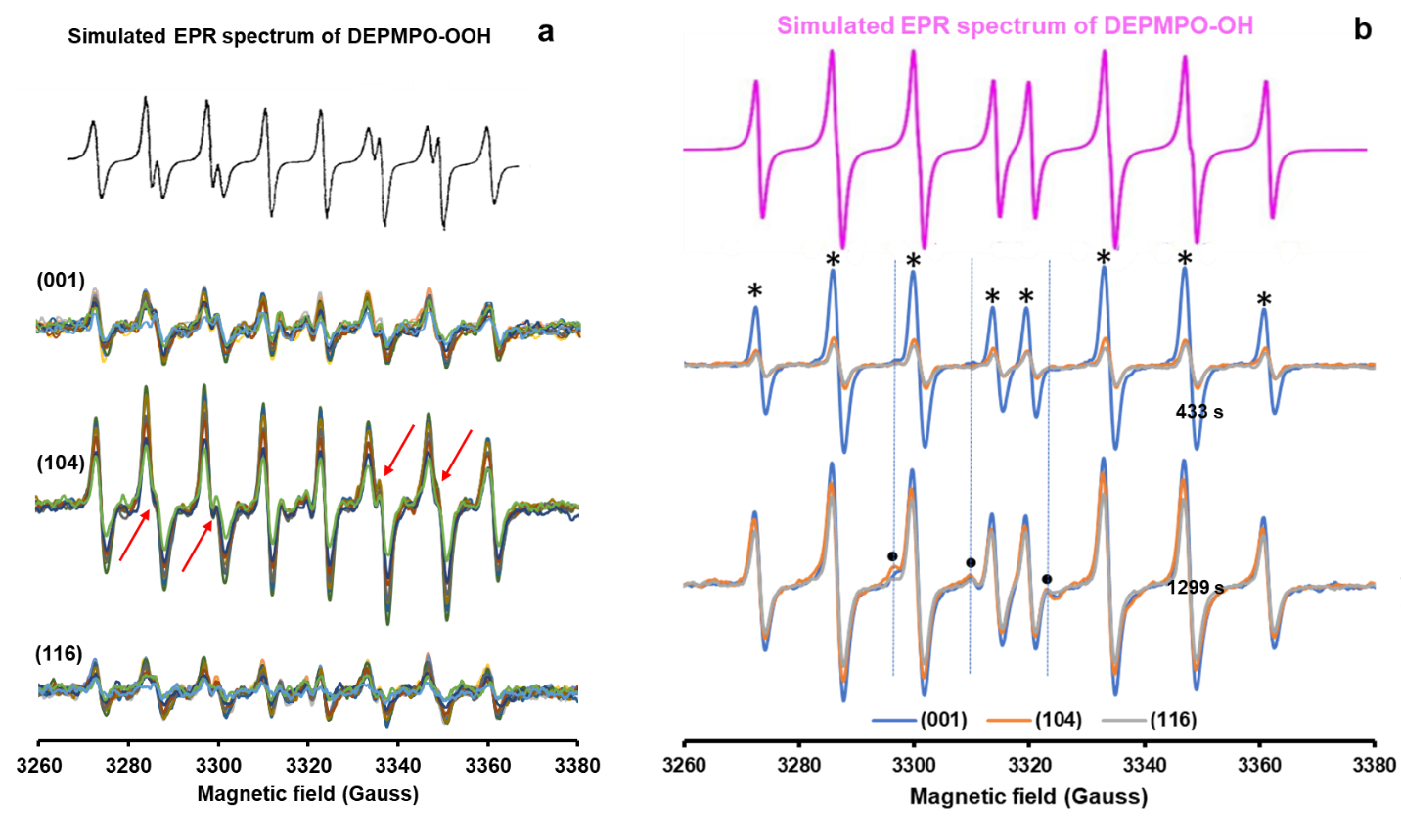


**Figure S8.** Measured DEPMPO-trapped EPR signal changes in aqueous solutions of hematite and H_2_O_2_. a. pH~5.5, hematite and H_2_O_2_ systems at 541s, 650s, 758s, 866s, 974s, 1083s, 1191s, 1299s, 1407s, 1516s, 1624s, and 1732s. original data can be found at Fig. S6c, e, g; b. pH~9.0, hematite and H_2_O_2_ systems at 433s and 1299s, original data can be found at Fig. S7c, e, g; Simulated EPR spectrum of DEPMPO-OOH ^[19]^, assuming an exchange between two conformers A and B of DEPMPO-OOH using parameters: A (50%), *a*_N_ = 13.2 G, *a*_H_^β^ = 11.9 G, *a*_P_ = 52.5 G, *a*_H_^γ^ = 0.7 G (1H), *a*_H_^γ^ = 0.43 G (6H), LW = 0.45 G; B (50%), *a*_N_ = 13.4 G, *a*_H_^β^ = 10.3 G, *a*_P_ = 48.5 G, *a*_H_^γ^ = 0.8 G (1H), *a*_H_^γ^ = 0.43 G (6H), LW = 0.45 G; *k*_exchange_ = 4*10^7^ s^-1^. Simulated EPR spectrum of DEPMPO-OH 3, using parameters *a*_P_ = 47.0 G, *a*_N_ =135 14.0 G, *a*_H_ = 13.0 G. * represents for DEPMPO-OH while ● represents for DEPMPO-OOH


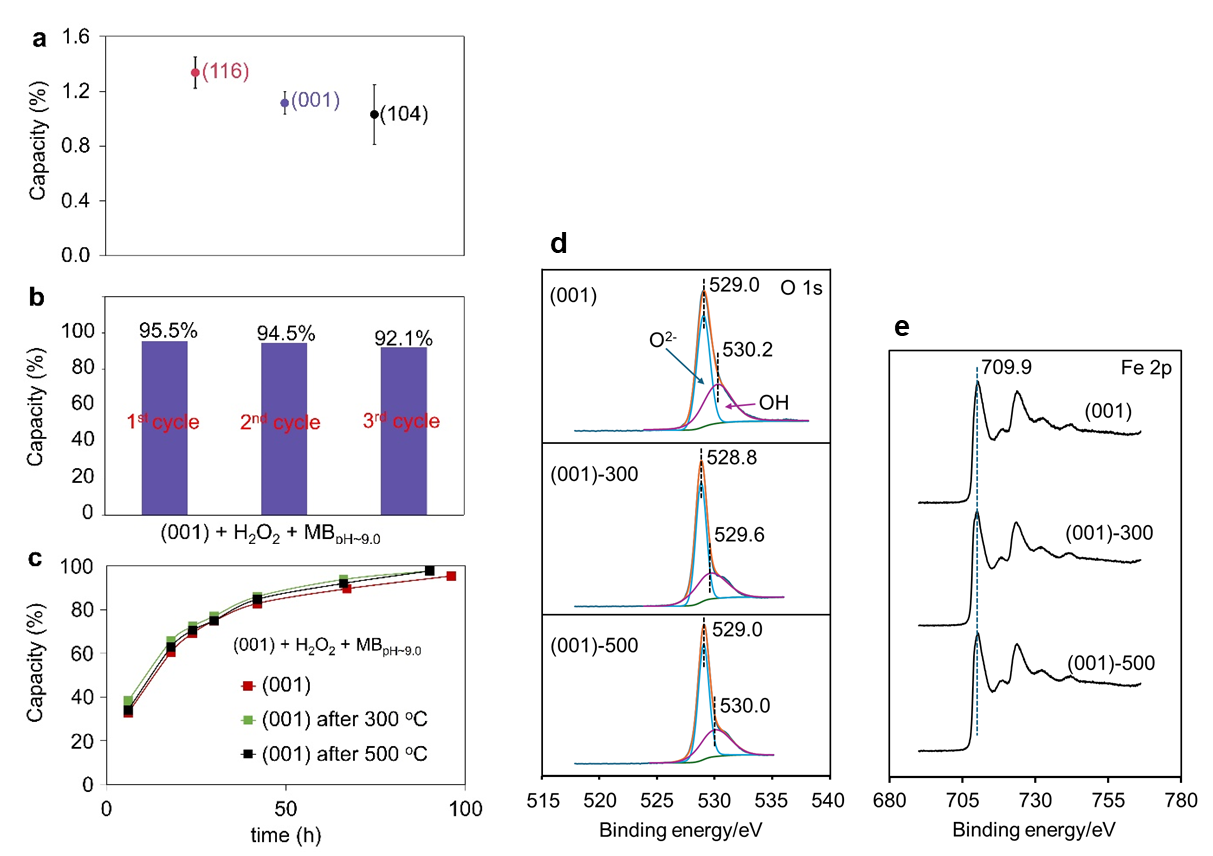


**Figure S9.** a, MB photodegradation capacity at 1299 s based on EPR experimental condition of Fig. 3a. b, MB removal capacity by recycling (001) three times at pH ~ 9.0, darkness. c, MB removal capacity after heat treatment of (001). d, O 1s XPS spectra of the (001) facet before and after heat treatment at 300 °C and 500 °C. e, Fe 2p XPS spectra of the (001) facet before and after heat treatment at 300 °C and 500 °C.

To examine the possible involvement of oxygen vacancies and surface Fe(II) in the dark reaction performance, XPS analyses of O 1s and Fe 2p regions were conducted for the pristine and annealed (001) hematite samples (**Fig. S9d, e**). The O 1s spectrum of the pristine sample exhibits a dominant sharp peak centered at ~529.0 eV, corresponding to lattice oxygen (O^2-^), and a minor broad peak at ~530.2 eV, attributed to surface hydroxyl groups. Notably, no shoulder or additional component associated with oxygen vacancy species was observed. After annealing at 300 °C and 500 °C, the O 1s spectra remain largely unchanged in both binding energy and intensity ratio. Similarly, the Fe 2p spectra show no discernible shifts or peak broadening upon annealing, and all Fe 2p spectra consistently indicate the presence of Fe(III) species, without any detectable signatures of Fe(II). These observations indicate that significant oxygen vacancies or Fe(II) species were neither present in the original sample nor introduced during the heat treatment process.


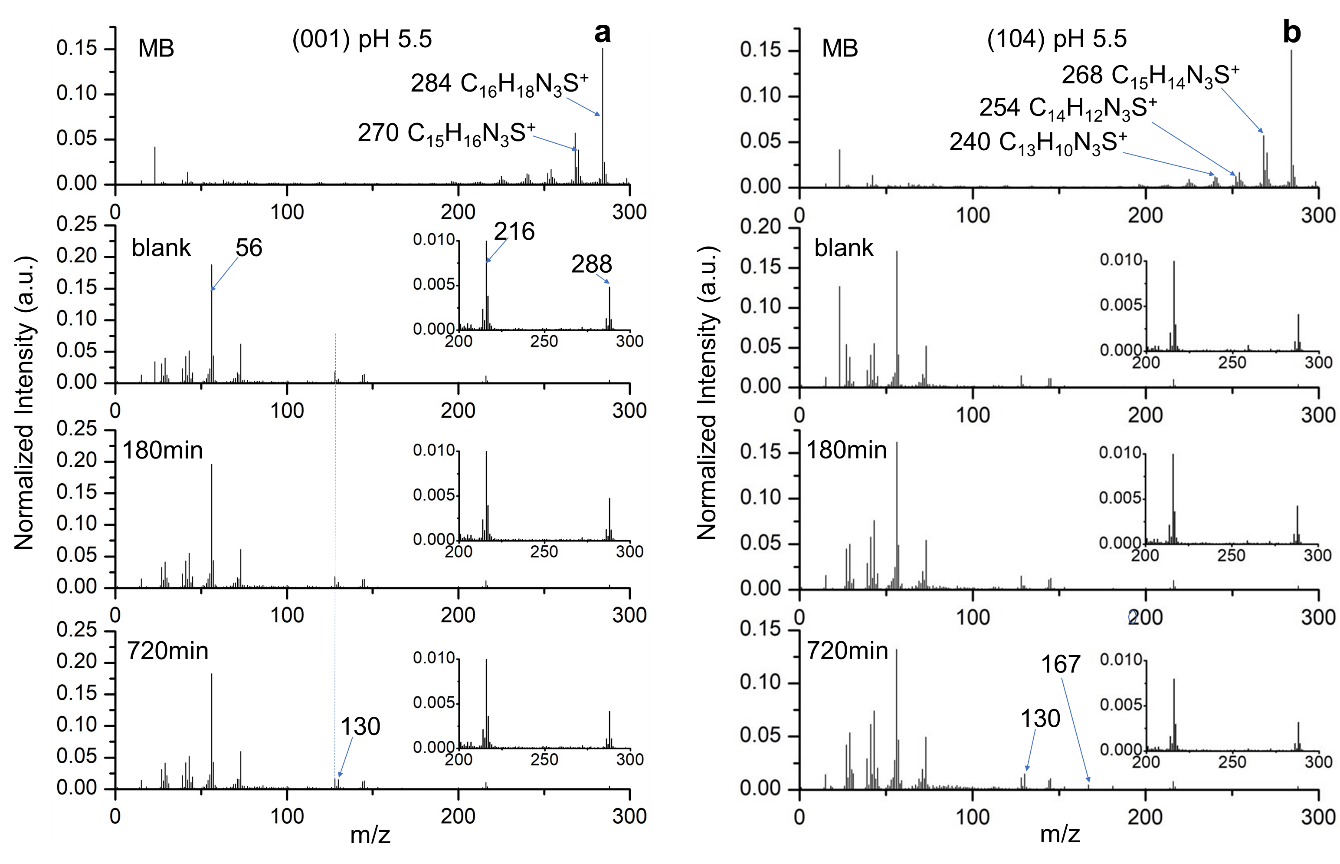


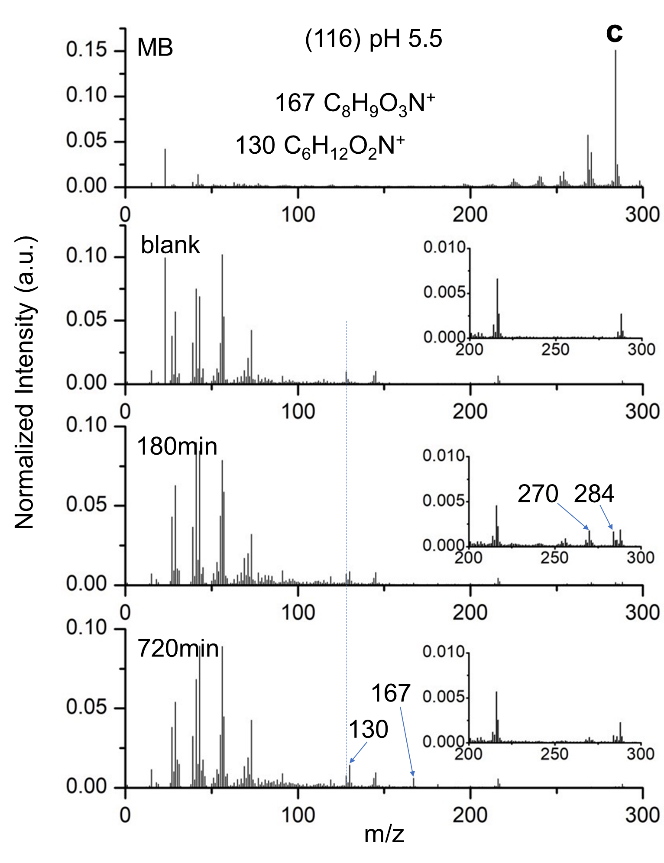


**Figure S10.** Positive ion spectra (m/z range 0-300) from TOF-SIMS recordings at different time of the photoreactions (pH ~ 5.5). Note: m/z 56 is Fe^+^, m/z 216 is Fe_3_O_3_^+^, and m/z 288 is Fe_4_O_4_^+^


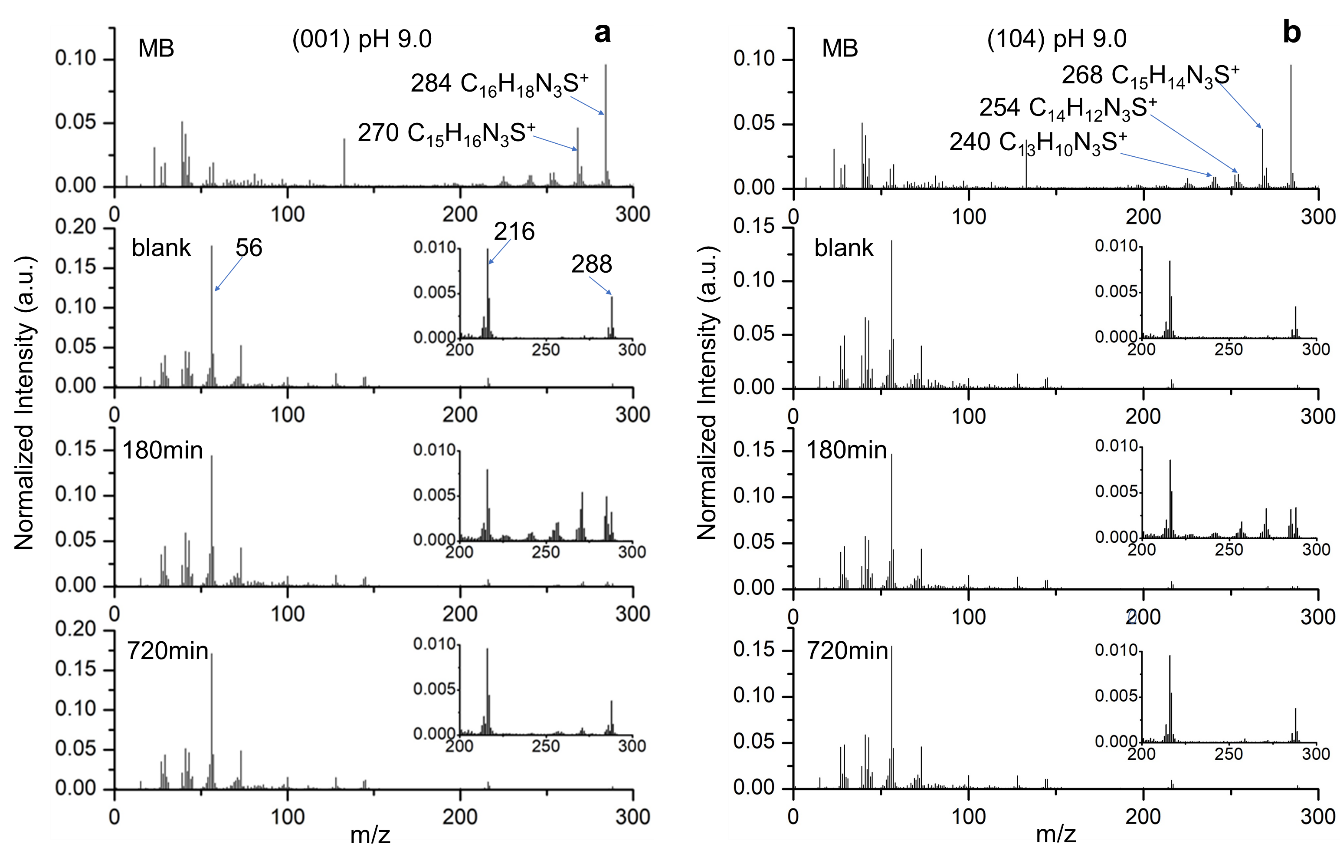


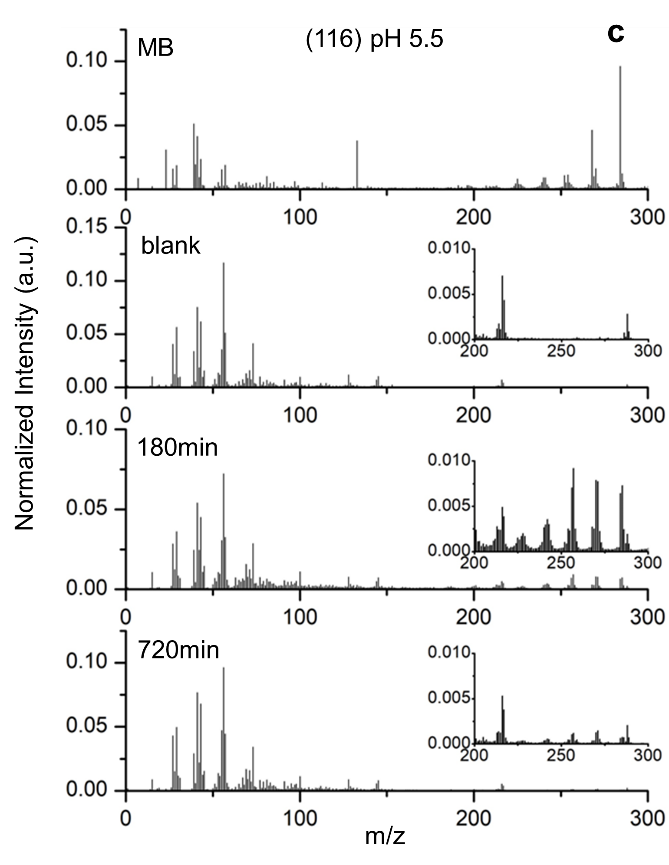


**Figure S11.** Positive ion spectra (m/z range 0-300) from TOF-SIMS recordings at different time of the photoreactions (pH ~ 9.0). Note: m/z 56 is Fe^+^, m/z 216 is Fe_3_O_3_^+^, and m/z 288 is Fe_4_O_4_^+^


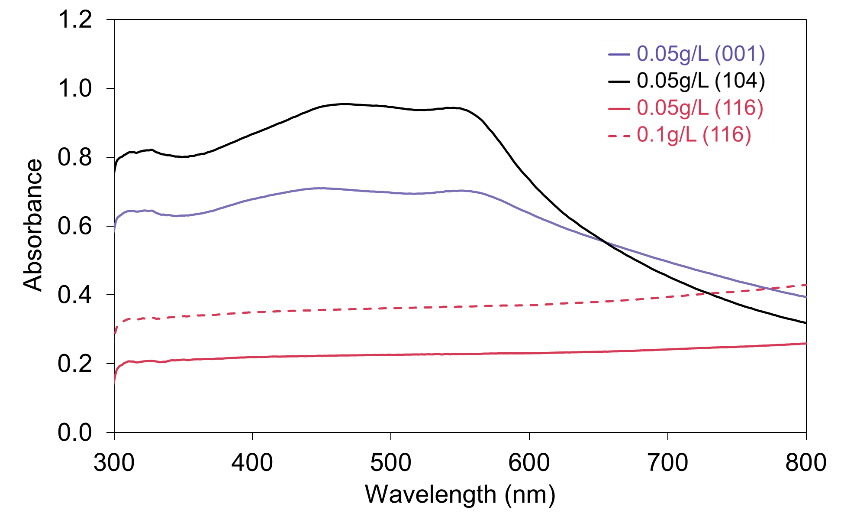


**Figure S12.** UV-vis diffuse absorption spectra of three different hematite nanoparticle


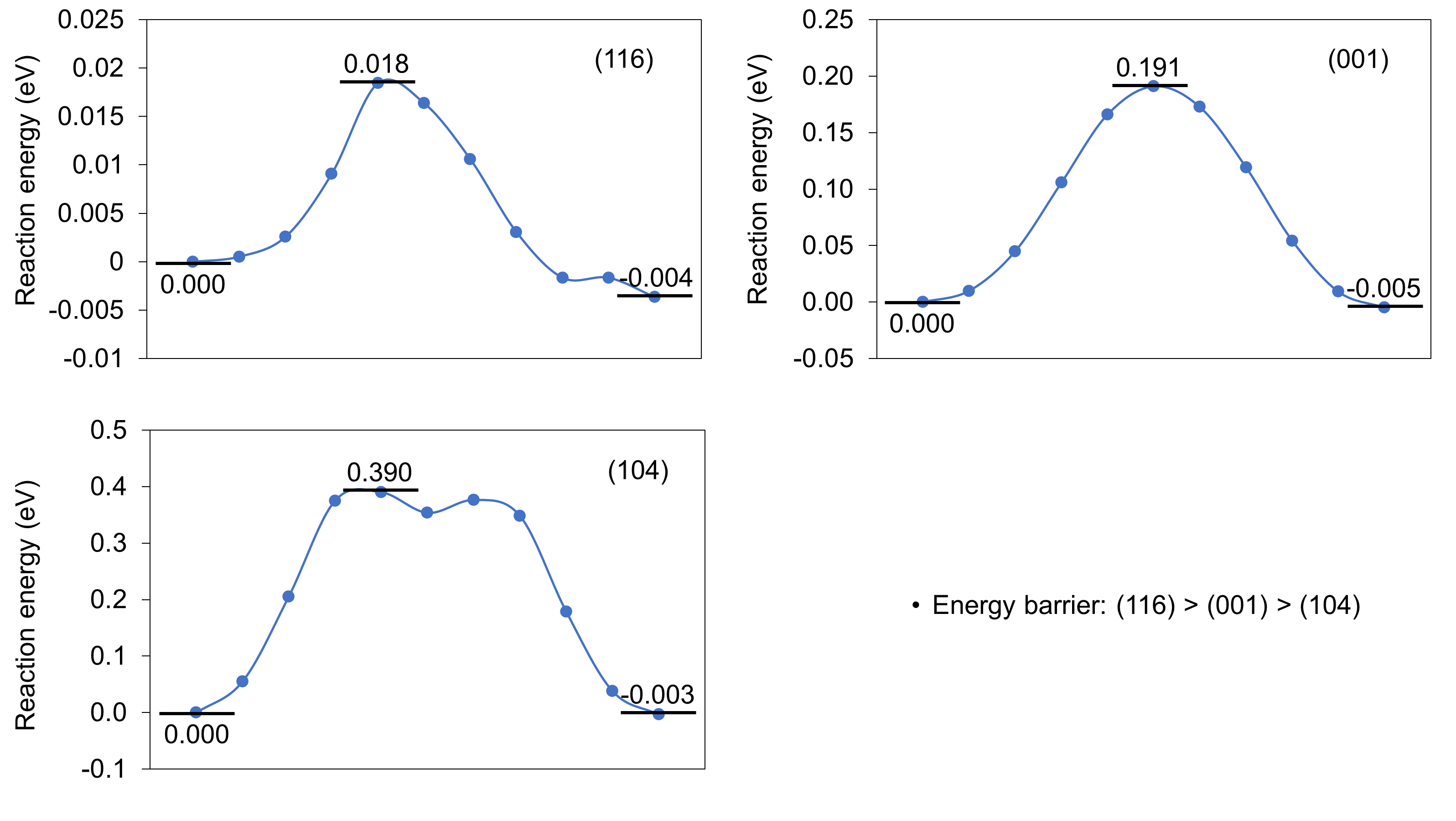


**Figure S13.** Energy barrier of the equation 6 (≡Fe^2+^ + H_2_O_2_ == ≡Fe^3+^ + ∙OH + OH^-^) in Path Two calculated by DFT. More CIF data can be found in **Appendix 1** to **9.**

MB degradation by the three-sets of hematite with coexisted Fe^2+^

This experiment was set to examine the influence of surface Fe^2+^ of hematite on the MB degradation. Experiments were carried out similar to the photoreaction except illumination (in dark and O_2_ free). H_2_O_2_ was added into hematite suspension at a same time with MB, Fe^2+^ or 3 h later. The initial condition is 0.5 g/L hematite, 5 mg/L Fe^2+^, 10 mg/L MB, H_2_O_2_ (V:V) 3:100, pH 5.6.


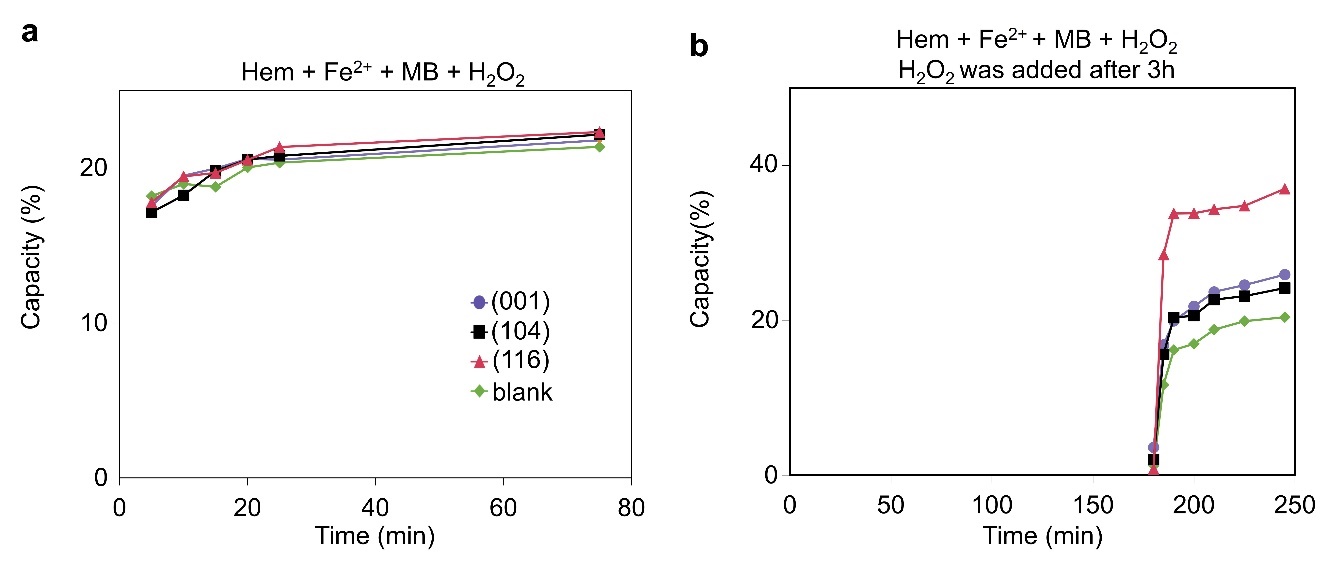


**Figure S14.** MB removal capacity by Fe^2+^ adsorbed Hematite nanoparticles. a, all the components were added at the same time while b, the H_2_O_2_ was added after 3 h of mixing other components.


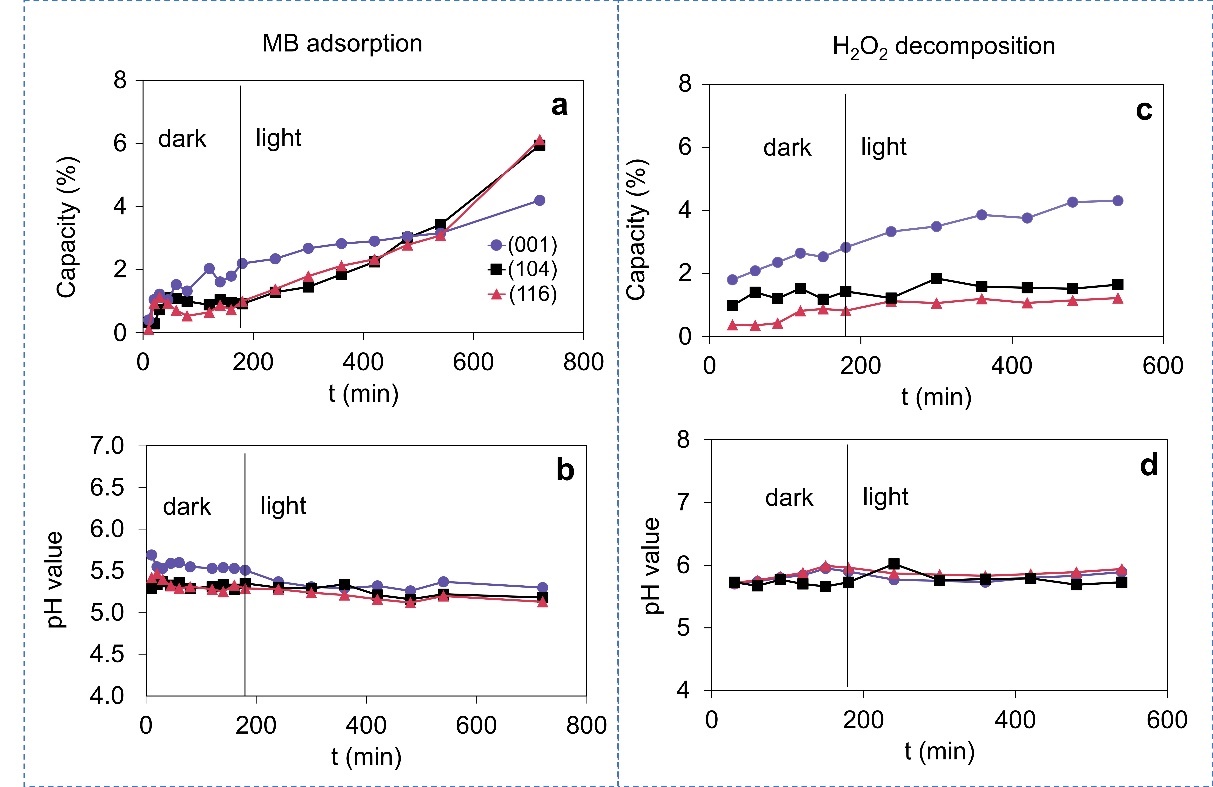


**Figure S15.** Adsorption of MB by three different hematite nanoparticles without H_2_O_2_ (a, b). Decomposition of H_2_O_2_ by three different hematite nanoparticles without MB (c, d)


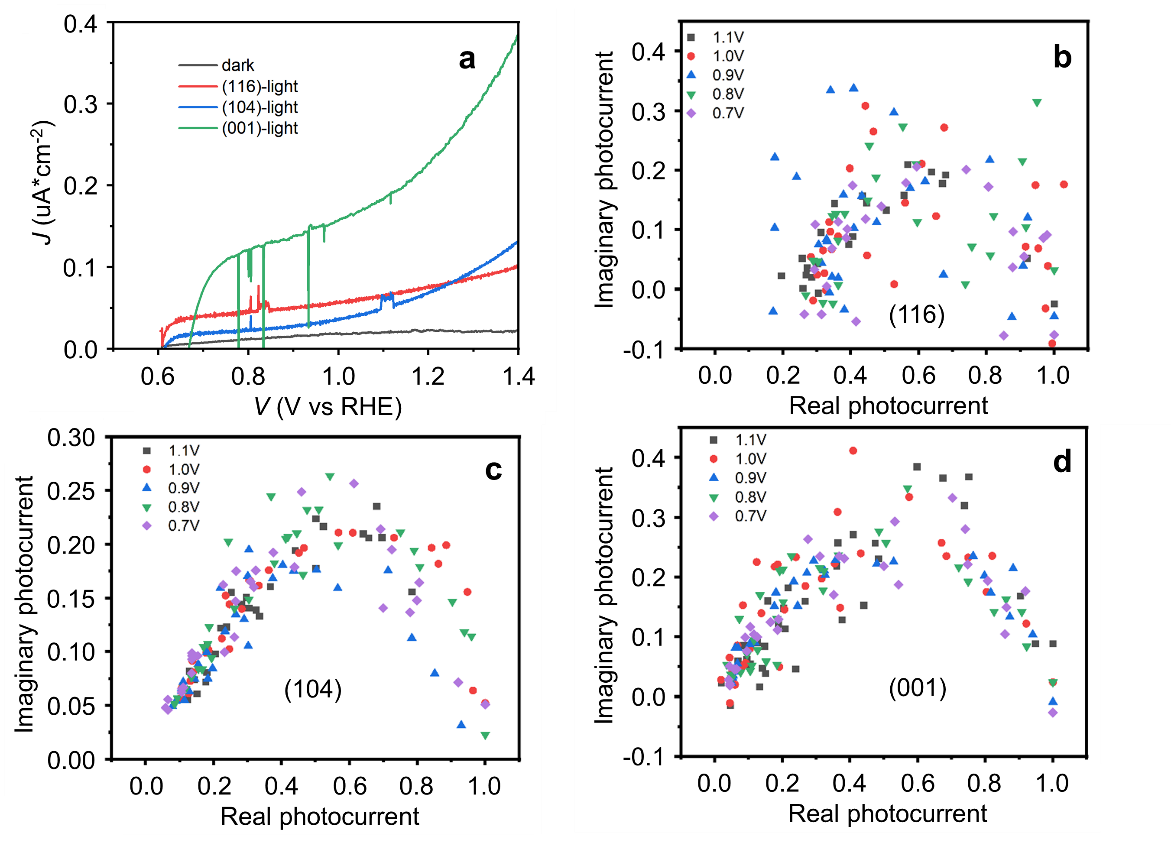


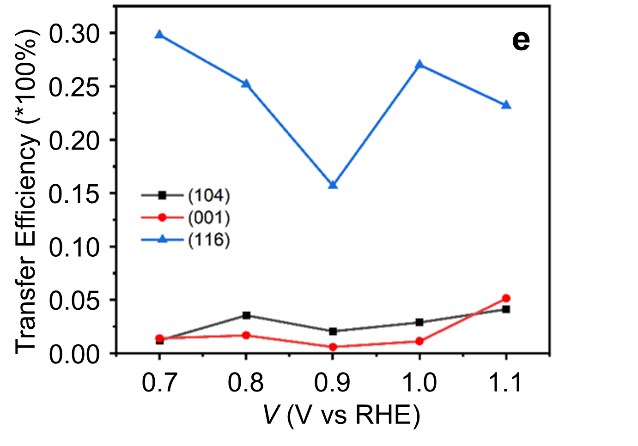


**Figure S16.** PEC and IMPS data for hematite under monochromic illuminations (λ = 405 nm; intensity: 95 mW∙cm^−2^). current–voltage curves for (104), (001), (116) hematite, (b)-(d) Nyquist plots of the (104), (001), (116) hematite at different potential, (e) Charge transfer efficiencies for various hematite photoelectrodes at different applied potentials.


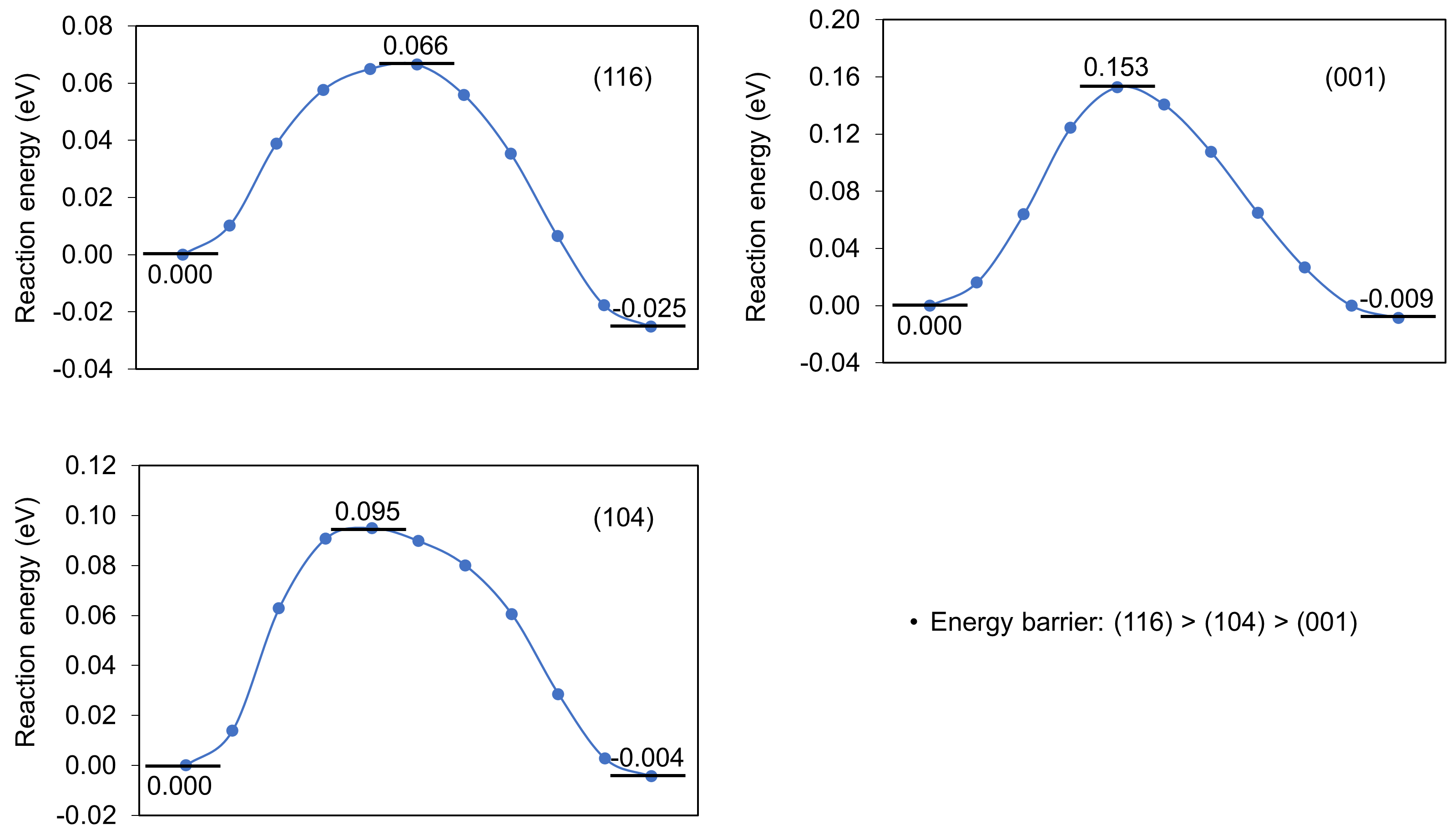


**Figure S17.** Energy barrier of the equation 7 (≡Fe^3+^ + H_2_O_2_ == ≡Fe…OOH^2+^ + H^+^ == ≡Fe^2+^ + ∙OOH + H^+^) in Path Three calculated by DFT. More CIF data can be found in **Appendix 10** to **18.**

**Table S6.** Comparative Fenton and Photo-Fenton Degradation Performance of Various Catalysts Reported in Literature and This Study.

| **Catalysts** | **pH** | **H_2_O_2_ dose (M)** | **Temperature (°C)** | **Time (h)** | **Sun source** | **Fenton reaction (mg/g)** |
| --- | --- | --- | --- | --- | --- | --- |
| Fe_2_O_3_/TiO_2_-BC^[20]^ | 6.0 | 1.18 | 30 | 1 | -- | 248 |
| Fe_2_O_3_/TiO_2_/RGO^[21]^ | 8.0 | 0.48 | 35 | 1 | -- | 760 |
| TiO_2_-doped Fe_3_O_4_ NPs^[22]^ | 3.5 | 0.25 | 35 | 2 | -- | 41 |
| Fe_3_O_4_^[23]^ | 2.5 | 0.06 | n.m. | 1 | -- | 66.7 |
| Fe_3_O_4_@MAFCC^[23]^ | 2.5 | 0.06 | n.m. | 1 | -- | 83.3 |
| Fe_3_O_4_/usGO^[24]^ | 3.0 | n.m. | 60 | 2 | -- | 1712 |
| H(001) this study | 9.0 | 0.29 | 25 | 96 | -- | 19.1 |
| **Catalysts** | **pH** | **H_2_O_2_ dose (M)** | **Temperature (°C)** | **Time (h)** | **Sun source** | **Photo-Fenton reaction (mg/g)** |
| Fe_3_O_4_-rGO-ZnO^[25]^ | 7.0 | 0.0556 | 25 | 2 | vis | 12.4 |
| Mn_3_O_4_/Fe_3_O_4_/CMS^[26]^ | 3.0 | 0.03 | 30 | 1 | vis | 83.2 |
| Fe_3_O_4_^[27]^ | 7.0 | 0.176 | 25 | 2 | vis | 3.2 |
| Fe_3_O_4_@rGO@TiO_2_^[27]^ | 7.0 | 0.176 | 25 | 2 | vis | 6.3 |
| TiO_2_^[28]^ | n.m. | 0.0098 | n.m. | 3.5 | UV | 0.06 |
| Fe-C-TiO_2_-500^[28]^ | n.m. | 0.0098 | n.m. | 3.5 | UV | 0.15 |
| H(104) this study | 9.0 | 0.29 | 25 | 7 | vis | 19.9 |
| H(104) this study | 5.5 | 0.29 | 25 | 7 | vis | 11.6 |
| Note: n.m. represents no mentioned | | | | | | |

**REFERENCES**

[1] Y. Wang, S. Xue, Q. Lin, et al.,"Particle-based hematite crystallization is invariant to initial particle morphology," *Proceedings of the National Academy of Sciences of the United States of America* **2022**, *119* (11), e2112679119.

[2] E. Apra, E. J. Bylaska, W. A. De Jong, et al.,"NWChem: Past, present, and future," *The Journal of Chemical Physics* **2020**, *152* (18).

[3] M. Valiev, E. J. Bylaska, N. Govind, et al.,"NWChem: A comprehensive and scalable open-source solution for large scale molecular simulations," *Computer Physics Communications* **2010**, *181* (9), 1477.

[4] J. P. Perdew, K. Burke, M. Ernzerhof,"Generalized gradient approximation made simple," *Physical Review Letters* **1996**, *77* (18), 3865.

[5] S. L. Dudarev, G. A. Botton, S. Y. Savrasov, C. Humphreys, A. P. Sutton,"Electron-energy-loss spectra and the structural stability of nickel oxide: An LSDA+ U study," *Physical Review B* **1998**, *57* (3), 1505.

[6] J. D. Kubicki, K. W. Paul, D. L. Sparks,"Periodic density functional theory calculations of bulk and the (010) surface of goethite," *Geochemical Transactions* **2008**, *9* (1), 1.

[7] J. D. Kubicki, D. Tunega, S. Kraemer,"A density functional theory investigation of oxalate and Fe (II) adsorption onto the (010) goethite surface with implications for ligand-and reduction-promoted dissolution," *Chemical Geology* **2017**, *464*, 14.

[8] D. Hamann,"Generalized norm-conserving pseudopotentials," *Physical Review B* **1989**, *40* (5), 2980.

[9] N. Troullier, J. L. Martins,"Efficient pseudopotentials for plane-wave calculations," *Physical Review B* **1991**, *43* (3), 1993.

[10] L. Kleinman, D. Bylander,"Efficacious form for model pseudopotentials," *Physical Review Letters* **1982**, *48* (20), 1425.

[11] E. J. Bylaska, D. Song, K. M. Rosso,"Electron transfer calculations between edge sharing octahedra in hematite, goethite, and annite," *Geochimica et Cosmochimica Acta* **2020**, *291*, 79.

[12] V. Ásgeirsson, B. O. Birgisson, R. Bjornsson, et al.,"Nudged elastic band method for molecular reactions using energy-weighted springs combined with eigenvector following," *Journal of Chemical Theory and Computation* **2021**, *17* (8), 4929.

[13] G. Henkelman, H. Jónsson,"Improved tangent estimate in the nudged elastic band method for finding minimum energy paths and saddle points," *The Journal of chemical physics* **2000**, *113* (22), 9978.

[14] G. Henkelman, B. P. Uberuaga, H. Jónsson,"A climbing image nudged elastic band method for finding saddle points and minimum energy paths," *The Journal of chemical physics* **2000**, *113* (22), 9901.

[15] G. Mills, H. Jónsson,"Quantum and thermal effects in H_2_ dissociative adsorption: Evaluation of free energy barriers in multidimensional quantum systems," *Physical Review Letters* **1994**, *72* (7), 1124.

[16] G. Mills, H. Jónsson, G. K. Schenter,"Reversible work transition state theory: application to dissociative adsorption of hydrogen," *Surface Science* **1995**, *324* (2-3), 305.

[17] S. Smidstrup, A. Pedersen, K. Stokbro, H. Jónsson,"Improved initial guess for minimum energy path calculations," *The Journal of chemical physics* **2014**, *140* (21), 214106.

[18] M. Zong, D. Song, X. Zhang, et al.,"Facet-dependent photodegradation of methylene blue by hematite nanoplates in visible light," *Environmental Science & Technology* **2020**, *55* (1), 677.

[19] C. Frejaville, H. Karoui, B. Tuccio, et al.,"5-Diethoxyphosphoryl-5-methyl-1-pyrroline N-oxide (DEPMPO): a new phosphorylated nitrone for the efficient In Vitro and In Vivo spin trapping of oxygen-centred radicals," *Journal of the Chemical Society, Chemical Communications* **1994**, (15), 1793.

[20] X.-L. Chen, F. Li, H. Chen, H. Wang, G. Li,"Fe_2_O_3_/TiO_2_ functionalized biochar as a heterogeneous catalyst for dyes degradation in water under Fenton processes," *Journal of Environmental Chemical Engineering* **2020**, *8* (4), 103905.

[21] C. Sun, S.-T. Yang, Z. Gao, et al.,"Fe_3_O_4_/TiO_2_/reduced graphene oxide composites as highly efficient Fenton-like catalyst for the decoloration of methylene blue," *Materials Chemistry and Physics* **2019**, *223*, 751.

[22] S. Yang, L. Yang, X. Liu, et al.,"TiO_2_-doped Fe_3_O_4_ nanoparticles as high-performance Fenton-like catalyst for dye decoloration," *Science China Technological Sciences* **2015**, *58*, 858.

[23] Q. Lu, Y. Zhang, H. Hu, et al.,"In situ synthesis of a stable Fe_3_O_4_@ cellulose nanocomposite for efficient catalytic degradation of methylene blue," *Nanomaterials* **2019**, *9* (2), 275.

[24] S. Song, Y. Wang, H. Shen, et al.,"Ultrasmall graphene oxide modified with Fe_3_O_4_ nanoparticles as a fenton-like agent for methylene blue degradation," *ACS Applied Nano Materials* **2019**, *2* (11), 7074.

[25] A. Abharya, A. Gholizadeh,"Synthesis of a Fe_3_O_4_-rGO-ZnO-catalyzed photo-Fenton system with enhanced photocatalytic performance," *Ceramics International* **2021**, *47* (9), 12010.

[26] T. D. Nguyen, X. M. Vu, T. F. Kouznetsova, et al.,"Advanced Mn_3_O_4_/Fe_3_O_4_-carbon molecular sieve composite: a robust catalyst for heterogeneous photo-fenton oxidation of organic dyes," *Journal of Porous Materials* **2025**, *32* (3), 821.

[27] X. Yang, W. Chen, J. Huang, et al.,"Rapid degradation of methylene blue in a novel heterogeneous Fe_3_O_4_@rGO@TiO_2_-catalyzed photo-Fenton system," *Scientific reports* **2015**, *5* (1), 10632.

[28] B. Tryba, M. Piszcz, B. Grzmil, A. Pattek-Janczyk, A. W. Morawski,"Photodecomposition of dyes on Fe-C-TiO_2_ photocatalysts under UV radiation supported by photo-Fenton process," *Journal of Hazardous Materials* **2009**, *162* (1), 111.

**Appendix 1. CIF file of (116) at the initial state (equation 6, and Fig. S13).**

_cell_length_a 7.4825

_cell_length_b 9.0497

_cell_length_c 27.0000

_cell_angle_alpha 110.1837

_cell_angle_beta 95.8323

_cell_angle_gamma 63.6274

_symmetry_space_group_name_H-M P1

loop_

_atom_site_type_symbol

_atom_site_fract_x

_atom_site_fract_y

_atom_site_fract_z

O 0.984610 0.332989 0.231330

O 0.640502 0.673189 0.246216

O 0.023657 0.931320 0.280655

O 0.359617 0.586545 0.269483

O 0.704224 0.217962 0.251591

Fe 0.544305 0.423312 0.307461

O 0.621797 0.358081 0.379982

O 0.725502 0.545723 0.334481

Fe 0.023208 0.416576 0.305499

Fe 0.805044 0.470014 0.401955

O 0.976486 0.338697 0.451654

O 0.326868 0.337131 0.314518

Fe 0.668236 0.780974 0.325762

O 0.645594 0.008304 0.312850

Fe 0.858275 0.792832 0.454832

Fe 0.163074 0.790679 0.330400

Fe 0.693302 0.125727 0.388412

Fe 0.312321 0.467640 0.402302

O 0.648130 0.697768 0.456937

O 0.359195 0.908179 0.335294

O 0.690352 0.920672 0.406881

O 0.018816 0.557236 0.400795

O 0.047564 0.899645 0.479187

O 0.313177 0.666983 0.381169

O 0.990299 0.024624 0.388594

O 0.983429 0.661743 0.306239

Fe 0.353166 0.794134 0.456013

O 0.347892 0.596417 0.478381

Fe 0.979064 0.133171 0.475565

Fe 0.186835 0.138249 0.394107

O 0.034061 0.234059 0.336158

O 0.685023 0.234848 0.474000

Fe 0.488376 0.129099 0.474548

O 0.383663 0.234395 0.409611

O 0.289105 0.031333 0.453834

Fe 0.511481 0.471639 0.526679

O 0.616551 0.365008 0.591066

O 0.710918 0.568826 0.546971

Fe 0.020907 0.467736 0.525736

Fe 0.813124 0.461835 0.607038

O 0.965893 0.365655 0.664619

O 0.315073 0.364773 0.526663

Fe 0.646578 0.806559 0.545243

O 0.652221 0.003848 0.522520

Fe 0.838061 0.808493 0.670475

Fe 0.141670 0.807863 0.546305

Fe 0.687303 0.132960 0.599152

Fe 0.307130 0.474363 0.612634

O 0.642111 0.691891 0.665279

O 0.351482 0.902966 0.543930

O 0.687275 0.932984 0.619908

O 0.010104 0.574993 0.611615

O 0.013452 0.940590 0.695687

O 0.309637 0.678246 0.593372

O 0.980922 0.042927 0.599957

O 0.952363 0.700475 0.521286

Fe 0.330831 0.818773 0.675062

O 0.353503 0.590180 0.687882

Fe 0.977706 0.183126 0.695727

Fe 0.195211 0.130862 0.599678

O 0.023750 0.261155 0.549219

O 0.672440 0.263275 0.686855

Fe 0.454466 0.178785 0.694563

O 0.378485 0.242554 0.621477

O 0.274311 0.057062 0.666876

O 0.294528 0.380345 0.751278

O 0.635160 0.012108 0.733429

O 0.362343 0.927475 0.754222

O 0.976132 0.665849 0.719313

O 0.009638 0.272702 0.769968

H 0.113742 0.178563 0.779728

H 0.372374 0.856661 0.774216

H 0.544073 0.977605 0.745960

H 0.936894 0.722033 0.756089

H 0.188740 0.365537 0.763520

H 0.238346 0.611854 0.708039

H 0.676713 0.668617 0.697762

H 0.589728 0.387510 0.693943

H 0.999081 0.460735 0.688959

H 0.019197 0.954996 0.732647

H 0.808624 0.229020 0.237381

H 0.997971 0.141543 0.312038

H 0.758011 0.988308 0.292019

H 0.056268 0.879219 0.243809

H 0.618181 0.751900 0.227964

H 0.444012 0.618084 0.254014

H 0.871440 0.426033 0.223312

H 0.323951 0.932750 0.303037

H 0.982051 0.644617 0.269263

H 0.409956 0.213049 0.307245

O 0.891291 0.900951 0.830571

H 0.032855 0.842770 0.831659

O 0.875386 0.043903 0.813473

H 0.772029 0.044682 0.787381

**Appendix 2. CIF file of (116) at the transition state (equation 6, and Fig. S13).**

_cell_length_a 7.4825

_cell_length_b 9.0497

_cell_length_c 27.0000

_cell_angle_alpha 110.1837

_cell_angle_beta 95.8323

_cell_angle_gamma 63.6274

_symmetry_space_group_name_H-M P1

loop_

_atom_site_type_symbol

_atom_site_fract_x

_atom_site_fract_y

_atom_site_fract_z

O 0.987486 0.330559 0.230932

O 0.640830 0.673458 0.245960

O 0.024266 0.931265 0.280324

O 0.359548 0.587021 0.269242

O 0.703577 0.218722 0.251334

Fe 0.544459 0.424070 0.307195

O 0.621815 0.358684 0.379805

O 0.725854 0.545985 0.334182

Fe 0.023339 0.416772 0.305086

Fe 0.805290 0.470137 0.401539

O 0.976338 0.339180 0.451708

O 0.326951 0.337812 0.314410

Fe 0.668222 0.781371 0.325370

O 0.645710 0.009042 0.312760

Fe 0.857906 0.792849 0.454548

Fe 0.162896 0.790781 0.330033

Fe 0.693192 0.125946 0.388158

Fe 0.312471 0.467735 0.401941

O 0.648034 0.698269 0.456788

O 0.358738 0.908863 0.335148

O 0.690594 0.921146 0.406847

O 0.018988 0.557611 0.400770

O 0.047486 0.899897 0.479127

O 0.313167 0.667503 0.381080

O 0.990124 0.025391 0.388408

O 0.983455 0.661865 0.305826

Fe 0.353342 0.794045 0.455750

O 0.347795 0.596818 0.478437

Fe 0.979001 0.133239 0.475357

Fe 0.186959 0.138274 0.393855

O 0.033807 0.234706 0.336102

O 0.684743 0.235542 0.473999

Fe 0.488405 0.129018 0.474375

O 0.383685 0.234797 0.409514

O 0.289028 0.031573 0.453744

Fe 0.511577 0.471567 0.526418

O 0.615889 0.365764 0.591073

O 0.710379 0.569351 0.546980

Fe 0.020254 0.468218 0.525581

Fe 0.813241 0.461918 0.606895

O 0.967124 0.364823 0.664442

O 0.314609 0.365757 0.526683

Fe 0.646222 0.807266 0.545312

O 0.651993 0.004338 0.522603

Fe 0.838822 0.809785 0.671405

Fe 0.140891 0.808555 0.546142

Fe 0.686890 0.133193 0.599153

Fe 0.306000 0.473890 0.612252

O 0.639101 0.691991 0.665190

O 0.350786 0.903684 0.544009

O 0.687350 0.933088 0.619970

O 0.009344 0.575468 0.611530

O 0.019794 0.939138 0.696018

O 0.309374 0.680271 0.594276

O 0.980214 0.043572 0.599730

O 0.952225 0.701008 0.521334

Fe 0.331068 0.819296 0.675674

O 0.351779 0.593023 0.687809

Fe 0.974826 0.185577 0.696089

Fe 0.193955 0.131389 0.599501

O 0.023422 0.261704 0.549243

O 0.671892 0.263134 0.686373

Fe 0.454426 0.178748 0.694626

O 0.377794 0.242589 0.621279

O 0.274341 0.053046 0.666585

O 0.294658 0.381765 0.750509

O 0.636902 0.013724 0.732102

O 0.379697 0.918543 0.751429

O 0.974333 0.663518 0.718322

O 0.001345 0.284317 0.769604

H 0.072119 0.193289 0.784371

H 0.342374 0.885832 0.776902

H 0.550783 0.979171 0.746389

H 0.949472 0.720971 0.756253

H 0.189602 0.369192 0.764033

H 0.238598 0.612350 0.708402

H 0.675597 0.670431 0.698008

H 0.589964 0.387480 0.694158

H 0.998766 0.461620 0.689068

H 0.015531 0.957141 0.734258

H 0.808638 0.228884 0.237105

H 0.997931 0.142194 0.311859

H 0.757834 0.988768 0.291802

H 0.057584 0.878276 0.243505

H 0.617630 0.752366 0.227728

H 0.444203 0.618321 0.253720

H 0.875724 0.422855 0.222246

H 0.323161 0.932966 0.302824

H 0.981456 0.645163 0.268870

H 0.410179 0.213640 0.307008

O 0.933772 0.866212 0.826206

H 0.074779 0.812560 0.827858

O 0.941356 0.033892 0.805250

H 0.810816 0.047484 0.794020

**Appendix 3. CIF file of (116) at the end state (equation 6, and Fig. S13).**

_cell_length_a 7.4825

_cell_length_b 9.0497

_cell_length_c 27.0000

_cell_angle_alpha 110.1837

_cell_angle_beta 95.8323

_cell_angle_gamma 63.6274

_symmetry_space_group_name_H-M P1

loop_

_atom_site_type_symbol

_atom_site_fract_x

_atom_site_fract_y

_atom_site_fract_z

O 0.991829 0.325210 0.230120

O 0.640950 0.674963 0.245968

O 0.025403 0.930059 0.279557

O 0.359187 0.586857 0.268599

O 0.703525 0.218594 0.250981

Fe 0.544246 0.424480 0.306862

O 0.622142 0.358380 0.378713

O 0.725763 0.548707 0.332914

Fe 0.025770 0.414146 0.304420

Fe 0.805979 0.470472 0.401660

O 0.976149 0.338538 0.450807

O 0.327171 0.337549 0.313609

Fe 0.667533 0.782302 0.325139

O 0.647399 0.006196 0.312068

Fe 0.857928 0.793271 0.454768

Fe 0.162369 0.791005 0.329882

Fe 0.693493 0.127179 0.388421

Fe 0.312399 0.468517 0.401999

O 0.648124 0.697244 0.455687

O 0.361112 0.907956 0.334332

O 0.691012 0.918020 0.404877

O 0.019129 0.556581 0.399930

O 0.047206 0.899294 0.478215

O 0.313010 0.666901 0.379995

O 0.990467 0.024458 0.387283

O 0.980709 0.661873 0.304921

Fe 0.353275 0.794442 0.455939

O 0.347914 0.595848 0.477434

Fe 0.979137 0.133736 0.475608

Fe 0.186754 0.139050 0.393986

O 0.033060 0.234982 0.335387

O 0.684809 0.234252 0.472992

Fe 0.487345 0.130608 0.474730

O 0.384135 0.234135 0.408642

O 0.289214 0.030844 0.452824

Fe 0.511027 0.472774 0.526756

O 0.615226 0.365770 0.590336

O 0.709964 0.569133 0.546211

Fe 0.019598 0.469363 0.525922

Fe 0.812649 0.463265 0.607254

O 0.967952 0.363788 0.663687

O 0.314262 0.365376 0.525817

Fe 0.645696 0.808308 0.545676

O 0.651748 0.003430 0.521485

Fe 0.840365 0.811077 0.672086

Fe 0.140808 0.809295 0.546583

Fe 0.686425 0.134148 0.599357

Fe 0.304898 0.474968 0.612587

O 0.639188 0.690543 0.664730

O 0.350381 0.902826 0.543075

O 0.686558 0.932606 0.618804

O 0.009094 0.573764 0.610565

O 0.021521 0.938653 0.694684

O 0.309392 0.679747 0.593474

O 0.979437 0.042968 0.598667

O 0.951861 0.700862 0.520597

Fe 0.330951 0.820712 0.676600

O 0.350717 0.592462 0.686906

Fe 0.973832 0.186481 0.696626

Fe 0.193270 0.131824 0.599843

O 0.022913 0.261218 0.548334

O 0.671473 0.262527 0.685372

Fe 0.453647 0.178015 0.694128

O 0.377327 0.241881 0.620350

O 0.273028 0.052695 0.665786

O 0.294473 0.383034 0.748889

O 0.638954 0.015288 0.730474

O 0.405944 0.895394 0.750260

O 0.971368 0.660137 0.716667

O 0.991220 0.298224 0.769444

H 0.009039 0.221683 0.789157

H 0.301764 0.937840 0.776411

H 0.557532 0.979845 0.746351

H 0.969066 0.715228 0.755557

H 0.190170 0.374305 0.764168

H 0.239977 0.613177 0.708576

H 0.675581 0.671136 0.697987

H 0.589983 0.387299 0.694017

H 0.999818 0.460855 0.688759

H 0.015602 0.959486 0.735189

H 0.808954 0.226516 0.236108

H 0.997563 0.142372 0.311255

H 0.758555 0.987476 0.291008

H 0.059393 0.876093 0.242772

H 0.617089 0.751888 0.226928

H 0.443381 0.617194 0.252696

H 0.882474 0.414772 0.219906

H 0.322415 0.931770 0.302026

H 0.980820 0.644495 0.267923

H 0.409922 0.213615 0.306174

O 0.991601 0.811989 0.819045

H 0.133429 0.760955 0.820736

O 0.021961 0.012258 0.792802

H 0.890738 0.051414 0.806562

**Appendix 4. CIF file of (001) at the initial state (equation 6, and Fig. S13).**

_cell_length_a 10.1955

_cell_length_b 10.1955

_cell_length_c 24.2762

_cell_angle_alpha 90.0000

_cell_angle_beta 90.0000

_cell_angle_gamma 60.0000

_symmetry_space_group_name_H-M P1

loop_

_atom_site_type_symbol

_atom_site_fract_x

_atom_site_fract_y

_atom_site_fract_z

Fe 0.055132 0.917210 0.658007

Fe 0.221269 0.086046 0.640540

Fe 0.222271 0.086691 0.462757

Fe 0.888667 0.252374 0.439179

Fe 0.889256 0.754124 0.562630

Fe 0.061261 0.914171 0.538525

Fe 0.055786 0.919652 0.361284

Fe 0.222418 0.089422 0.341289

O 0.062338 0.242053 0.701053

O 0.879789 0.092893 0.697122

O 0.209924 0.760717 0.603837

O 0.056196 0.072673 0.600675

O 0.896650 0.919436 0.602294

O 0.242601 0.904335 0.498035

O 0.885844 0.106163 0.500060

O 0.040540 0.747590 0.500413

O 0.875841 0.933789 0.401517

O 0.223381 0.239061 0.400134

O 0.068710 0.086442 0.400250

O 0.901839 0.069588 0.300167

O 0.053915 0.763260 0.300847

H 0.968399 0.241506 0.699541

H 0.961412 0.760041 0.301929

H 0.860427 0.064397 0.732296

H 0.922059 0.995178 0.272668

O 0.219409 0.932437 0.700584

H 0.169351 0.003781 0.729325

O 0.220191 0.912952 0.304159

H 0.168797 0.937996 0.269902

Fe 0.556823 0.915861 0.658676

Fe 0.720111 0.086821 0.641679

Fe 0.723261 0.086457 0.464507

Fe 0.389872 0.252668 0.439130

Fe 0.378686 0.757643 0.561442

Fe 0.554494 0.918118 0.537670

Fe 0.556690 0.919342 0.360562

Fe 0.721450 0.090347 0.342672

O 0.562497 0.242842 0.700395

O 0.379944 0.093662 0.696209

O 0.706350 0.766104 0.599493

O 0.555335 0.071841 0.600019

O 0.398982 0.921194 0.599720

O 0.736063 0.905320 0.500779

O 0.389246 0.101120 0.500302

O 0.542692 0.752050 0.499813

O 0.376592 0.933393 0.399724

O 0.720692 0.240525 0.400758

O 0.568989 0.085035 0.401084

O 0.402780 0.068787 0.298830

O 0.555606 0.762014 0.300200

H 0.468798 0.242011 0.699243

H 0.463153 0.758742 0.301522

H 0.355474 0.071628 0.731363

H 0.424225 0.993306 0.271634

O 0.719916 0.930993 0.700385

H 0.677497 0.993235 0.731981

O 0.720350 0.914485 0.304225

H 0.668711 0.942317 0.270019

Fe 0.052851 0.415465 0.659070

Fe 0.220893 0.583305 0.642421

Fe 0.221442 0.585235 0.463353

Fe 0.889504 0.752842 0.439403

Fe 0.888708 0.248808 0.561735

Fe 0.053769 0.420224 0.538199

Fe 0.055490 0.418932 0.360838

Fe 0.220583 0.590035 0.341925

O 0.055842 0.747839 0.703332

O 0.875653 0.594001 0.695496

O 0.206547 0.266940 0.600544

O 0.054625 0.571813 0.600887

O 0.898432 0.419590 0.599595

O 0.235710 0.404160 0.500590

O 0.887699 0.600134 0.500631

O 0.041341 0.253365 0.500293

O 0.875744 0.433021 0.400944

O 0.222077 0.738883 0.400462

O 0.068376 0.584825 0.400567

O 0.901989 0.568774 0.299505

O 0.055003 0.262289 0.300241

H 0.959705 0.752511 0.698853

H 0.963188 0.257800 0.301570

H 0.848188 0.571837 0.730083

H 0.922601 0.494150 0.272049

O 0.210290 0.436950 0.702759

H 0.154509 0.509628 0.730530

O 0.219841 0.413510 0.304155

H 0.168473 0.439536 0.269889

Fe 0.556498 0.415930 0.658975

Fe 0.717851 0.586759 0.638826

Fe 0.722314 0.585489 0.463179

Fe 0.389180 0.754141 0.438603

Fe 0.388406 0.252140 0.562486

Fe 0.553881 0.422161 0.538293

Fe 0.554783 0.420018 0.360816

Fe 0.721548 0.589596 0.341936

O 0.559739 0.744541 0.699620

O 0.379075 0.583519 0.700031

O 0.706917 0.266344 0.600605

O 0.551907 0.572887 0.599554

O 0.398249 0.419917 0.600822

O 0.734382 0.406747 0.500473

O 0.386901 0.601238 0.500352

O 0.539841 0.254359 0.500865

O 0.375306 0.433158 0.400950

O 0.722958 0.739262 0.400308

O 0.568651 0.585385 0.400511

O 0.401151 0.569112 0.299463

O 0.554736 0.262227 0.300879

H 0.461692 0.752942 0.699029

H 0.462680 0.258188 0.301873

H 0.362888 0.542631 0.732730

H 0.421847 0.494220 0.272110

O 0.714454 0.439387 0.701165

H 0.658425 0.516066 0.727796

O 0.719625 0.413581 0.304236

H 0.668521 0.439847 0.269919

O 0.899940 0.969102 0.812124

O 0.029345 0.814681 0.811415

H 0.949156 0.023407 0.823126

H 0.041216 0.793393 0.771248

**Appendix 5. CIF file of (001) at the transition state (equation 6, and Fig. S13).**

_cell_length_a 10.1955

_cell_length_b 10.1955

_cell_length_c 24.2762

_cell_angle_alpha 90.0000

_cell_angle_beta 90.0000

_cell_angle_gamma 60.0000

_symmetry_space_group_name_H-M P1

loop_

_atom_site_type_symbol

_atom_site_fract_x

_atom_site_fract_y

_atom_site_fract_z

Fe 0.055560 0.916451 0.659124

Fe 0.219842 0.084017 0.641459

Fe 0.220341 0.085318 0.463226

Fe 0.886830 0.251903 0.439083

Fe 0.886665 0.753000 0.562553

Fe 0.056193 0.915900 0.538415

Fe 0.053810 0.918770 0.361149

Fe 0.220069 0.088852 0.341754

O 0.060429 0.241229 0.699336

O 0.879513 0.092140 0.695560

O 0.206947 0.761504 0.601189

O 0.056119 0.070681 0.599287

O 0.897080 0.919425 0.600463

O 0.237456 0.903369 0.498847

O 0.885172 0.102600 0.500028

O 0.038538 0.749323 0.500089

O 0.873785 0.933012 0.401309

O 0.220873 0.238450 0.400399

O 0.066911 0.084919 0.400572

O 0.899926 0.069686 0.300226

O 0.052074 0.762481 0.300966

H 0.968867 0.236123 0.698967

H 0.959389 0.759560 0.302013

H 0.870914 0.072764 0.734411

H 0.920215 0.996402 0.272274

O 0.222134 0.928017 0.698695

H 0.182200 -0.020919 0.733433

O 0.219328 0.911848 0.304463

H 0.169320 0.935987 0.269937

Fe 0.555780 0.916069 0.659250

Fe 0.721881 0.084854 0.642155

Fe 0.720743 0.085158 0.464081

Fe 0.387470 0.252095 0.439171

Fe 0.381304 0.754817 0.562026

Fe 0.552728 0.917981 0.538133

Fe 0.554290 0.918690 0.360791

Fe 0.719498 0.089429 0.342417

O 0.560815 0.240296 0.699393

O 0.378905 0.092896 0.696510

O 0.705352 0.764851 0.599654

O 0.555096 0.070422 0.600039

O 0.398533 0.920373 0.599438

O 0.733871 0.904028 0.500371

O 0.386966 0.100015 0.500353

O 0.539779 0.751587 0.499964

O 0.374295 0.932827 0.400440

O 0.719413 0.239269 0.400645

O 0.566984 0.084296 0.401030

O 0.400413 0.069260 0.299559

O 0.552938 0.761936 0.300636

H 0.467627 0.238319 0.698785

H 0.460280 0.758992 0.301823

H 0.352138 0.072532 0.731603

H 0.421372 0.995353 0.271784

O 0.723763 0.925554 0.699120

H 0.690522 0.982652 0.732067

O 0.719353 0.912706 0.304472

H 0.669126 0.938283 0.269990

Fe 0.052018 0.414232 0.658656

Fe 0.217862 0.584238 0.641158

Fe 0.219856 0.584548 0.463400

Fe 0.887341 0.752021 0.439210

Fe 0.886995 0.249111 0.562198

Fe 0.052446 0.418991 0.538014

Fe 0.053635 0.418364 0.360828

Fe 0.218995 0.589238 0.341962

O 0.057323 0.743952 0.700674

O 0.874504 0.589856 0.696007

O 0.205263 0.265270 0.600115

O 0.052765 0.570538 0.599801

O 0.896131 0.419902 0.599114

O 0.233882 0.403424 0.500241

O 0.886023 0.599540 0.500283

O 0.038887 0.252481 0.500033

O 0.873705 0.432602 0.400908

O 0.220264 0.738241 0.400482

O 0.066644 0.584085 0.400636

O 0.899923 0.568961 0.299695

O 0.052847 0.261584 0.300648

H 0.959206 0.752729 0.698429

H 0.960709 0.257646 0.301798

H 0.849890 0.563114 0.730244

H 0.920308 0.495255 0.271893

O 0.210486 0.435146 0.701379

H 0.156154 0.507634 0.729385

O 0.218737 0.412455 0.304289

H 0.168272 0.437575 0.269844

Fe 0.554053 0.413879 0.658925

Fe 0.716190 0.585877 0.639496

Fe 0.720213 0.584687 0.463327

Fe 0.387189 0.752724 0.438814

Fe 0.387064 0.250439 0.562840

Fe 0.552282 0.420057 0.538121

Fe 0.553193 0.419046 0.360804

Fe 0.719505 0.589086 0.341967

O 0.557595 0.744078 0.699031

O 0.376443 0.584503 0.698205

O 0.705090 0.265550 0.600011

O 0.551284 0.571228 0.599310

O 0.396574 0.419642 0.599891

O 0.733008 0.404737 0.500101

O 0.385860 0.599961 0.500074

O 0.538196 0.252625 0.500505

O 0.373536 0.432622 0.400948

O 0.720636 0.738503 0.400438

O 0.566762 0.584470 0.400605

O 0.399430 0.569110 0.299646

O 0.552545 0.261646 0.300970

H 0.458205 0.755217 0.698226

H 0.460281 0.257947 0.301983

H 0.356374 0.550468 0.731712

H 0.419940 0.495144 0.271935

O 0.710994 0.438314 0.701073

H 0.655007 0.513953 0.728015

O 0.718449 0.412736 0.304270

H 0.667922 0.438277 0.269835

O 0.903389 1.013758 0.804841

O 0.092116 0.902462 0.804604

H 0.929317 0.084452 0.816688

H 0.095873 0.845281 0.790644

**Appendix 6. CIF file of (001) at the end state (equation 6, and Fig. S13).**

_audit_creation_date Fri Jan 28 06:07:00 2022

_audit_creation_method generated by PSPW module of NWChem

_cell_length_a 10.1955

_cell_length_b 10.1955

_cell_length_c 24.2762

_cell_angle_alpha 90.0000

_cell_angle_beta 90.0000

_cell_angle_gamma 60.0000

_symmetry_space_group_name_H-M P1

loop_

_atom_site_type_symbol

_atom_site_fract_x

_atom_site_fract_y

_atom_site_fract_z

Fe 0.055988 0.915693 0.660241

Fe 0.218416 0.081988 0.642379

Fe 0.218411 0.083945 0.463696

Fe 0.884993 0.251432 0.438988

Fe 0.884074 0.751876 0.562477

Fe 0.051124 0.917629 0.538306

Fe 0.051833 0.917888 0.361014

Fe 0.217720 0.088283 0.342219

O 0.058519 0.240409 0.697619

O 0.879238 0.091389 0.693997

O 0.203970 0.762293 0.598541

O 0.056042 0.068692 0.597899

O 0.897510 0.919416 0.598632

O 0.232310 0.902405 0.499660

O 0.884500 0.099039 0.499997

O 0.036536 0.751058 0.499765

O 0.871730 0.932237 0.401101

O 0.218366 0.237842 0.400665

O 0.065112 0.083398 0.400895

O 0.898012 0.069786 0.300286

O 0.050233 0.761704 0.301086

H 0.969338 0.230784 0.698406

H 0.957365 0.759120 0.302106

H 0.881389 0.081172 0.736550

H 0.918369 0.997666 0.271889

O 0.224858 0.923597 0.696805

H 0.195007 0.954506 0.737572

O 0.218464 0.910747 0.304768

H 0.169840 0.934020 0.269982

Fe 0.554736 0.916278 0.659824

Fe 0.723651 0.082888 0.642632

Fe 0.718224 0.083859 0.463655

Fe 0.385068 0.251523 0.439212

Fe 0.383922 0.751991 0.562611

Fe 0.550962 0.917844 0.538596

Fe 0.551890 0.918038 0.361020

Fe 0.717546 0.088511 0.342163

O 0.559134 0.237753 0.698392

O 0.377867 0.092133 0.696812

O 0.704354 0.763601 0.599815

O 0.554856 0.069006 0.600060

O 0.398085 0.919555 0.599157

O 0.731679 0.902738 0.499964

O 0.384686 0.098913 0.500405

O 0.536866 0.751126 0.500115

O 0.371999 0.932263 0.401157

O 0.718134 0.238015 0.400533

O 0.564978 0.083559 0.400977

O 0.398045 0.069735 0.300288

O 0.550270 0.761860 0.301072

H 0.466454 0.234670 0.698338

H 0.457406 0.759282 0.302132

H 0.348796 0.073473 0.731854

H 0.418518 0.997441 0.271944

O 0.727609 0.920119 0.697857

H 0.703540 0.972107 0.732153

O 0.718356 0.910929 0.304720

H 0.669539 0.934293 0.269970

Fe 0.051185 0.412999 0.658243

Fe 0.214831 0.585171 0.639895

Fe 0.218271 0.583861 0.463448

Fe 0.885177 0.751201 0.439018

Fe 0.885283 0.249414 0.562662

Fe 0.051122 0.417758 0.537830

Fe 0.051779 0.417796 0.360819

Fe 0.217406 0.588442 0.342000

O 0.058802 0.740068 0.698018

O 0.873354 0.585713 0.696518

O 0.203979 0.263604 0.599686

O 0.050904 0.569266 0.598715

O 0.893830 0.420217 0.598634

O 0.232054 0.402691 0.499892

O 0.884347 0.598949 0.499936

O 0.036433 0.251600 0.499773

O 0.871665 0.432186 0.400873

O 0.218452 0.737601 0.400502

O 0.064911 0.583347 0.400705

O 0.897857 0.569150 0.299886

O 0.050690 0.260881 0.301055

H 0.958711 0.752983 0.698013

H 0.958230 0.257534 0.302034

H 0.851589 0.554440 0.730412

H 0.918014 0.496400 0.271747

O 0.210682 0.433346 0.699999

H 0.157797 0.505675 0.728251

O 0.217633 0.411403 0.304424

H 0.168068 0.435657 0.269809

Fe 0.551608 0.411829 0.658875

Fe 0.714528 0.584995 0.640167

Fe 0.718112 0.583885 0.463476

Fe 0.385198 0.751307 0.439026

Fe 0.385722 0.248738 0.563194

Fe 0.550682 0.417953 0.537950

Fe 0.551603 0.418074 0.360792

Fe 0.717461 0.588576 0.341998

O 0.555451 0.743618 0.698442

O 0.373811 0.585489 0.696380

O 0.703262 0.264758 0.599417

O 0.550660 0.569573 0.599066

O 0.394899 0.419369 0.598960

O 0.731633 0.402730 0.499729

O 0.384819 0.598686 0.499797

O 0.536552 0.250894 0.500145

O 0.371766 0.432088 0.400947

O 0.718315 0.737746 0.400568

O 0.564873 0.583558 0.400699

O 0.397709 0.569111 0.299830

O 0.550354 0.261068 0.301062

H 0.454712 0.757537 0.697433

H 0.457881 0.257747 0.302102

H 0.349865 0.558344 0.730701

H 0.418031 0.496108 0.271770

O 0.707534 0.437243 0.700982

H 0.651589 0.511881 0.728244

O 0.717272 0.411893 0.304305

H 0.667321 0.436750 0.269761

O 0.906841 0.058420 0.797559

O 0.154908 0.989719 0.797665

H 0.909570 0.145294 0.810240

H 0.149995 0.905547 0.812096

**Appendix 7. CIF file of (104) at the initial state (equation 6, and Fig. S13).**

_cell_length_a 9.0497

_cell_length_b 10.1802

_cell_length_c 25.4233

_cell_angle_alpha 90.0000

_cell_angle_beta 93.1170

_cell_angle_gamma 124.2259

_symmetry_space_group_name_H-M P1

loop_

_atom_site_type_symbol

_atom_site_fract_x

_atom_site_fract_y

_atom_site_fract_z

O 0.606600 0.086714 0.233081

Fe 0.875840 0.904988 0.336899

Fe 0.675678 0.047459 0.304057

Fe 0.175583 0.780415 0.304129

O 0.762129 0.992315 0.376565

O 0.793971 0.948250 0.268434

O 0.937274 0.766180 0.298022

O 0.121311 0.093358 0.344076

O 0.293656 0.998410 0.268449

O 0.107052 0.172853 0.233013

Fe 0.375654 0.123005 0.336892

O 0.261805 0.921247 0.376544

O 0.436937 0.823458 0.298042

O 0.621374 0.180020 0.344092

O 0.606621 0.586760 0.233076

Fe 0.875826 0.404983 0.336891

Fe 0.675675 0.547475 0.304053

Fe 0.175602 0.280423 0.304109

O 0.762140 0.492319 0.376563

O 0.793953 0.448234 0.268423

O 0.937304 0.266209 0.298019

O 0.121302 0.593347 0.344080

O 0.293664 0.498424 0.268433

O 0.107080 0.672899 0.233026

Fe 0.375654 0.622993 0.336884

O 0.261816 0.421248 0.376535

O 0.436951 0.323470 0.298034

O 0.621408 0.680061 0.344100

O 0.414446 0.731283 0.412456

Fe 0.655513 0.777114 0.426183

O 0.600656 0.077015 0.455201

Fe 0.846152 0.871962 0.550962

Fe 0.155666 0.028450 0.426266

Fe 0.860065 0.129876 0.440748

Fe 0.641845 0.019865 0.536569

Fe 0.141775 0.770570 0.536474

Fe 0.360007 0.880646 0.440651

O 0.739749 0.979282 0.600717

O 0.750643 0.923337 0.488609

O 0.900797 0.726384 0.522007

O 0.087300 0.066805 0.564786

O 0.250957 0.977029 0.488661

O 0.100951 0.173928 0.455245

O 0.914538 0.833671 0.412475

Fe 0.346300 0.123376 0.551051

O 0.239548 0.907918 0.600700

O 0.401304 0.823394 0.521940

O 0.587304 0.169174 0.564777

O 0.414483 0.231335 0.412455

Fe 0.655552 0.277158 0.426179

O 0.600645 0.576994 0.455208

Fe 0.846142 0.371975 0.550961

Fe 0.155654 0.528452 0.426268

Fe 0.860091 0.629900 0.440747

Fe 0.641811 0.519856 0.536564

Fe 0.141806 0.270612 0.536476

Fe 0.359976 0.380638 0.440646

O 0.739759 0.479284 0.600708

O 0.750653 0.423347 0.488606

O 0.900843 0.226455 0.522003

O 0.087302 0.566806 0.564791

O 0.250966 0.477040 0.488664

O 0.100993 0.673986 0.455247

O 0.914542 0.333675 0.412471

Fe 0.346330 0.623417 0.551058

O 0.239546 0.407918 0.600706

O 0.401288 0.323383 0.521943

O 0.587332 0.669214 0.564779

O 0.380032 0.720189 0.633034

Fe 0.625931 0.777491 0.640329

O 0.564344 0.076997 0.679133

Fe 0.125724 0.995572 0.640296

Fe 0.825815 0.120009 0.673044

Fe 0.325684 0.852986 0.673023

O 0.707402 0.902049 0.708746

O 0.893668 0.728179 0.744490

O 0.207275 0.952236 0.708732

O 0.063957 0.134134 0.679213

O 0.880220 0.807023 0.633011

O 0.394049 0.812876 0.744939

O 0.380057 0.220243 0.633035

Fe 0.625927 0.277497 0.640320

O 0.564355 0.577002 0.679137

Fe 0.125701 0.495548 0.640301

Fe 0.825839 0.620024 0.673029

Fe 0.325679 0.352996 0.673036

O 0.707408 0.402057 0.708734

O 0.895170 0.228002 0.745064

O 0.207250 0.452214 0.708739

O 0.063986 0.634166 0.679212

O 0.880211 0.307018 0.633021

O 0.394203 0.319548 0.747933

H 0.899536 0.159213 0.769450

H 0.886894 0.659530 0.770907

H 0.602213 0.373904 0.728018

H 0.602195 0.873885 0.728026

H 0.579979 0.109441 0.715474

H 0.580039 0.609445 0.715479

H 0.397985 0.400163 0.768124

H 0.383689 0.873848 0.770852

H 0.102033 0.375294 0.728032

H 0.102057 0.875311 0.728023

H 0.079615 0.117280 0.715544

H 0.079664 0.617347 0.715545

H 0.117917 0.743138 0.206531

H 0.117913 0.243090 0.206513

H 0.398827 0.526552 0.249129

H 0.398813 0.026531 0.249145

H 0.421561 0.791030 0.261710

H 0.421621 0.291036 0.261706

H 0.617352 0.027537 0.206479

H 0.617366 0.527579 0.206472

H 0.899079 0.025197 0.249107

H 0.899073 0.525194 0.249104

H 0.922177 0.783385 0.261692

H 0.922181 0.283397 0.261688

O 0.273959 0.142412 0.833019

H 0.314968 0.191320 0.798656

O 0.100791 0.122488 0.832407

H 0.133232 0.215654 0.852150

**Appendix 8. CIF file of (104) at the transition state (equation 6, and Fig. S13).**

_cell_length_a 9.0497

_cell_length_b 10.1802

_cell_length_c 25.4233

_cell_angle_alpha 90.0000

_cell_angle_beta 93.1170

_cell_angle_gamma 124.2259

_symmetry_space_group_name_H-M P1

loop_

_atom_site_type_symbol

_atom_site_fract_x

_atom_site_fract_y

_atom_site_fract_z

O 0.600887 0.005365 0.233595

Fe 0.870478 0.821707 0.337058

Fe 0.671300 -0.034972 0.304313

Fe 0.171291 0.698964 0.304336

O 0.757310 0.910291 0.377123

O 0.788979 0.865743 0.268792

O 0.931863 0.683188 0.297967

O 0.117525 0.011186 0.344952

O 0.288717 0.916049 0.268821

O 0.101581 0.088835 0.233527

Fe 0.370304 0.041371 0.337063

O 0.257320 0.839225 0.377104

O 0.431940 0.741360 0.297997

O 0.617614 0.099003 0.344968

O 0.600903 0.505397 0.233581

Fe 0.870444 0.321663 0.337056

Fe 0.671300 0.465046 0.304298

Fe 0.171309 0.198956 0.304320

O 0.757298 0.410284 0.377110

O 0.788978 0.365734 0.268787

O 0.931889 0.183201 0.297968

O 0.117499 0.511167 0.344951

O 0.288712 0.416049 0.268805

O 0.101616 0.588872 0.233537

Fe 0.370261 0.541346 0.337047

O 0.257293 0.339198 0.377090

O 0.431961 0.241371 0.297990

O 0.617622 0.599035 0.344963

O 0.410434 0.649843 0.412805

Fe 0.652327 0.696422 0.426230

O 0.598163 -0.003571 0.456230

Fe 0.843622 0.791851 0.552386

Fe 0.152456 -0.052982 0.426344

Fe 0.856338 0.048619 0.441824

Fe 0.639573 -0.060482 0.537282

Fe 0.139463 0.689947 0.536989

Fe 0.356539 0.799290 0.441738

O 0.738474 0.899141 0.601879

O 0.747850 0.842199 0.489400

O 0.897505 0.645484 0.522725

O 0.085609 -0.013268 0.566216

O 0.247922 0.896344 0.489491

O 0.098251 0.092936 0.456249

O 0.910513 0.752060 0.412805

Fe 0.343417 0.042050 0.552612

O 0.237915 0.828536 0.601725

O 0.397909 0.742319 0.522622

O 0.585303 0.088860 0.566123

O 0.410483 0.149898 0.412817

Fe 0.652399 0.196496 0.426277

O 0.598039 0.496315 0.456217

Fe 0.843596 0.291677 0.552579

Fe 0.152436 0.447064 0.426337

Fe 0.856316 0.548593 0.441780

Fe 0.639553 0.439221 0.537086

Fe 0.139533 0.190144 0.537139

Fe 0.356256 0.299091 0.441728

O 0.738508 0.399339 0.601814

O 0.747956 0.342262 0.489431

O 0.897389 0.145364 0.522730

O 0.085775 0.486768 0.566043

O 0.247916 0.396217 0.489460

O 0.098332 0.593123 0.456199

O 0.910542 0.252092 0.412812

Fe 0.344085 0.542234 0.552476

O 0.238314 0.327741 0.601849

O 0.397757 0.242169 0.522734

O 0.586239 0.588934 0.566142

O 0.379391 0.639202 0.633194

Fe 0.626511 0.697167 0.641747

O 0.564019 -0.002994 0.680734

Fe 0.125133 0.918408 0.641948

Fe 0.824244 0.041535 0.674972

Fe 0.326977 0.773907 0.673246

O 0.707386 0.822329 0.710269

O 0.894735 0.648532 0.745485

O 0.210175 0.874124 0.709936

O 0.062568 0.054471 0.681328

O 0.879077 0.727878 0.634143

O 0.392828 0.729839 0.744615

O 0.377980 0.139763 0.634377

Fe 0.624935 0.196309 0.642010

O 0.563937 0.496533 0.680761

Fe 0.125622 0.416845 0.641678

Fe 0.825123 0.539408 0.673904

Fe 0.322129 0.271968 0.675221

O 0.709722 0.324215 0.709858

O 0.898821 0.152542 0.746064

O 0.206680 0.373173 0.709996

O 0.065258 0.556983 0.680328

O 0.878260 0.227943 0.633631

O 0.397299 0.240618 0.748105

H 0.913493 0.086727 0.769786

H 0.883869 0.577845 0.771743

H 0.607617 0.298165 0.730053

H 0.599850 0.791977 0.729628

H 0.566908 0.025362 0.717654

H 0.577575 0.527478 0.717203

H 0.356809 0.260453 0.768433

H 0.394023 0.798804 0.769903

H 0.099731 0.296581 0.729397

H 0.107937 0.797778 0.730218

H 0.064721 0.028216 0.718159

H 0.081610 0.540736 0.716738

H 0.112799 0.657598 0.206524

H 0.112789 0.157561 0.206510

H 0.393702 0.444484 0.249289

H 0.393703 -0.055523 0.249305

H 0.417153 0.709185 0.261682

H 0.417202 0.209169 0.261679

H 0.612016 -0.051937 0.206448

H 0.612016 0.448081 0.206435

H 0.893879 -0.057828 0.249273

H 0.893891 0.442167 0.249274

H 0.917327 0.700796 0.261672

H 0.917319 0.200782 0.261672

O 0.367872 0.048721 0.810571

H 0.372806 0.103898 0.788325

O 0.081632 0.029058 0.810925

H 0.065961 0.046380 0.832496

**Appendix 9. CIF file of (104) at the end state (equation 6, and Fig. S13).**

_cell_length_a 9.0497

_cell_length_b 10.1802

_cell_length_c 25.4233

_cell_angle_alpha 90.0000

_cell_angle_beta 93.1170

_cell_angle_gamma 124.2259

_symmetry_space_group_name_H-M P1

loop_

_atom_site_type_symbol

_atom_site_fract_x

_atom_site_fract_y

_atom_site_fract_z

O 0.592325 0.883352 0.234361

Fe 0.862437 0.696789 0.337295

Fe 0.664734 0.841385 0.304695

Fe 0.164854 0.576790 0.304644

O 0.750090 0.787268 0.377956

O 0.781496 0.741993 0.269323

O 0.923752 0.558713 0.297880

O 0.111849 0.887938 0.346261

O 0.281313 0.792519 0.269373

O 0.093381 0.962819 0.234293

Fe 0.362281 0.918923 0.337319

O 0.250599 0.716203 0.377940

O 0.424450 0.618222 0.297924

O 0.611977 0.977485 0.346275

O 0.592332 0.383363 0.234335

Fe 0.862372 0.196688 0.337302

Fe 0.664738 0.341406 0.304664

Fe 0.164870 0.076757 0.304636

O 0.750043 0.287244 0.377925

O 0.781521 0.241994 0.269327

O 0.923773 0.058702 0.297887

O 0.111797 0.387908 0.346251

O 0.281290 0.292497 0.269358

O 0.093426 0.462843 0.234300

Fe 0.362174 0.418878 0.337291

O 0.250517 0.216135 0.377918

O 0.424482 0.118232 0.297919

O 0.611945 0.477505 0.346251

O 0.404424 0.527696 0.413323

Fe 0.647549 0.575386 0.426300

O 0.594425 0.875561 0.457769

Fe 0.839828 0.671687 0.554519

Fe 0.147641 0.824873 0.426460

Fe 0.850750 0.926736 0.443437

Fe 0.636167 0.819000 0.538349

Fe 0.135996 0.569015 0.537760

Fe 0.351340 0.677259 0.443366

O 0.736565 0.778940 0.603619

O 0.743666 0.720501 0.490581

O 0.892574 0.524147 0.523797

O 0.083078 0.866634 0.568355

O 0.243376 0.775330 0.490730

O 0.094206 0.971456 0.457749

O 0.904483 0.629656 0.413296

Fe 0.339095 0.920064 0.554951

O 0.235468 0.709473 0.603260

O 0.392824 0.620716 0.523640

O 0.582306 0.968400 0.568138

O 0.404489 0.027756 0.413356

Fe 0.647670 0.075507 0.426422

O 0.594132 0.375307 0.457724

Fe 0.839778 0.171234 0.555003

Fe 0.147611 0.324986 0.426440

Fe 0.850656 0.426635 0.443328

Fe 0.636167 0.318271 0.537868

Fe 0.136125 0.069444 0.538131

Fe 0.350677 0.176774 0.443348

O 0.736637 0.279434 0.603468

O 0.743916 0.220643 0.490664

O 0.892215 0.023741 0.523816

O 0.083488 0.366723 0.567917

O 0.243346 0.274996 0.490648

O 0.094344 0.471837 0.457621

O 0.904550 0.129730 0.413318

Fe 0.340718 0.420462 0.554602

O 0.236469 0.207486 0.603558

O 0.392468 0.120358 0.523916

O 0.584604 0.468526 0.568181

O 0.378438 0.517736 0.633430

Fe 0.627380 0.576685 0.643871

O 0.563537 0.877026 0.683128

Fe 0.124248 0.802664 0.644424

Fe 0.821888 0.923828 0.677862

Fe 0.328920 0.655292 0.673579

O 0.707371 0.702760 0.712548

O 0.896343 0.529074 0.746974

O 0.214530 0.756968 0.711739

O 0.060490 0.934988 0.684494

O 0.877371 0.609168 0.635838

O 0.391000 0.605295 0.744127

O 0.374870 0.019056 0.636386

Fe 0.623447 0.074530 0.644542

O 0.563316 0.375840 0.683191

Fe 0.125505 0.298792 0.643742

Fe 0.824050 0.418486 0.675215

Fe 0.316804 0.150429 0.678497

O 0.713201 0.207466 0.711539

O 0.904305 0.039364 0.747562

O 0.205831 0.254621 0.711876

O 0.067170 0.441219 0.681997

O 0.875341 0.109339 0.634543

O 0.401794 0.122296 0.748441

H 0.934398 0.978131 0.770194

H 0.879416 0.455477 0.772904

H 0.615749 0.184680 0.733010

H 0.596362 0.669275 0.731945

H 0.547401 0.899459 0.720893

H 0.573961 0.404715 0.719696

H 0.297656 0.050071 0.767597

H 0.409585 0.686354 0.768381

H 0.096387 0.178709 0.731349

H 0.116821 0.681617 0.733407

H 0.042479 0.894776 0.721995

H 0.084596 0.425960 0.718439

H 0.105217 0.529483 0.206429

H 0.105198 0.029461 0.206420

H 0.386119 0.321570 0.249445

H 0.386143 0.821582 0.249460

H 0.410635 0.586595 0.261555

H 0.410667 0.086546 0.261555

H 0.604107 0.829023 0.206317

H 0.604086 0.329003 0.206295

H 0.886184 0.817823 0.249438

H 0.886224 0.317814 0.249445

H 0.910146 0.577099 0.261557

H 0.910122 0.077046 0.261563

O 0.508741 0.908323 0.776847

H 0.459359 0.971174 0.773533

O 0.052089 0.889840 0.779836

H 0.978207 0.778427 0.785053

**Appendix 10. CIF file of (116) at the initial state (equation 7, and Fig. S17).**

_cell_length_a 7.4825

_cell_length_b 9.0497

_cell_length_c 27.0000

_cell_angle_alpha 110.1837

_cell_angle_beta 95.8323

_cell_angle_gamma 63.6274

_symmetry_space_group_name_H-M P1

loop_

_atom_site_type_symbol

_atom_site_fract_x

_atom_site_fract_y

_atom_site_fract_z

O 0.793646 0.318621 0.229279

O 0.448029 0.665785 0.245518

O 0.835433 0.921711 0.278854

O 0.167711 0.577771 0.267940

O 0.509912 0.208228 0.250344

Fe 0.352176 0.414430 0.305430

O 0.429069 0.350492 0.378330

O 0.533823 0.539532 0.332355

Fe 0.833140 0.403556 0.302498

Fe 0.612693 0.462469 0.400338

O 0.783537 0.331625 0.450504

O 0.133964 0.329519 0.313106

Fe 0.475711 0.772041 0.323632

O 0.456821 0.996408 0.311480

Fe 0.665315 0.785049 0.453461

Fe 0.970326 0.781428 0.328403

Fe 0.500695 0.118417 0.387200

Fe 0.119578 0.459859 0.400632

O 0.455697 0.690332 0.455733

O 0.168870 0.899488 0.333721

O 0.497666 0.910909 0.404687

O 0.826297 0.549605 0.399962

O 0.854599 0.892498 0.478088

O 0.121042 0.659586 0.379617

O 0.797483 0.017164 0.387023

O 0.789522 0.651803 0.303940

Fe 0.160604 0.785981 0.454437

O 0.154914 0.589821 0.477253

Fe 0.786383 0.125654 0.474341

Fe 0.993941 0.130328 0.392737

O 0.840804 0.226316 0.334745

O 0.491895 0.227091 0.472841

Fe 0.294216 0.122346 0.473481

O 0.191418 0.226297 0.408329

O 0.095838 0.023799 0.452587

Fe 0.318323 0.464236 0.525090

O 0.420796 0.361275 0.590445

O 0.515652 0.563718 0.545887

Fe 0.826081 0.461961 0.524378

Fe 0.617490 0.457999 0.605790

O 0.773116 0.359771 0.663068

O 0.120857 0.360060 0.525910

Fe 0.451907 0.801671 0.544400

O 0.458006 0.997646 0.521512

Fe 0.643962 0.806873 0.670502

Fe 0.947252 0.802664 0.545355

Fe 0.492890 0.127459 0.598198

Fe 0.111603 0.467897 0.611394

O 0.443433 0.689082 0.664576

O 0.156766 0.897765 0.543602

O 0.493056 0.927241 0.619230

O 0.814575 0.571050 0.611606

O 0.823796 0.940680 0.697169

O 0.113378 0.677606 0.594467

O 0.785979 0.037520 0.598801

O 0.758336 0.694985 0.520709

Fe 0.136277 0.815345 0.674586

O 0.156418 0.589223 0.686922

Fe 0.774867 0.189711 0.697880

Fe 0.997692 0.125888 0.598837

O 0.828337 0.256407 0.548245

O 0.476853 0.255853 0.684694

Fe 0.258583 0.173098 0.693868

O 0.182630 0.236051 0.620396

O 0.075328 0.050277 0.668192

O 0.104752 0.378526 0.749208

O 0.445270 0.008436 0.731053

O 0.176468 0.897797 0.754004

O 0.780127 0.664141 0.719227

O 0.776253 0.326207 0.768574

H 0.780410 0.292904 0.798984

H 0.213857 0.799756 0.765186

H 0.366705 0.974823 0.747401

H 0.746896 0.712258 0.756121

H 0.994745 0.376504 0.764286

H 0.045891 0.607666 0.708226

H 0.480894 0.666175 0.697095

H 0.394414 0.380365 0.693266

H 0.805898 0.454234 0.687617

H 0.830335 0.949590 0.733625

H 0.613265 0.215996 0.234440

H 0.806398 0.132825 0.310348

H 0.566618 0.978338 0.290051

H 0.866888 0.868574 0.241891

H 0.424974 0.740561 0.225719

H 0.251580 0.607748 0.251692

H 0.683906 0.408907 0.219353

H 0.129814 0.922424 0.301241

H 0.789761 0.634206 0.266874

H 0.216470 0.205365 0.305134

O 0.067820 0.120839 0.855585

H 0.095723 0.052403 0.817753

O 0.851523 0.221470 0.858148

H 0.808469 0.165097 0.875020

**Appendix 11. CIF file of (116) at the transition state (equation 7, and Fig. S17).**

_cell_length_a 7.4825

_cell_length_b 9.0497

_cell_length_c 27.0000

_cell_angle_alpha 110.1837

_cell_angle_beta 95.8323

_cell_angle_gamma 63.6274

_symmetry_space_group_name_H-M P1

loop_

_atom_site_type_symbol

_atom_site_fract_x

_atom_site_fract_y

_atom_site_fract_z

O 0.787818 0.323631 0.229242

O 0.443060 0.670597 0.245538

O 0.828355 0.926804 0.279048

O 0.161395 0.582678 0.267887

O 0.503770 0.213801 0.250062

Fe 0.345966 0.419272 0.305501

O 0.423396 0.354760 0.378184

O 0.527258 0.544612 0.332406

Fe 0.826993 0.408337 0.302668

Fe 0.606699 0.466905 0.400454

O 0.777199 0.335960 0.450559

O 0.127817 0.334031 0.313094

Fe 0.469688 0.777101 0.323944

O 0.450202 1.001580 0.311642

Fe 0.659201 0.789322 0.453673

Fe 0.964286 0.786146 0.328570

Fe 0.495058 0.123092 0.387367

Fe 0.113599 0.464395 0.400701

O 0.449643 0.694603 0.455782

O 0.162767 0.904330 0.333802

O 0.492097 0.915377 0.404819

O 0.820331 0.553756 0.399995

O 0.848296 0.896936 0.478191

O 0.114691 0.664046 0.379622

O 0.791854 0.021397 0.387059

O 0.783169 0.656758 0.304114

Fe 0.154797 0.790447 0.454566

O 0.148836 0.593972 0.476998

Fe 0.780331 0.130156 0.474515

Fe 0.988062 0.135008 0.392878

O 0.834347 0.231227 0.334871

O 0.485874 0.231873 0.472848

Fe 0.288550 0.126840 0.473601

O 0.185467 0.230933 0.408345

O 0.090167 0.028203 0.452594

Fe 0.311682 0.469665 0.525222

O 0.415933 0.364939 0.590482

O 0.510109 0.568453 0.546353

Fe 0.819798 0.466318 0.524446

Fe 0.610837 0.463366 0.606373

O 0.766107 0.364529 0.663400

O 0.114540 0.363503 0.525694

Fe 0.445945 0.806621 0.545046

O 0.451928 1.002232 0.521793

Fe 0.641491 0.809375 0.670975

Fe 0.941160 0.806760 0.545250

Fe 0.487263 0.131932 0.598330

Fe 0.106132 0.471875 0.610941

O 0.438769 0.695082 0.666602

O 0.151008 0.902476 0.543575

O 0.487735 0.932207 0.620189

O 0.808533 0.575756 0.611974

O 0.822804 0.943586 0.698090

O 0.108595 0.675651 0.591635

O 0.780502 0.041765 0.599297

O 0.751448 0.699656 0.520870

Fe 0.131190 0.814689 0.670964

O 0.151774 0.593022 0.686457

Fe 0.771870 0.189713 0.696660

Fe 0.992248 0.130313 0.598794

O 0.822349 0.261196 0.548479

O 0.471484 0.260425 0.685199

Fe 0.253360 0.176651 0.693384

O 0.177364 0.240470 0.620448

O 0.071740 0.055983 0.668358

O 0.101076 0.379639 0.750580

O 0.441483 0.012730 0.731676

O 0.111011 0.786042 0.788753

O 0.777431 0.666234 0.719587

O 0.776094 0.323138 0.768042

H 0.804951 0.267327 0.794178

H 0.153575 0.664590 0.780397

H 0.363233 0.987421 0.750265

H 0.748202 0.716282 0.756635

H 0.990788 0.372169 0.763775

H 0.037407 0.613664 0.706766

H 0.475656 0.667243 0.698254

H 0.389465 0.384719 0.693085

H 0.799446 0.458288 0.688146

H 0.833084 0.950860 0.734314

H 0.607559 0.221921 0.234492

H 0.800203 0.137482 0.310495

H 0.560253 0.983175 0.290229

H 0.860247 0.873959 0.242109

H 0.419824 0.746101 0.225997

H 0.245615 0.612745 0.251824

H 0.677610 0.414623 0.219676

H 0.124138 0.927003 0.301293

H 0.782957 0.639225 0.267058

H 0.210558 0.209907 0.305254

O 0.120775 0.055953 0.806638

H 0.131349 -0.165608 0.824928

O 0.926053 0.115134 0.822842

H 0.921217 0.032144 0.836726

**Appendix 12. CIF file of (116) at the end state (equation 7, and Fig. S17).**

_cell_length_a 7.4825

_cell_length_b 9.0497

_cell_length_c 27.0000

_cell_angle_alpha 110.1837

_cell_angle_beta 95.8323

_cell_angle_gamma 63.6274

_symmetry_space_group_name_H-M P1

loop_

_atom_site_type_symbol

_atom_site_fract_x

_atom_site_fract_y

_atom_site_fract_z

O 0.780646 0.329983 0.229378

O 0.438117 0.676115 0.245636

O 0.821828 0.932927 0.279253

O 0.155124 0.588685 0.267989

O 0.496924 0.219925 0.249816

Fe 0.339455 0.424533 0.305419

O 0.416713 0.360085 0.378214

O 0.521165 0.549700 0.332513

Fe 0.820617 0.413565 0.302671

Fe 0.600241 0.471909 0.400436

O 0.771071 0.340681 0.450634

O 0.121629 0.338975 0.313032

Fe 0.463302 0.782319 0.324017

O 0.442895 0.007384 0.311673

Fe 0.652460 0.794262 0.453759

Fe 0.957860 0.791373 0.328577

Fe 0.488727 0.128288 0.387352

Fe 0.107102 0.469334 0.400621

O 0.443347 0.699533 0.455883

O 0.156891 0.908937 0.333835

O 0.486057 0.920628 0.404937

O 0.813741 0.559037 0.400207

O 0.841657 0.902228 0.478337

O 0.108454 0.668909 0.379655

O 0.785559 0.026455 0.387057

O 0.776558 0.662467 0.304315

Fe 0.148708 0.795380 0.454570

O 0.142365 0.598966 0.477093

Fe 0.774031 0.135202 0.474532

Fe 0.981680 0.140015 0.392846

O 0.827361 0.236673 0.334937

O 0.479440 0.236824 0.472810

Fe 0.282319 0.131609 0.473579

O 0.178860 0.235979 0.408300

O 0.083721 0.033290 0.452641

Fe 0.306359 0.474202 0.525133

O 0.410343 0.369608 0.590373

O 0.503596 0.574271 0.546685

Fe 0.812913 0.471552 0.524548

Fe 0.605108 0.468743 0.606787

O 0.759519 0.369225 0.664049

O 0.108185 0.368558 0.525554

Fe 0.439496 0.812518 0.545644

O 0.445031 0.007402 0.522010

Fe 0.637344 0.815165 0.672442

Fe 0.934805 0.811938 0.545132

Fe 0.481332 0.137051 0.598432

Fe 0.099736 0.475343 0.610585

O 0.433909 0.699667 0.666660

O 0.144570 0.907282 0.543390

O 0.480666 0.938094 0.620933

O 0.802459 0.580274 0.612010

O 0.819442 0.944403 0.696891

O 0.104734 0.682308 0.592606

O 0.774381 0.046369 0.599431

O 0.744715 0.705242 0.521181

Fe 0.127342 0.820614 0.672759

O 0.144757 0.595388 0.685258

Fe 0.766777 0.190827 0.696177

Fe 0.986047 0.135441 0.598487

O 0.815443 0.266385 0.548530

O 0.465149 0.265935 0.685605

Fe 0.250874 0.177411 0.692867

O 0.171971 0.244863 0.620500

O 0.066475 0.058027 0.666271

O 0.094662 0.378776 0.751498

O 0.437205 0.020104 0.732059

O 0.059068 0.689859 0.816519

O 0.774938 0.669182 0.719908

O 0.786321 0.306347 0.769090

H 0.835565 0.233055 0.790399

H 0.083490 0.571428 0.792520

H 0.357373 0.996139 0.751387

H 0.748325 0.721769 0.757099

H 0.985346 0.366934 0.763471

H 0.030733 0.619529 0.706496

H 0.462789 0.675881 0.699258

H 0.383710 0.390324 0.693306

H 0.792272 0.463747 0.688699

H 0.843023 0.952304 0.734215

H 0.601059 0.228149 0.234414

H 0.793602 0.142583 0.310540

H 0.553226 0.988734 0.290314

H 0.853088 0.880184 0.242281

H 0.414403 0.751910 0.226162

H 0.239340 0.618362 0.251816

H 0.670717 0.421121 0.219806

H 0.118200 0.931951 0.301361

H 0.775685 0.644947 0.267239

H 0.204350 0.214860 0.305269

O 0.175516 0.904538 0.751420

H 0.135229 0.674580 0.846080

O 0.004999 0.953035 0.784823

H 0.043988 0.850126 0.796091

**Appendix 13. CIF file of (001) at the initial state (equation 7, and Fig. S17).**

_cell_length_a 10.1955

_cell_length_b 10.1955

_cell_length_c 24.2762

_cell_angle_alpha 90.0000

_cell_angle_beta 90.0000

_cell_angle_gamma 60.0000

_symmetry_space_group_name_H-M P1

loop_

_atom_site_type_symbol

_atom_site_fract_x

_atom_site_fract_y

_atom_site_fract_z

Fe 0.457745 0.814081 0.659732

Fe 0.621319 0.984894 0.640910

Fe 0.624087 0.983747 0.463687

Fe 0.290555 0.150468 0.439098

Fe 0.290758 0.650772 0.562516

Fe 0.457110 0.817934 0.538081

Fe 0.456738 0.816467 0.361210

Fe 0.622270 0.986833 0.342301

O 0.464064 0.143080 0.699638

O 0.280296 0.987281 0.697020

O 0.608607 0.665734 0.599534

O 0.457156 0.969923 0.599996

O 0.301220 0.818697 0.600503

O 0.638566 0.802884 0.500843

O 0.290165 0.999042 0.501084

O 0.442467 0.651090 0.500815

O 0.277042 0.831226 0.401883

O 0.623040 0.136449 0.401702

O 0.470326 0.982221 0.401633

O 0.302986 0.969246 0.301551

O 0.456156 0.660084 0.301803

H 0.367072 0.148821 0.699424

H 0.363392 0.657135 0.302314

H 0.261720 0.962713 0.733247

H 0.322494 0.902462 0.271343

O 0.620042 0.832226 0.700545

H 0.571851 0.897495 0.731055

O 0.623303 0.808525 0.305940

H 0.579918 0.826270 0.270264

Fe 0.956847 0.814961 0.659783

Fe 0.123553 0.985007 0.640573

Fe 0.124040 0.983511 0.463685

Fe 0.790656 0.150540 0.439131

Fe 0.790872 0.651019 0.562605

Fe 0.956960 0.818011 0.538393

Fe 0.956740 0.816467 0.361205

Fe 0.122261 0.986867 0.342289

O 0.960429 0.145055 0.699661

O 0.778739 0.991811 0.696235

O 0.109525 0.666318 0.600834

O 0.957867 0.970187 0.600463

O 0.801659 0.819141 0.600418

O 0.138347 0.802802 0.501029

O 0.790512 0.998904 0.501126

O 0.942751 0.651084 0.501047

O 0.777113 0.831266 0.401885

O 0.122971 0.136458 0.401677

O 0.970291 0.982221 0.401639

O 0.802995 0.969255 0.301557

O 0.956178 0.660049 0.301819

H 0.860508 0.157482 0.697756

H 0.863443 0.657049 0.302317

H 0.754307 0.971585 0.731731

H 0.822480 0.902538 0.271332

O 0.106787 0.845211 0.705046

H 0.043024 0.937002 0.724909

O 0.123425 0.808434 0.305965

H 0.080164 0.826016 0.270271

Fe 0.460515 0.314522 0.659169

Fe 0.625033 0.484633 0.639470

Fe 0.623776 0.483928 0.463644

Fe 0.290456 0.650458 0.439069

Fe 0.290910 0.151046 0.562665

Fe 0.457306 0.317679 0.537914

Fe 0.456902 0.316391 0.361259

Fe 0.622363 0.486856 0.342269

O 0.469746 0.636088 0.698548

O 0.284220 0.495802 0.695670

O 0.609436 0.164291 0.600091

O 0.459005 0.469748 0.599399

O 0.303015 0.319194 0.600101

O 0.638863 0.302707 0.500883

O 0.290075 0.499136 0.501003

O 0.442804 0.150649 0.500883

O 0.277154 0.331255 0.401915

O 0.622934 0.636494 0.401623

O 0.470354 0.482271 0.401647

O 0.303165 0.469411 0.301623

O 0.456174 0.160262 0.301835

H 0.379099 0.629373 0.698017

H 0.363252 0.157612 0.302354

H 0.260172 0.477020 0.731480

H 0.322707 0.402860 0.271339

O 0.628073 0.327807 0.698517

H 0.586251 0.389889 0.730212

O 0.623633 0.308308 0.306015

H 0.580471 0.325641 0.270310

Fe 0.960881 0.315146 0.659438

Fe 0.124279 0.485246 0.640293

Fe 0.123683 0.483884 0.463690

Fe 0.790437 0.650580 0.439077

Fe 0.790886 0.150532 0.562751

Fe 0.957074 0.317764 0.537941

Fe 0.956842 0.316475 0.361255

Fe 0.122443 0.486823 0.342293

O 0.967909 0.638876 0.699533

O 0.783718 0.495432 0.694591

O 0.110207 0.164506 0.600506

O 0.959381 0.470024 0.599587

O 0.804161 0.318545 0.599703

O 0.138779 0.302685 0.500945

O 0.790465 0.499070 0.500862

O 0.942970 0.150433 0.500953

O 0.777145 0.331268 0.401890

O 0.122920 0.636468 0.401653

O 0.970315 0.482346 0.401663

O 0.803139 0.469434 0.301616

O 0.956099 0.160290 0.301838

H 0.873604 0.638802 0.698888

H 0.863196 0.157609 0.302353

H 0.754404 0.485178 0.730648

H 0.822710 0.402867 0.271335

O 0.126679 0.329678 0.699549

H 0.082225 0.393779 0.730608

O 0.123722 0.308251 0.306038

H 0.080656 0.325539 0.270319

O 0.247395 0.894433 0.811000

O 0.219849 0.766954 0.809436

H 0.159819 0.967047 0.830552

H 0.191585 0.771176 0.770735

**Appendix 14. CIF file of (001) at the transition state (equation 7, and Fig. S17).**

_cell_length_a 10.1955

_cell_length_b 10.1955

_cell_length_c 24.2762

_cell_angle_alpha 90.0000

_cell_angle_beta 90.0000

_cell_angle_gamma 60.0000

_symmetry_space_group_name_H-M P1

loop_

_atom_site_type_symbol

_atom_site_fract_x

_atom_site_fract_y

_atom_site_fract_z

Fe 0.458624 0.814514 0.659325

Fe 0.622934 0.984468 0.640859

Fe 0.624993 0.983680 0.463570

Fe 0.291536 0.150458 0.438952

Fe 0.291431 0.650487 0.562288

Fe 0.458079 0.817871 0.537852

Fe 0.457880 0.816364 0.361116

Fe 0.623401 0.986706 0.342187

O 0.464440 0.142851 0.699557

O 0.279469 0.985895 0.696473

O 0.609557 0.665649 0.599541

O 0.457877 0.970124 0.599906

O 0.301993 0.818741 0.600193

O 0.639418 0.802802 0.500659

O 0.291129 0.999079 0.500866

O 0.443378 0.650936 0.500611

O 0.278166 0.831198 0.401730

O 0.624195 0.136317 0.401522

O 0.471408 0.982164 0.401471

O 0.304112 0.969299 0.301410

O 0.457210 0.660027 0.301683

H 0.367334 0.149057 0.699228

H 0.364419 0.657119 0.302248

H 0.259273 0.959373 0.732375

H 0.323718 0.902521 0.271200

O 0.620794 0.832078 0.700435

H 0.573557 0.898993 0.730586

O 0.624777 0.808098 0.305852

H 0.581581 0.825586 0.270155

Fe 0.956887 0.816436 0.658674

Fe 0.123105 0.986912 0.639423

Fe 0.124960 0.983474 0.463550

Fe 0.791694 0.150450 0.438978

Fe 0.791784 0.651102 0.562612

Fe 0.957678 0.817796 0.538294

Fe 0.957852 0.816373 0.361108

Fe 0.123383 0.986765 0.342157

O 0.962578 0.141438 0.698946

O 0.774604 0.996075 0.695535

O 0.110843 0.666120 0.600807

O 0.959167 0.970066 0.600243

O 0.801782 0.819311 0.599919

O 0.139227 0.802660 0.500766

O 0.791434 0.998892 0.500904

O 0.943673 0.650793 0.500897

O 0.778235 0.831196 0.401759

O 0.124120 0.136340 0.401494

O 0.971379 0.982170 0.401459

O 0.804099 0.969300 0.301423

O 0.957203 0.659999 0.301696

H 0.862473 0.153955 0.696963

H 0.864440 0.657043 0.302254

H 0.757427 0.971004 0.731501

H 0.823673 0.902586 0.271195

O -0.004029 0.833603 0.742358

H -0.090282 0.894408 0.764106

O 0.124884 0.808024 0.305868

H 0.081815 0.825325 0.270154

Fe 0.461394 0.314058 0.658920

Fe 0.625385 0.484966 0.639483

Fe 0.624833 0.483826 0.463520

Fe 0.291542 0.650402 0.438915

Fe 0.292024 0.151224 0.562496

Fe 0.458128 0.317672 0.537832

Fe 0.458045 0.316291 0.361142

Fe 0.623529 0.486742 0.342156

O 0.470148 0.636998 0.698445

O 0.284691 0.495216 0.695760

O 0.610371 0.164268 0.599969

O 0.459785 0.469726 0.599331

O 0.303800 0.319391 0.599953

O 0.639708 0.302627 0.500698

O 0.291090 0.499044 0.500801

O 0.443610 0.150683 0.500711

O 0.278285 0.331208 0.401765

O 0.624149 0.636354 0.401465

O 0.471482 0.482170 0.401464

O 0.304300 0.469523 0.301488

O 0.457216 0.160201 0.301706

H 0.377953 0.633110 0.698327

H 0.364238 0.157647 0.302275

H 0.261107 0.476273 0.731610

H 0.323901 0.403047 0.271182

O 0.628413 0.328009 0.698528

H 0.585912 0.390143 0.730143

O 0.625179 0.307879 0.305912

H 0.582231 0.324944 0.270182

Fe 0.961223 0.315032 0.659248

Fe 0.123989 0.486194 0.640586

Fe 0.124672 0.483836 0.463557

Fe 0.791518 0.650498 0.439000

Fe 0.791434 0.150828 0.562537

Fe 0.957953 0.317904 0.537844

Fe 0.957976 0.316410 0.361147

Fe 0.123590 0.486724 0.342176

O 0.968777 0.635777 0.699609

O 0.784275 0.494569 0.694908

O 0.110710 0.165621 0.600092

O 0.959841 0.470171 0.599723

O 0.804624 0.318719 0.599628

O 0.139601 0.302721 0.500744

O 0.791344 0.499029 0.500768

O 0.943768 0.150492 0.500786

O 0.778289 0.331219 0.401756

O 0.124100 0.636333 0.401476

O 0.971391 0.482284 0.401498

O 0.804276 0.469553 0.301486

O 0.957123 0.160270 0.301704

H 0.873534 0.638959 0.698618

H 0.864156 0.157698 0.302272

H 0.755566 0.481999 0.730758

H 0.823922 0.403044 0.271187

O 0.127260 0.328870 0.699479

H 0.082907 0.392745 0.730615

O 0.125239 0.307846 0.305939

H 0.082351 0.324882 0.270200

O 0.202607 0.895732 0.790652

O 0.207145 0.771860 0.764680

H 0.101033 0.979943 0.796107

H 0.072670 0.763874 0.771001

**Appendix 15. CIF file of (001) at the end state (equation 7, and Fig. S17).**

_cell_length_a 10.1955

_cell_length_b 10.1955

_cell_length_c 24.2762

_cell_angle_alpha 90.0000

_cell_angle_beta 90.0000

_cell_angle_gamma 60.0000

_symmetry_space_group_name_H-M P1

loop_

_atom_site_type_symbol

_atom_site_fract_x

_atom_site_fract_y

_atom_site_fract_z

Fe 0.459552 0.815150 0.659074

Fe 0.624412 0.984708 0.641071

Fe 0.626619 0.983473 0.463281

Fe 0.293314 0.150281 0.438638

Fe 0.292973 0.650120 0.562019

Fe 0.459508 0.817630 0.537519

Fe 0.459888 0.816089 0.360865

Fe 0.625402 0.986387 0.341892

O 0.465799 0.142223 0.699086

O 0.280832 0.986535 0.696901

O 0.611579 0.665319 0.599514

O 0.459538 0.970286 0.599728

O 0.304731 0.818874 0.598744

O 0.641155 0.802525 0.500441

O 0.292779 0.999127 0.500500

O 0.445030 0.650494 0.500260

O 0.280102 0.831072 0.401514

O 0.626277 0.135992 0.401249

O 0.473353 0.981937 0.401239

O 0.306096 0.969219 0.301208

O 0.459090 0.659815 0.301540

H 0.368457 0.148844 0.698975

H 0.366264 0.656965 0.302090

H 0.256338 0.953795 0.730303

H 0.325824 0.902584 0.270932

O 0.621943 0.831996 0.699977

H 0.577115 0.900706 0.729887

O 0.627247 0.807394 0.305708

H 0.584446 0.824416 0.269946

Fe 0.957387 0.817281 0.659189

Fe 0.126125 0.985275 0.638490

Fe 0.126684 0.983313 0.463252

Fe 0.793525 0.150214 0.438640

Fe 0.793192 0.650737 0.562366

Fe 0.959350 0.817603 0.537769

Fe 0.959820 0.816109 0.360852

Fe 0.125372 0.986486 0.341833

O 0.962841 0.140406 0.698175

O 0.782423 0.991154 0.696236

O 0.111615 0.665853 0.600474

O 0.960209 0.969910 0.599288

O 0.804094 0.818845 0.600010

O 0.140799 0.802291 0.500377

O 0.793001 0.998686 0.500560

O 0.945210 0.650319 0.500723

O 0.780188 0.831001 0.401580

O 0.126176 0.136044 0.401212

O 0.973315 0.981960 0.401209

O 0.806052 0.969204 0.301231

O 0.959046 0.659794 0.301549

H 0.864037 0.150146 0.695618

H 0.866241 0.656906 0.302100

H 0.769018 0.966830 0.733273

H 0.825733 0.902638 0.270937

O 0.829520 0.831569 0.801319

H 0.761813 0.798745 0.812778

O 0.127330 0.807349 0.305713

H 0.084660 0.824146 0.269933

Fe 0.462854 0.313739 0.658892

Fe 0.625812 0.485465 0.639589

Fe 0.626651 0.483554 0.463230

Fe 0.293509 0.650163 0.438584

Fe 0.293933 0.151198 0.562141

Fe 0.459734 0.317615 0.537635

Fe 0.460058 0.316017 0.360858

Fe 0.625581 0.486443 0.341862

O 0.470556 0.638230 0.698572

O 0.286313 0.492166 0.695678

O 0.612129 0.163775 0.599794

O 0.461257 0.469539 0.599088

O 0.304802 0.319953 0.599668

O 0.641231 0.302362 0.500404

O 0.292853 0.498796 0.500524

O 0.445090 0.150667 0.500467

O 0.280235 0.331065 0.401554

O 0.626323 0.636015 0.401228

O 0.473496 0.481875 0.401195

O 0.306299 0.469529 0.301294

O 0.459079 0.159983 0.301549

H 0.375700 0.639333 0.698324

H 0.366023 0.157569 0.302096

H 0.262633 0.475660 0.731846

H 0.325964 0.403299 0.270892

O 0.629218 0.328415 0.698331

H 0.586013 0.389917 0.730059

O 0.627747 0.307189 0.305748

H 0.585239 0.323754 0.269947

Fe 0.961935 0.314895 0.658934

Fe 0.126811 0.485544 0.640662

Fe 0.126483 0.483620 0.463244

Fe 0.793407 0.650289 0.438750

Fe 0.792598 0.151023 0.562122

Fe 0.959645 0.317760 0.537605

Fe 0.959981 0.316187 0.360876

Fe 0.125618 0.486451 0.341874

O 0.968197 0.641919 0.701513

O 0.785844 0.493544 0.695396

O 0.112504 0.166018 0.599845

O 0.960904 0.470771 0.600177

O 0.805813 0.318866 0.599548

O 0.141027 0.302692 0.500469

O 0.792973 0.498850 0.500655

O 0.945263 0.150508 0.500546

O 0.780269 0.331062 0.401570

O 0.126203 0.636008 0.401215

O 0.973311 0.482067 0.401271

O 0.806278 0.469568 0.301300

O 0.958954 0.160116 0.301541

H 0.874797 0.640369 0.698566

H 0.865900 0.157699 0.302090

H 0.757728 0.477153 0.730837

H 0.826013 0.403281 0.270911

O 0.127564 0.328967 0.699108

H 0.084713 0.390421 0.730852

O 0.127758 0.307193 0.305780

H 0.085260 0.323754 0.269976

O 0.103682 0.880115 0.752742

O 0.133597 0.822713 0.696674

H 0.036453 0.988159 0.745360

H 0.898026 0.754227 0.776526

**Appendix 16. CIF file of (104) at the initial state (equation 7, and Fig. S17).**

_cell_length_a 9.0497

_cell_length_b 10.1802

_cell_length_c 25.4233

_cell_angle_alpha 90.0000

_cell_angle_beta 93.1170

_cell_angle_gamma 124.2259

_symmetry_space_group_name_H-M P1

loop_

_atom_site_type_symbol

_atom_site_fract_x

_atom_site_fract_y

_atom_site_fract_z

O 0.303298 0.585563 0.230473

Fe 0.572469 0.400086 0.334461

Fe 0.374430 0.544583 0.301109

Fe 0.874403 0.279656 0.301110

O 0.460809 0.490662 0.375286

O 0.492220 0.445603 0.266225

O 0.633887 0.261952 0.294811

O 0.822151 0.591235 0.343097

O 0.991760 0.496295 0.266201

O 0.803439 0.667560 0.230455

Fe 0.072391 0.622034 0.334503

O 0.959934 0.419637 0.375289

O 0.133803 0.321515 0.294840

O 0.322081 0.680424 0.343126

O 0.303305 0.085560 0.230474

Fe 0.572430 0.900037 0.334471

Fe 0.374454 0.044627 0.301121

Fe 0.874391 0.779621 0.301117

O 0.460746 0.990558 0.375316

O 0.492197 0.945601 0.266239

O 0.633886 0.761937 0.294813

O 0.822140 0.091239 0.343088

O 0.991729 0.996226 0.266193

O 0.803418 0.167562 0.230455

Fe 0.072324 0.121994 0.334491

O 0.959919 0.919609 0.375291

O 0.133823 0.821540 0.294857

O 0.322075 0.180456 0.343117

O 0.113268 0.230567 0.410800

Fe 0.356949 0.278895 0.424000

O 0.302996 0.578593 0.455408

Fe 0.545815 0.373419 0.552933

Fe 0.856631 0.527888 0.424077

Fe 0.559066 0.629280 0.441018

Fe 0.343429 0.521516 0.536228

Fe 0.843547 0.272174 0.536209

Fe 0.059475 0.379960 0.440959

O 0.443745 0.482158 0.601729

O 0.451425 0.423686 0.488487

O 0.599660 0.226805 0.521696

O 0.789536 0.568956 0.566418

O 0.951357 0.477948 0.488633

O 0.803180 0.674601 0.455455

O 0.613261 0.332347 0.410778

Fe 0.045449 0.622160 0.553152

O 0.942913 0.411470 0.601962

O 0.099826 0.323021 0.521715

O 0.289062 0.670560 0.566445

O 0.113228 0.730550 0.410823

Fe 0.356959 0.778990 0.424035

O 0.302974 0.078456 0.455368

Fe 0.545322 0.873069 0.553086

Fe 0.856643 0.027978 0.424074

Fe 0.559182 0.129280 0.441047

Fe 0.343363 0.021476 0.536199

Fe 0.843312 0.771910 0.536090

Fe 0.059402 0.879898 0.440968

O 0.442636 0.981428 0.601897

O 0.451245 0.923509 0.488579

O 0.599562 0.726900 0.521727

O 0.789082 0.068606 0.566230

O 0.951214 0.977724 0.488606

O 0.803256 0.174713 0.455429

O 0.613269 0.832398 0.410793

Fe 0.045436 0.122493 0.553004

O 0.942262 0.910511 0.602024

O 0.099799 0.822845 0.521905

O 0.288975 0.170709 0.566049

O 0.079851 0.220815 0.633842

Fe 0.329598 0.279176 0.642250

O 0.269445 0.580127 0.682786

Fe 0.828989 0.500054 0.642686

Fe 0.528330 0.621778 0.676431

Fe 0.028805 0.356847 0.676594

O 0.410393 0.404270 0.710412

O 0.596403 0.234543 0.745694

O 0.911451 0.457024 0.711064

O 0.769008 0.638810 0.682879

O 0.579664 0.309333 0.633624

O 0.100081 0.316049 0.746942

O 0.080156 0.720567 0.634675

Fe 0.329911 0.778813 0.642763

O 0.267370 0.078775 0.681956

Fe 0.829490 0.001502 0.642591

Fe 0.527613 0.121348 0.675888

Fe 0.026432 0.856097 0.675585

O 0.410919 0.904755 0.711068

O 0.601809 0.734312 0.746802

O 0.909883 0.955465 0.710664

O 0.768662 0.139989 0.682379

O 0.579882 0.809870 0.634096

O 0.099515 0.822970 0.749337

H 0.614750 0.675375 0.773588

H 0.588230 0.173028 0.774819

H 0.309072 0.879236 0.731284

H 0.308338 0.377275 0.731142

H 0.285379 0.611461 0.719267

H 0.285563 0.114624 0.718012

H 0.101125 0.899111 0.771493

H 0.087110 0.369315 0.775101

H 0.803472 0.878490 0.730172

H 0.807587 0.382602 0.731374

H 0.778898 0.618711 0.719253

H 0.782137 0.121662 0.718713

H 0.814168 0.234862 0.202944

H 0.814200 0.734913 0.202962

H 0.096033 0.024313 0.246318

H 0.096041 0.524362 0.246318

H 0.119000 0.289238 0.258509

H 0.119029 0.789251 0.258527

H 0.314034 0.529206 0.202898

H 0.314042 0.029148 0.202916

H 0.596442 0.521732 0.246302

H 0.596423 0.021735 0.246319

H 0.618998 0.279466 0.258489

H 0.619015 0.779480 0.258492

O 0.946346 0.613940 0.827833

H 0.002669 0.679066 0.797294

O 0.797453 0.631186 0.831724

H 0.836264 0.696421 0.863085

**Appendix 17. CIF file of (104) at the transition state (equation 7, and Fig. S17).**

_cell_length_a 9.0497

_cell_length_b 10.1802

_cell_length_c 25.4233

_cell_angle_alpha 90.0000

_cell_angle_beta 93.1170

_cell_angle_gamma 124.2259

_symmetry_space_group_name_H-M P1

loop_

_atom_site_type_symbol

_atom_site_fract_x

_atom_site_fract_y

_atom_site_fract_z

O 0.303627 0.584738 0.230545

Fe 0.572849 0.398981 0.334686

Fe 0.374851 0.543462 0.301302

Fe 0.874782 0.278676 0.301360

O 0.461133 0.489497 0.375422

O 0.492721 0.444542 0.266338

O 0.634090 0.260772 0.294924

O 0.822446 0.590220 0.343202

O 0.992112 0.495146 0.266335

O 0.804509 0.666427 0.230562

Fe 0.072787 0.621029 0.334682

O 0.960375 0.418718 0.375432

O 0.134248 0.320556 0.294988

O 0.322502 0.679297 0.343234

O 0.303662 0.084775 0.230554

Fe 0.572824 0.898940 0.334669

Fe 0.374878 0.043514 0.301320

Fe 0.874793 0.778669 0.301359

O 0.461156 0.989479 0.375436

O 0.492696 0.944552 0.266336

O 0.634077 0.760755 0.294910

O 0.822461 0.090246 0.343207

O 0.992110 0.995130 0.266337

O 0.804493 0.166454 0.230559

Fe 0.072852 0.121069 0.334688

O 0.960388 0.918697 0.375388

O 0.134275 0.820577 0.294997

O 0.322534 0.179316 0.343256

O 0.113872 0.229811 0.410902

Fe 0.357405 0.277848 0.424275

O 0.303648 0.577432 0.455544

Fe 0.546856 0.372517 0.553200

Fe 0.857156 0.526942 0.424297

Fe 0.559738 0.628308 0.441212

Fe 0.344372 0.520399 0.536470

Fe 0.844547 0.271390 0.536383

Fe 0.060028 0.379027 0.441204

O 0.445148 0.481539 0.602244

O 0.452064 0.422432 0.488752

O 0.600232 0.225607 0.521991

O 0.790395 0.568062 0.566714

O 0.951856 0.477068 0.488794

O 0.803623 0.673537 0.455487

O 0.613628 0.331414 0.410967

Fe 0.046233 0.621493 0.553367

O 0.943617 0.410110 0.602161

O 0.100853 0.322193 0.521886

O 0.290384 0.669943 0.566438

O 0.113776 0.729715 0.410892

Fe 0.357330 0.777775 0.424225

O 0.303749 0.077475 0.455576

Fe 0.547062 0.872431 0.553043

Fe 0.857137 0.027045 0.424249

Fe 0.559771 0.128290 0.441225

Fe 0.344104 0.020556 0.536457

Fe 0.844153 0.771258 0.536298

Fe 0.059923 0.878951 0.441145

O 0.444462 0.981571 0.602416

O 0.451807 0.922436 0.488709

O 0.599833 0.725311 0.521791

O 0.789632 0.068077 0.566028

O 0.951984 0.976956 0.488483

O 0.803757 0.173755 0.455517

O 0.613633 0.831444 0.410938

Fe 0.046166 0.120857 0.553149

O 0.941255 0.911383 0.600327

O 0.100945 0.821961 0.521824

O 0.290718 0.170315 0.566515

O 0.082048 0.220612 0.634025

Fe 0.330355 0.277553 0.642851

O 0.271220 0.579866 0.682736

Fe 0.831789 0.501946 0.643055

Fe 0.531158 0.621583 0.676333

Fe 0.029987 0.356497 0.675936

O 0.411465 0.404173 0.711000

O 0.600656 0.235030 0.746536

O 0.914636 0.457090 0.710966

O 0.772409 0.639180 0.684082

O 0.581631 0.309423 0.634205

O 0.099826 0.315472 0.747325

O 0.083072 0.718055 0.634742

Fe 0.330251 0.776833 0.641909

O 0.270384 0.076452 0.683221

Fe 0.830440 0.000472 0.642351

Fe 0.529927 0.123155 0.675779

Fe 0.025496 0.858595 0.673292

O 0.412731 0.903298 0.711797

O 0.600655 0.733763 0.747555

O 0.907462 0.956506 0.709175

O 0.769375 0.140142 0.682085

O 0.580176 0.808499 0.634150

O 0.067835 0.921766 0.784445

H 0.606758 0.669249 0.773408

H 0.587869 0.170524 0.775204

H 0.317038 0.878485 0.732796

H 0.308050 0.376905 0.731170

H 0.286887 0.610808 0.719227

H 0.282857 0.110827 0.719112

H 0.099955 1.000618 0.810216

H 0.088488 0.373464 0.774218

H 0.806096 0.879652 0.729802

H 0.811198 0.383069 0.731497

H 0.783882 0.621466 0.720547

H 0.785390 0.122769 0.718369

H 0.814843 0.234012 0.203192

H 0.814872 0.733980 0.203192

H 0.096533 0.023247 0.246505

H 0.096530 0.523247 0.246502

H 0.118983 0.287982 0.258667

H 0.119055 0.788023 0.258676

H 0.314160 0.527907 0.203087

H 0.314207 0.027908 0.203111

H 0.597162 0.520750 0.246513

H 0.597137 0.020770 0.246511

H 0.619129 0.278493 0.258630

H 0.619137 0.778479 0.258614

O 1.011200 0.665275 0.798310

H -0.034637 0.830542 0.797803

O 0.845725 0.641202 0.817034

H 0.884254 0.721551 0.845014

**Appendix 18. CIF file of (104) at the end state (equation 7, and Fig. S17).**

_cell_length_a 9.0497

_cell_length_b 10.1802

_cell_length_c 25.4233

_cell_angle_alpha 90.0000

_cell_angle_beta 93.1170

_cell_angle_gamma 124.2259

_symmetry_space_group_name_H-M P1

loop_

_atom_site_type_symbol

_atom_site_fract_x

_atom_site_fract_y

_atom_site_fract_z

O 0.303821 0.582940 0.230733

Fe 0.572940 0.396898 0.334881

Fe 0.375044 0.541470 0.301372

Fe 0.874897 0.276677 0.301513

O 0.461250 0.487295 0.375622

O 0.493375 0.442760 0.266523

O 0.634144 0.258845 0.295046

O 0.822498 0.588328 0.343419

O 0.992571 0.493139 0.266553

O 0.805646 0.664510 0.230811

Fe 0.073009 0.619139 0.334808

O 0.960792 0.417068 0.375674

O 0.134697 0.318651 0.295163

O 0.322725 0.677317 0.343424

O 0.303900 0.083029 0.230759

Fe 0.572942 0.896910 0.334828

Fe 0.375079 0.041528 0.301405

Fe 0.874932 0.776718 0.301502

O 0.461363 0.987377 0.375621

O 0.493357 0.942788 0.266494

O 0.634109 0.758822 0.295009

O 0.822566 0.088377 0.343426

O 0.992610 0.993198 0.266569

O 0.805656 0.164579 0.230801

Fe 0.073270 0.119288 0.334844

O 0.960830 0.917047 0.375591

O 0.134731 0.818657 0.295164

O 0.322808 0.177331 0.343490

O 0.114327 0.228110 0.411099

Fe 0.357759 0.275911 0.424561

O 0.304347 0.575375 0.455750

Fe 0.548091 0.370861 0.553709

Fe 0.857534 0.525027 0.424411

Fe 0.559758 0.626152 0.441282

Fe 0.345627 0.518633 0.536908

Fe 0.845296 0.269963 0.536478

Fe 0.060608 0.377341 0.441371

O 0.446790 0.480204 0.602716

O 0.452824 0.420283 0.489099

O 0.601090 0.223835 0.522257

O 0.791293 0.566653 0.567048

O 0.952081 0.475179 0.488944

O 0.804029 0.671819 0.455652

O 0.613928 0.329767 0.411245

Fe 0.047668 0.620200 0.553496

O 0.944719 0.408971 0.602275

O 0.101885 0.320799 0.522095

O 0.291668 0.668539 0.566509

O 0.114163 0.727909 0.411028

Fe 0.357539 0.775626 0.424353

O 0.304515 0.075629 0.455843

Fe 0.547427 0.870426 0.552954

Fe 0.857450 0.025272 0.424327

Fe 0.560142 0.126365 0.441349

Fe 0.345211 0.018895 0.536747

Fe 0.844827 0.768905 0.535980

Fe 0.060788 0.877387 0.441221

O 0.446325 0.980443 0.602376

O 0.452525 0.920505 0.488771

O 0.600830 0.723322 0.521899

O 0.791262 0.066175 0.565948

O 0.952591 0.975338 0.488659

O 0.804234 0.172103 0.455696

O 0.613952 0.829725 0.411171

Fe 0.048551 0.120243 0.553111

O 0.944680 0.909572 0.601244

O 0.101637 0.820482 0.521813

O 0.292102 0.168626 0.567074

O 0.083743 0.218817 0.634040

Fe 0.332855 0.276771 0.643606

O 0.273364 0.579059 0.682486

Fe 0.833067 0.500613 0.643703

Fe 0.533621 0.620585 0.676768

Fe 0.032596 0.356188 0.675517

O 0.411974 0.403537 0.711566

O 0.606272 0.234906 0.747078

O 0.918500 0.457720 0.711328

O 0.776247 0.640017 0.684959

O 0.583523 0.308850 0.634927

O 0.098745 0.315043 0.747376

O 0.083539 0.718693 0.633357

Fe 0.333356 0.777591 0.642725

O 0.271642 0.075892 0.683022

Fe 0.830751 0.999838 0.641957

Fe 0.531950 0.121414 0.676976

Fe 0.029228 0.855930 0.674326

O 0.420798 0.904159 0.711345

O 0.600821 0.732403 0.747962

O 0.910464 0.954481 0.709642

O 0.772909 0.140509 0.681558

O 0.582804 0.806710 0.633899

O 0.977820 0.978065 0.842395

H 0.598434 0.663235 0.773528

H 0.586149 0.167654 0.775529

H 0.322791 0.873886 0.733665

H 0.307822 0.375811 0.731377

H 0.288170 0.610241 0.718923

H 0.276701 0.100413 0.719750

H 0.046986 0.075836 0.861928

H 0.090289 0.378219 0.772907

H 0.807580 0.877407 0.729606

H 0.815780 0.383320 0.731861

H 0.797211 0.631973 0.722295

H 0.789128 0.124180 0.717923

H 0.815418 0.231981 0.203449

H 0.815451 0.731823 0.203421

H 0.096903 0.021325 0.246677

H 0.096885 0.521261 0.246669

H 0.118675 0.285804 0.258838

H 0.118806 0.785886 0.258835

H 0.313939 0.525906 0.203251

H 0.314026 0.025985 0.203282

H 0.597842 0.518837 0.246714

H 0.597811 0.018883 0.246684

H 0.618995 0.276655 0.258773

H 0.618996 0.776604 0.258731

O 0.119478 0.836272 0.746714

H 0.874571 0.972392 0.827503

O 0.980202 0.740515 0.782126

H 0.990452 0.823092 0.804162
